# Cumulative microscopy reveals cellular states in fibroblasts from patients with genetic disorders

**DOI:** 10.1038/s44319-026-00859-5

**Published:** 2026-07-30

**Authors:** Karen Akopyan, Zhiyu Hao, Susann Karlberg, Svetlana Vakkilainen, Fulya Taylan, Minna Pekkinen, Outi Mäkitie, Arne Lindqvist

**Affiliations:** 1 Department of Cell and Molecular Biology, Karolinska Institutet, Stockholm, Sweden; 2 Children’s Hospital, Pediatric Research Center, University of Helsinki and Helsinki University Hospital, Helsinki, Finland; 3 Research Program for Clinical and Molecular Metabolism, Faculty of Medicine, University of Helsinki, Helsinki, Finland; 4 Folkhälsan Research Center, Institute of Genetics, Helsinki, Finland; 5 Department of Molecular Medicine and Surgery, Karolinska Institutet, and Department of Clinical Genetics and Genomics, Karolinska University Hospital, Stockholm, Sweden

**Keywords:** DNA Replication, Recombination & Repair, Methods & Resources, Molecular Biology of Disease

## Abstract

Analysis of cellular states and signaling trajectories can provide insights into causes of disease. We developed cumulative microscopy, a method to perform cyclical imaging without elution or quenching steps. Cumulative microscopy computationally extracts individual signals from accumulating fluorescence during sequential imaging. We use cumulative microscopy to quantitatively assess cell cycle and stress markers in individual primary fibroblasts from patients with rare genetic proliferative disorders with increased cancer risk. Neural network-based analysis of cumulative microscopy data suggests that cells from patients with Cartilage-hair hypoplasia (CHH), but not Mulibrey Nanism (MUL), show replication stress. We analyze cell states and cell trajectories and find that a subset of cells from patients with CHH show spontaneous replication stress, followed by cell cycle exit in both G1 and G2 phases. We note that replication stress potentially could underlie both proliferative defects and increased cancer risk in CHH patients and conclude that cumulative microscopy is an efficient, quantitative, and generalizable approach to multiplex microscopy.

## Synopsis

This study develops cumulative microscopy, a method to multiplex immunofluorescence that does not need elution or quenching steps. It detects cellular states and spontaneous replication stress in cells from patients with Cartilage-hair hypoplasia (CHH).

- This method multiplexes microscopy by addition of stainings without the need for elution or quenching and can be performed with off-the shelf reagents.
- It allows the assessment of cellular states.
- Cells from patients with Cartilage-hair hypoplasia (CHH) show spontaneous replication stress.

## Abstract (web summary)

This study develops cumulative microscopy, a method to multiplex immunofluorescence that does not need elution or quenching steps. It detects cellular states and spontaneous replication stress in cells from patients with Cartilage-hair hypoplasia (CHH).

## Graphical abstract

**Figure:**
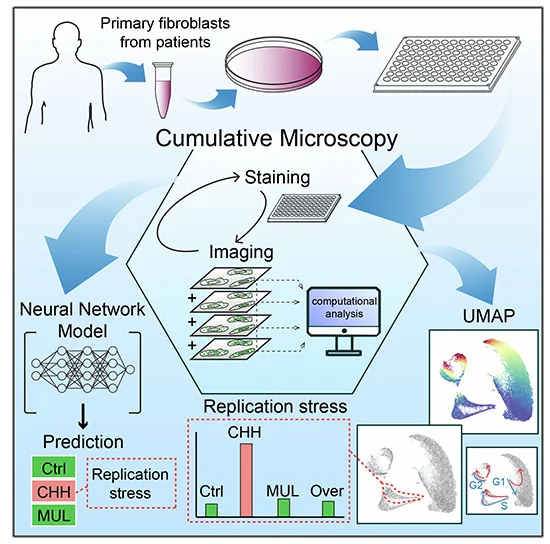

**Figure:**
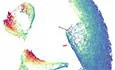

## Introduction

The cell forms the basic unit of life. The collective action of cells impacts on a wide range of processes in health and disease. To understand and model cellular behavior, a large number of different parameters can be described that collectively outline various cellular states (Rafelski and Theriot, 2024). Transitions between cellular states can be used to model eventual cell fates, while also accounting for various responses in a heterogenous population of cells (Rafelski and Theriot, 2024; Movasat et al, 2025).

Several thousand rare genetic disorders have been identified, but understanding of the mechanistic connection between genotype and phenotype remains often incomplete (Haendel et al, 2020). These include cartilage-hair hypoplasia (CHH; OMIM #250250) and Mulibrey Nanism (MUL; OMIM #253250). Both CHH and MUL are very rare autosomal recessive disorders that display a range of phenotypes, including growth failure. Whereas CHH is caused by mutations in the non-coding RNA *RMRP* that forms part of a large RNA processing complex (Ridanpää et al, 2001), MUL is caused by mutations in the E3 ubiquitin ligase TRIM37 (Avela et al, 2000; Kallijärvi et al, 2005). Both CHH and MUL patients have increased cancer risk, and expression of *RMRP* and TRIM37 is implicated in cancer progression (Hao and Zhou, 2022; Przanowski et al, 2020).

*RMRP* dysfunction affects the expression of cell cycle-regulating genes (Vakkilainen et al, 2019; Gao et al, 2025; Sun et al, 2019; Thiel et al, 2005), p53 protein level (Chen et al, 2021), and ribosome synthesis (Robertson et al, 2022), but how pathogenic variants of *RMRP* cause proliferative defects and increased cancer risk remains unclear. Despite reports on increased duration of the late cell cycle (Vakkilainen et al, 2019; Gill et al, 2004), a characterization of functional cellular states based on protein levels and signaling status is lacking.

Analysis of signaling trajectories requires multiplexed information from single cells. Due to the large number of available antibodies, immunofluorescence can detect a wide selection of proteins and post-translational modifications. In addition to the advantage of subcellular resolution, we have previously shown that immunofluorescence can be quantitative and be used to estimate temporal signaling dynamics through the cell cycle (Akopyan et al, 2014). However, quantitative immunofluorescence is restricted by the number of different antibodies that can be used on one cell. Increasing the number of antibodies allows for establishing signaling trajectories and has been used to determine signaling status and trajectories through the cell cycle (Stallaert et al, 2022a; Gookin et al, 2017; Mahdessian et al, 2021).

Different approaches have been used to allow the detection of more than one stain in fluorescence microscopy. A combination of antibodies from different species, followed by secondary antibodies coupled to different fluorophores, is routinely used. More recently, sequential or cyclical imaging has been established (Hickey et al, 2022). These methods rely on blocking the signal after imaging to allow another round of staining and imaging. Various methods have been established, including bleaching fluorophores (Schubert et al, 2006; Radtke et al, 2020), labeling antibodies with DNA barcodes (Goltsev et al, 2018; Saka et al, 2019), and elution of bound antibodies (Lan et al, 1995; Pirici et al, 2009). Methods that depend on bleaching and DNA barcodes require labeling of primary antibodies, excluding the use of off-the-shelf antibodies and amplification by secondary antibodies. Antibody elution balances efficient antibody removal with loss of epitopes (Klevanski et al, 2020), and requires strategies to minimize imaging-induced cross-linking of antibodies (Gut et al, 2018).

We have developed cumulative microscopy, a method that relies on sequential imaging, which retains the signal of previous stainings and therefore does not require bleaching, quenching, or elution steps. We find that cumulative microscopy is quantitative and can support neural network-based analysis as well as identification of cellular states and trajectories between states. Using cumulative microscopy on fibroblasts from patients with rare genetic disorders, we find that a subset of fibroblasts from CHH patients show spontaneous replication stress. We note that replication stress potentially could explain both proliferation defects and cancer prevalence in CHH patients.

## Results and discussion

**Figure 1:**
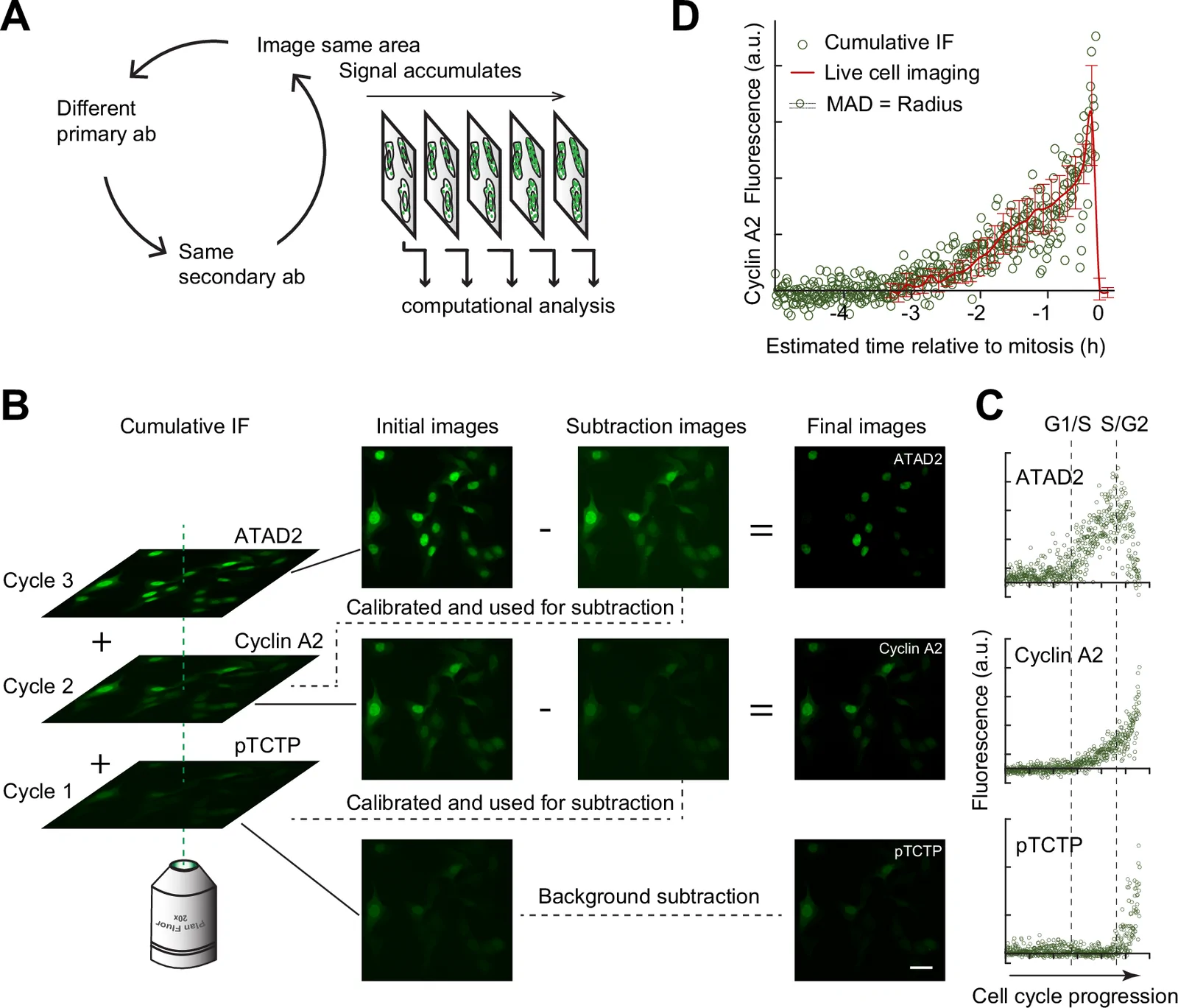
Cumulative microscopy. (**A**) Schematic of cumulative microscopy. (**B**) Workflow for cumulative microscopy. Images to the right show resulting subtractions after cumulative staining with ATAD2, Cyclin A2, and pTCTP antibodies. Scale bar 40 μm. (**C**) Quantification after imaging data as in (**B**), with the difference that estimation of the scaling function and subtraction are performed on measurements of nuclei rather than on full images. Graphs show measurements for individual cells sorted in silico for cell cycle progression. Data shown are representative of at least three independent experiments, each performed with technical replicates. (**D**) Comparison of Cyclin A2 levels based on live-cell imaging of Cyclin A2-YFP gene-targeted RPE cells (red line) and time-estimated Cyclin A2 levels in RPE cells based on data from (**C**) (green circles). The size of the circles indicates estimated technical variability (mean absolute deviation, MAD). Data are representative of two independent experiments with a total of four technical replicates. Source data are available online for this figure.

**Figure EV1:**
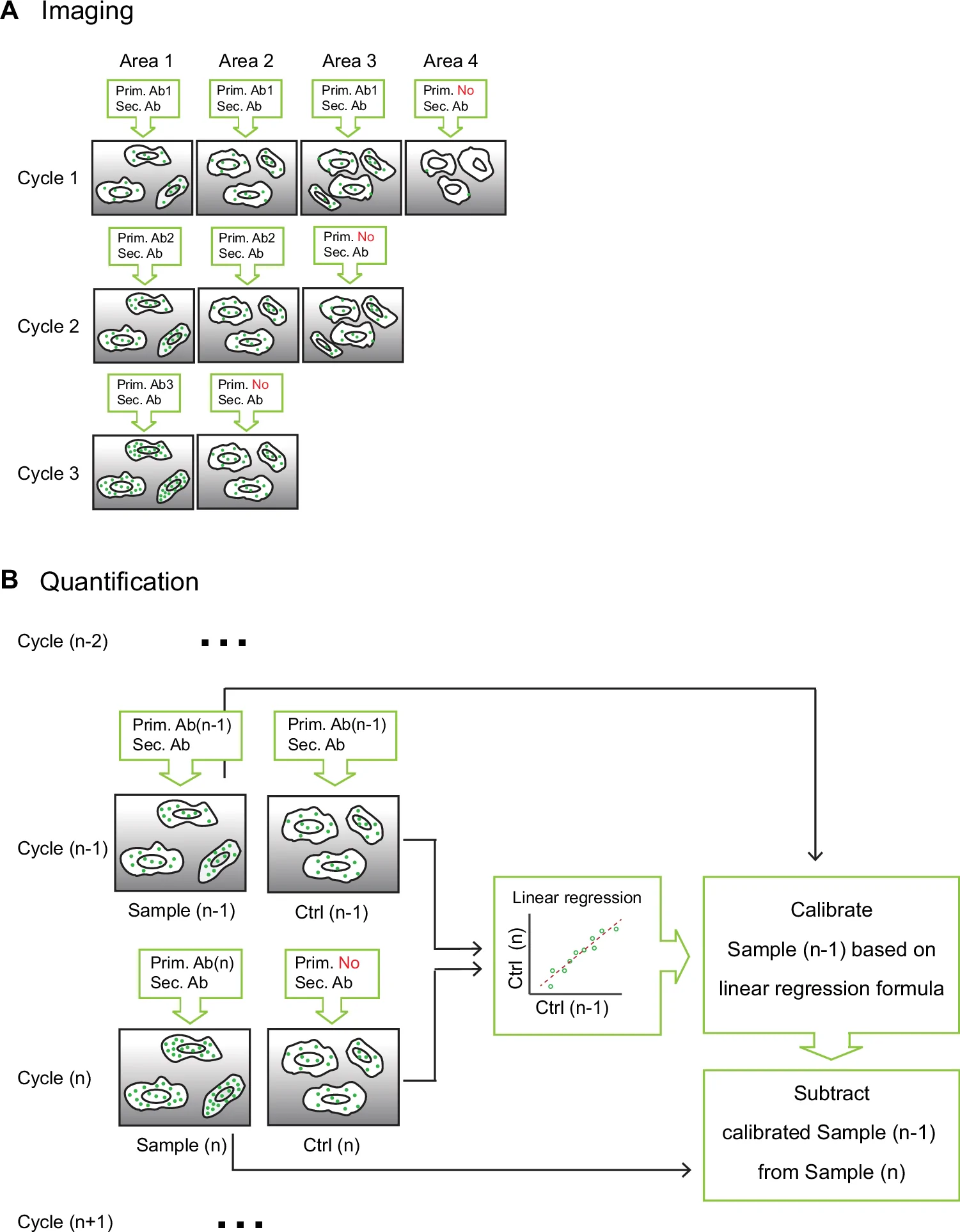
Principles of cumulative microscopy. (**A**) Schematic of the imaging setup. Each imaging cycle requires an extra staining in which no primary antibody is present. (**B**) Schematic of the quantification setup. Pixel values or quantification of segmented structures from the same area are compared for two imaging cycles. A scaling function is estimated by linear regression for the area in which no new primary antibody is introduced (Ctrl). The scaling function is used to calibrate the image or measurements from the sample before it can be subtracted from the image or measurements of the following cycle.

We developed cumulative microscopy, a method to multiplex fluorescence microscopy by computationally extracting individual signals from sequential imaging. During consecutive imaging cycles, different primary antibodies from the same species are used with the same secondary antibody. The resulting images show an additive staining over each imaging cycle. To retrieve the signal of one specific antibody, the image of the previous imaging cycle is subtracted (Fig. 1A). To control for bleaching and additive binding of secondary antibodies, the subtraction is calibrated by control images in which the last primary antibody is omitted (Fig. EV1). Due to the calibration step, exposure conditions can be changed between imaging cycles, avoiding eventual saturation of the signal.

The subtractions in consecutive imaging cycles can either be performed directly on the images or on measurements from the images (Fig. 1A,B; Appendix Fig. S1). From a practical perspective, we find that the latter can more easily correct for a shift in position that may occur if the same specimen is repeatedly replaced in a microscope, and therefore allows processing without accurate image registration. Further, using cytometric measurements rather than pixel values reduces variability that could depend on minimal shifts in pixel position, as well as reducing computational processing needed (Appendix Fig. S1). We conclude that cumulative microscopy can function both directly on images and on cytometric measurements from images, but is particularly suited for the latter.

**Figure EV2:**
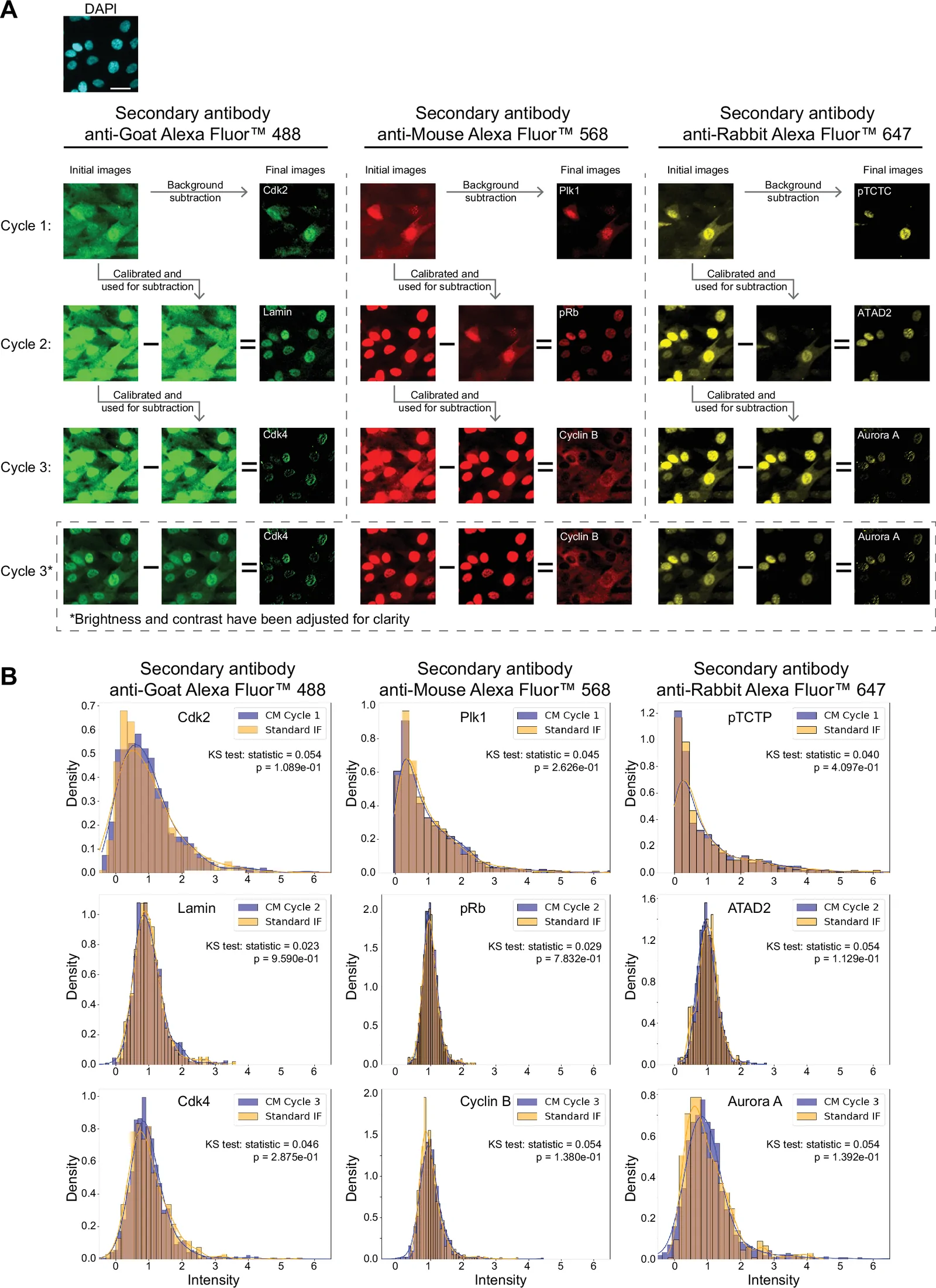
Workflow for cumulative microscopy. (**A**) Example images for cumulative staining with Cdk2, Lamin, Cdk4, Plk1, pRb, Cyclin B, ATAD2, Aurora A, and pTCTP antibodies. Scale bar 40 μm. (**B**) Comparison of signal intensity distributions between Standard IF and three sequential cycles of Cumulative IF in RPE G2 cells. Each Cumulative IF cycle included simultaneous staining with three secondary antibodies: anti-Goat Alexa Fluor™ 488, anti-Mouse Alexa Fluor™ 568, and anti-Rabbit Alexa Fluor™ 647. Statistical differences in signal distributions were evaluated using the Kolmogorov–Smirnov (KS) test. Data shown are representative of three independent experiments, each performed with technical replicates.

Next, we used cytometric measurements to assess the nuclear signals of pTCTP, Cyclin A2, and ATAD2, three targets that show similar nuclear localization, but distinct patterns of expression through the cell cycle (Fig. 1C). We estimated cell cycle progression based on DNA content and Cyclin A2 levels (Akopyan et al, 2014), and note that ATAD2 levels declined heavily through G2 phase (Lafranchi et al, 2020). In contrast, the preceding stainings of pTCTP and Cyclin A2 increased through G2 phase and reached their peak expression at the time when ATAD2 returned to baseline signal, indicating that stainings for Cyclin A2 and pTCTP did not contribute significantly to the resolved ATAD2 signal (Fig. 1C). Similarly, using an image-based approach, a cytoplasmic staining is apparent, despite that preceding nuclear stainings have stronger intensity (Fig. EV2A). Further, we find that the expression patterns are similar between cumulative microscopy and one round of quantitative immunofluorescence in both RPE1 cells and in primary fibroblasts (Appendix Fig. S2). This quantitative correlation between cumulative microscopy and one round of quantitative immunofluorescence also exists when three different secondary antibodies coupled to different fluorophores are used in the same sample (Fig. EV2B).

**Figure EV3:**
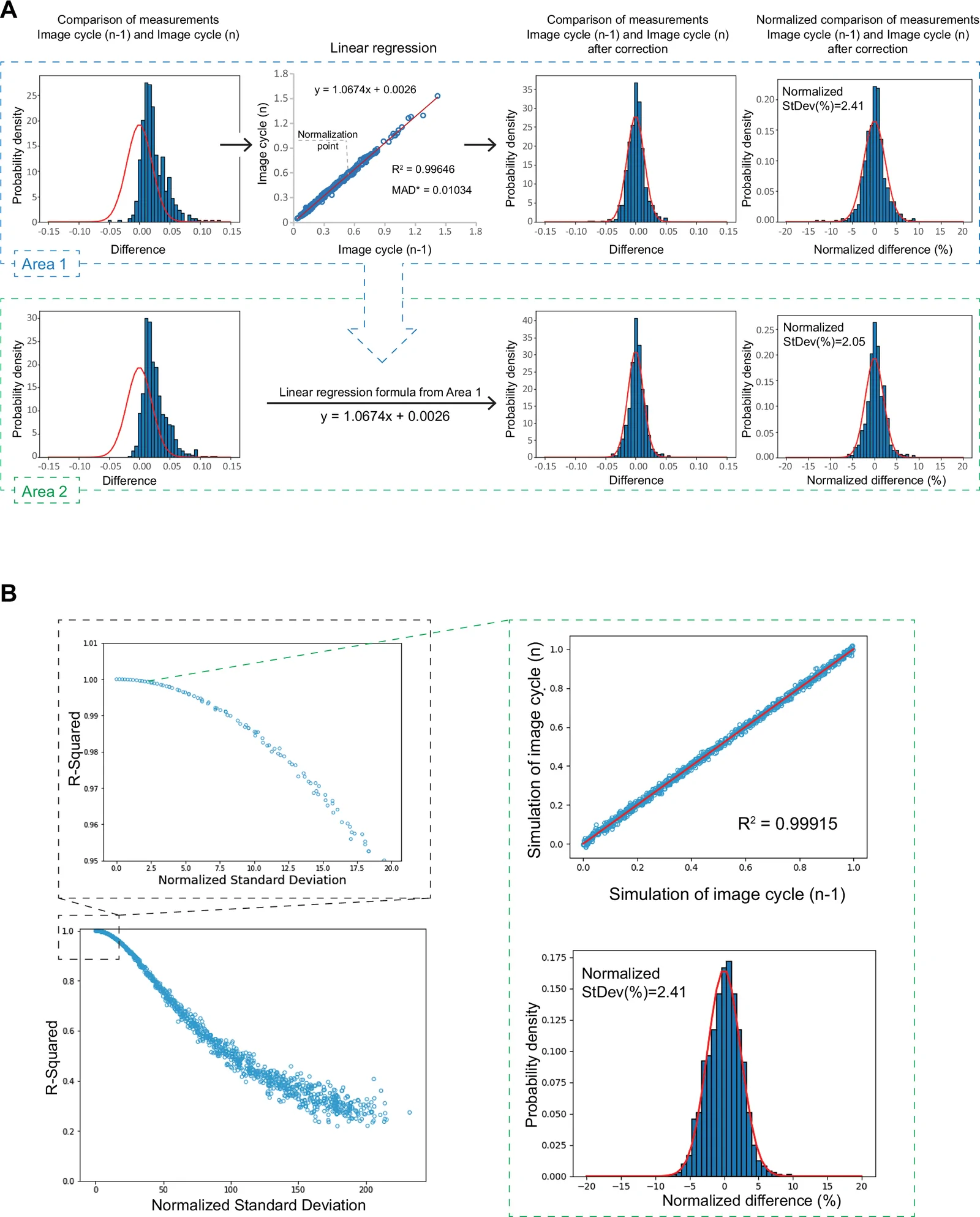
Estimation of uncertainty introduced by cumulative microscopy. (**A**) Histograms show a comparison between measurements from two consecutive imaging cycles in which no new primary antibody is introduced. The red line shows a normal distribution centered at 0 with the standard deviation derived from the data as a comparison. Left, comparison of raw data. Middle, linear regression, and the resulting distribution after correction by the scaling function. Right, the same data after normalization to signal strength. Lower graphs show regions from a second well, using the scaling function derived from the first well. *The mean absolute deviation (MAD) is used to represent the average distance between each data value and the regression line. (**B**) Simulation of the relation between the R-square value and the normalized standard deviation.

We developed a quantitative estimate of the uncertainty introduced by cumulative microscopy that is readily accessible from the calibration of each imaging cycle. Briefly, the quantitative estimate is derived from the difference in corrected measurements of two successive image cycles in a sample in which no new primary antibody is introduced (Fig. EV3). We note that the technical variability introduced by cumulative microscopy, indicated as the size of circles in Fig. 1D, can be small compared to variation between cells as assessed both by live-cell imaging and quantitative immunofluorescence (Fig. 1C,D; Appendix Fig. S2).

Both the expression pattern over time and variation between cells are similar between cumulative microscopy for Cyclin A2 and live-cell imaging of Cyclin A2-YFP (Fig. 1D). To assess how technical variability is affected by varying exposure conditions, we performed parallel experiments with different exposures. We find that subtle changes in exposure do not impact the estimate of technical variability. In addition, we do not detect a statistical difference between parallel samples (Appendix Fig. S3). Taken together, these data indicate that cumulative microscopy can be used to quantitatively assess individual antibody stainings. For a more detailed discussion of the quantitative estimate of variability and possible error sources, please see the Appendix.

**Figure 2:**
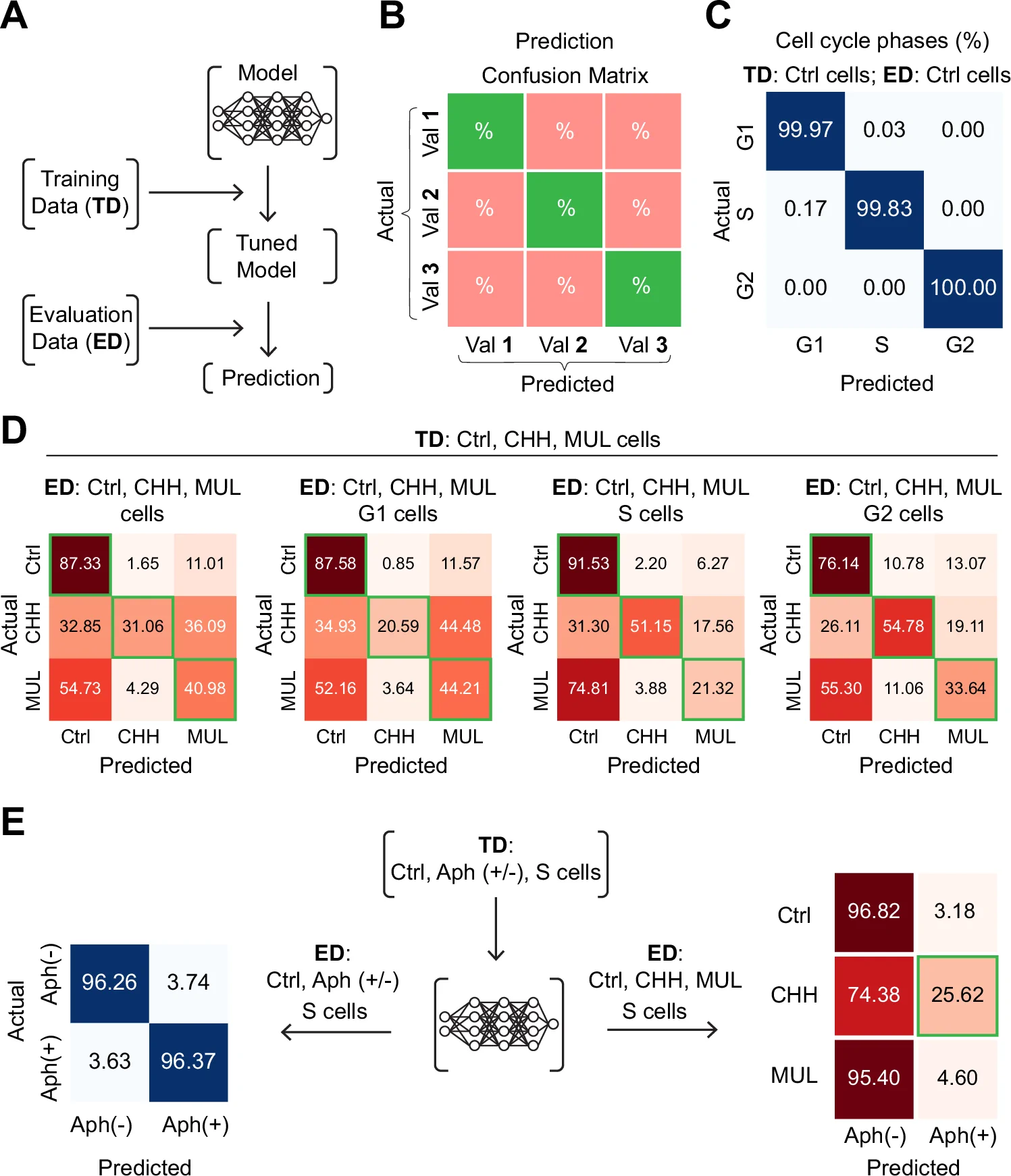
Neural network-based analysis of cumulative microscopy data. (**A**) Schematic of analysis. (**B**) Schematic of confusion matrix for visualization of results. (**C**) Model learning and prediction of cell cycle phase based on cumulative microscopy data on untreated control cells. (**D**) Model learning and prediction of cell type based on cumulative microscopy data on untreated control, CHH, and MUL cells. While model learning was performed for all cells, model prediction was performed either on all cells or on separate datasets. The left matrix shows the prediction for all cells, and the remaining matrices show the prediction separated for individual cell cycle phases. (**E**) The left matrix shows learning and prediction based on control S-phase cells treated with or without aphidicolin. Right matrix shows learning based on control S phase cells treated with or without aphidicolin, and prediction using untreated control, CHH, and MUL S phase cells. Source data are available online for this figure.

We used cumulative microscopy on a collection of primary fibroblasts from individuals with monogenic growth disorders with cell proliferative defects and from matched controls (Table EV1). We chose the antibodies to detect key proteins regulating cell cycle progression and stress response (Appendix Fig. S2D). Whereas Cyclin A2 increases from the G1/S transition, Plk1 increases mainly in G2 phase, and the Plk1 target phosphorylation pTCTP increases from the S/G2 transition until mitosis. Similarly, γH2AX was used to mark damaged DNA, and the DNA-damage-responsive transcription factor p53 and its transcriptional target p21 were used to indicate a stress response. At the same time, we recorded DAPI intensity to estimate DNA content, as well as nuclear area and EdU incorporation, the last hour before fixation. After quantification of the signals from each cell, we trained a neural network to detect different cell cycle phases. DAPI intensity was used to distinguish 2N versus 4N DNA content, and EdU incorporation identified S‑phase cells. These experimentally annotated stages were then used to confirm the accuracy of the computational classifications. Not surprisingly, considering the measurements of DNA content, EdU incorporation, and main cell cycle regulators, the neural network could accurately identify cell cycle phases (Fig. 2A–C). This shows that data from cumulative microscopy can be used for neural-network-based predictions.

We next sought to test whether a neural network based on cell cycle and stress response markers could identify cells from different disorders. We therefore trained the network on data from primary fibroblasts from CHH and MUL patients and matched controls. Interestingly, although the data is based solely on cell cycle and stress response markers, 31 and 41% of CHH and MUL cells were correctly identified (Fig. 2D). Whereas identification of MUL cells was most efficient in the G1 phase, identification of CHH cells was most efficient in S and G2 phases (Fig. 2D). This indicates that at least a subset of CHH and MUL cells have stress and cell cycle characteristics that separate them from control cells and from each other.

As CHH cells, but not MUL cells, were mainly identified in S and G2 phases, we reasoned that these cells could exhibit some difference during DNA replication. We therefore perturbed DNA replication by adding a low level of the DNA-polymerase inhibitor Aphidicolin. The low level was chosen to perturb, but not completely block proliferation. After cumulative microscopy, we trained a neural network to score whether S-phase control fibroblasts were exposed to aphidicolin (Fig. 2E, left). We next used the network trained on control cells treated or not with aphidicolin to score CHH and MUL cells in the absence of aphidicolin. Interestingly, although CHH cells were not treated with aphidicolin, the neural network scored 26% of S phase cells as treated with aphidicolin. In contrast, control and MUL cells were not identified as treated with aphidicolin (Fig. 2E, right). This suggests that at least a subset of S-phase CHH cells, but not MUL cells, are sharing properties with control cells treated with Aphidicolin.

**Figure 3:**
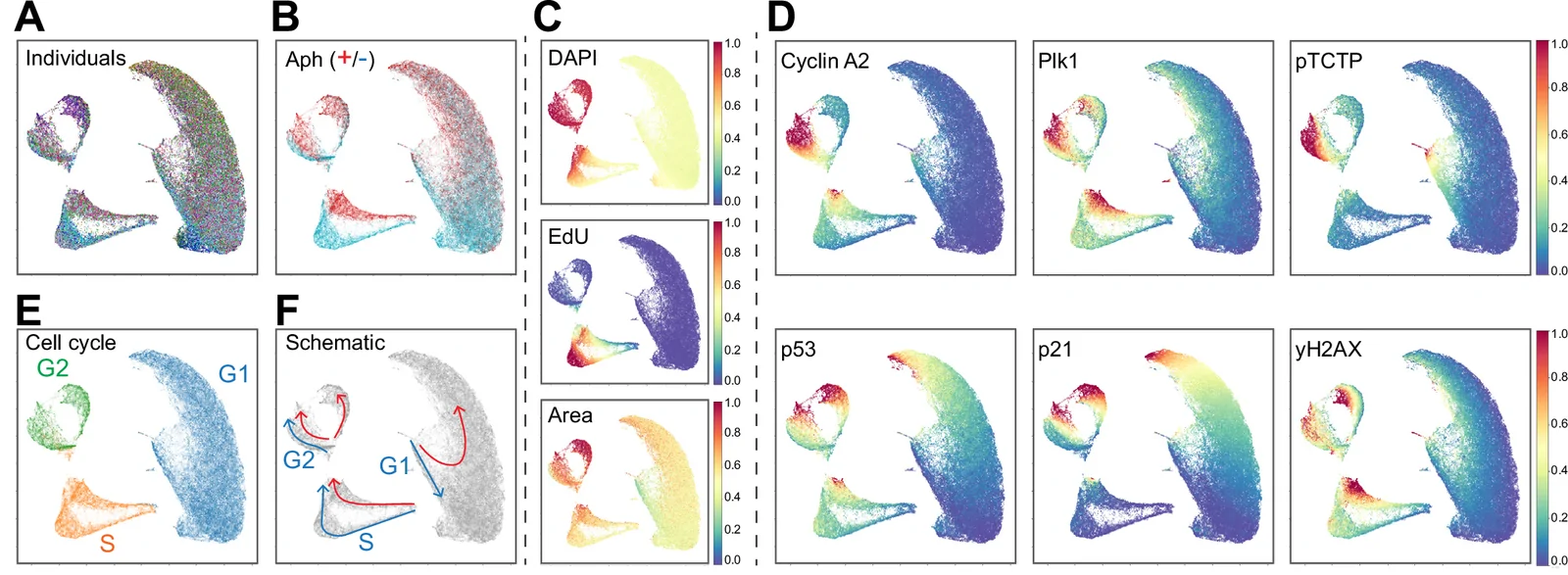
Assessing cell states after cumulative microscopy. (**A**) UMAP of cumulative microscopy data of in total 96,120 primary cells from 15 individuals, with or without 48 h exposure to aphidicolin. Colors indicate the 30 different conditions. Data are representative of two independent experiments with a total of four technical replicates. (**B**) UMAP from (**A**), indicating with or without exposure to aphidicolin. (**C**) UMAP from (**A**), indicating DAPI, EdU levels, and nuclear area. (**D**) UMAP from (**A**), indicating the antibodies used for cumulative microscopy. (**E**) UMAP from (**A**), indicating cell cycle stage based on DAPI and EdU data. (**F**) UMAP from (**A**), arrows indicate assessed main cell cycle trajectories. Blue indicates unperturbed cells, and red indicates after aphidicolin exposure. Source data are available online for this figure.

To visualize the cellular states, we performed a uniform manifold approximation and projection (UMAP) on all data combined and noted that fibroblasts from different individuals aligned in similar patterns (Fig. 3A). These patterns resembled distinct cellular states depending on cell cycle and stress responses. Importantly, by overlaying the individual antibody patterns, it was possible to identify states as replication stress, DNA damage checkpoints, and cell cycle exit (Fig. 3B–E). We identified the predominant trajectories of the unperturbed cell cycle in untreated cells (blue cells in Fig. 3B, blue arrows in Fig. 3F), starting with 2N DNA content cells that grew larger in G1 phase. Entering the S phase, these cells incorporated EdU and showed increasing DNA content, as well as low but increasing expression of Cyclin A2 and Plk1. After the S phase, a main trajectory characterized by 4 N DNA content, no EdU incorporation, and increasing levels of Cyclin A2 and Plk1, as well as phosphorylation of the Plk1 target TCTP, was visible. Similarly, we identified the predominant trajectories in cells treated with a low level of aphidicolin (red arrows in Fig. 3F). As expected, we find that S-phase cells gain a separate trajectory after Aphidicolin addition, characterized by reduced EdU incorporation and increased γH2AX levels, consistent with cells experiencing impeded DNA replication. In addition, we note that both G1 cells and G2 cells gained separate trajectories, characterized among others by expression of p53 and p21.

**Figure 4:**
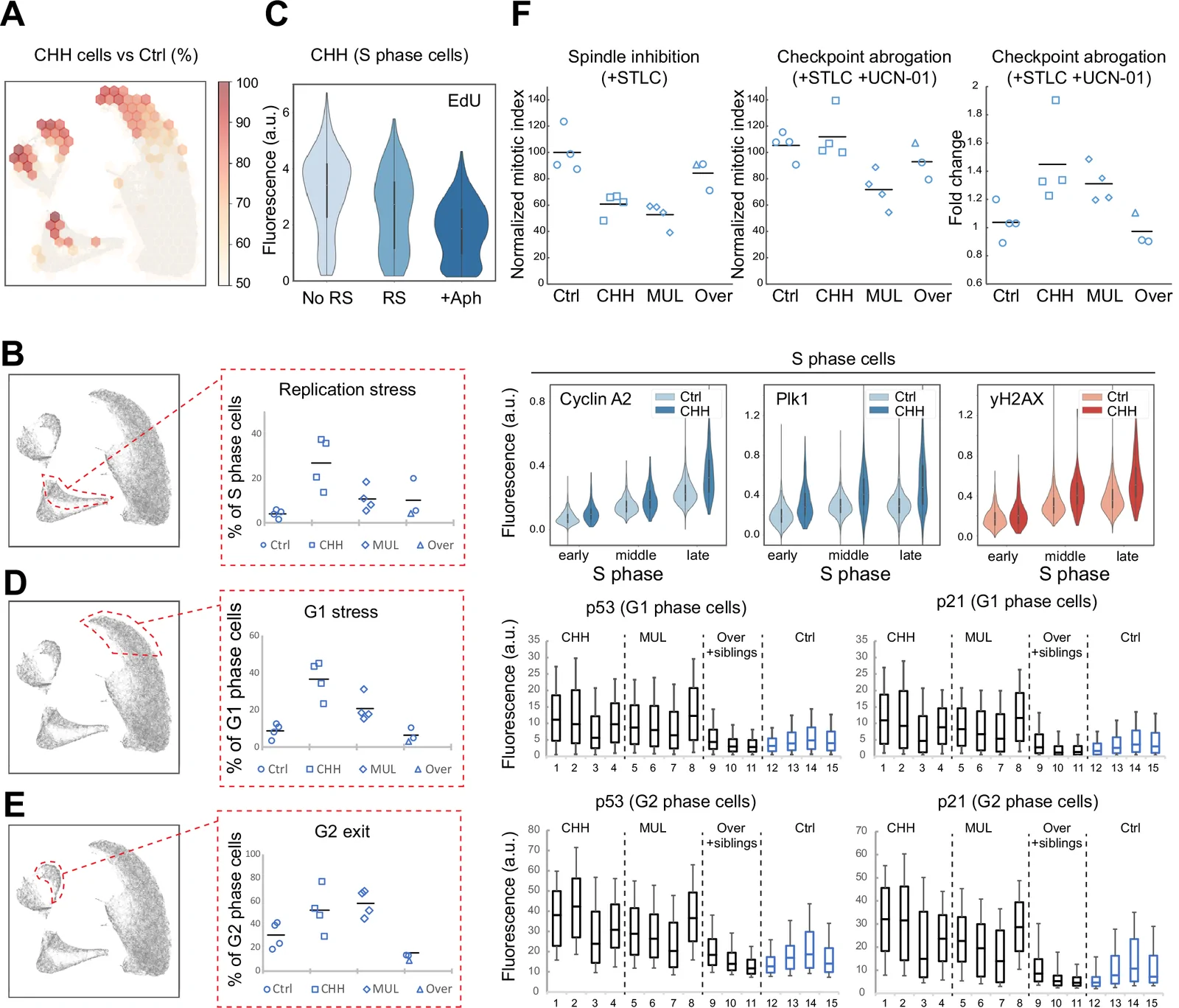
A subset of CHH cells shows replication stress and cell cycle exit in both G1 and G2 phases. Data in A-E is based on the UMAP in Fig. 3, which is representative of 2 independent experiments with a total of four technical replicates. (**A**) Accumulation of CHH cells. The graph shows the percentage of CHH cells versus Ctrl cells. The number of cells was normalized and binned into hexagons through the UMAP. (**B**) Left, gate in UMAP to depict replication stress. Right, distribution of indicated measurements in S-phase cells. Violin plots show data density with internal markers for the median and interquartile range. (**C**) EdU incorporation in S phase for different fractions of CHH cells: No RS – cells colocalized with untreated Ctrl replicating cells, RS – cells colocalized with cells treated with aphidicolin, and fraction ( + Aph) – cells treated with aphidicolin. Violin plots show data density with internal markers for the median and interquartile range. (**D**) Left, gate in UMAP to depict G1 stress. Right, distribution of indicated measurements in G1 phase cells. Box plots represent the median, 25th and 75th percentiles, with whiskers corresponding to the 10th and 90th percentiles. See Table EV2 for *P* values based on *t* test. (**E**) Left, gate in UMAP to depict cell cycle exit from G2 phase. Right, distribution of indicated measurements in G2 phase cells. Box plots represent the median, 25th and 75th percentiles, with whiskers corresponding to the 10th and 90th percentiles. See Table EV2 for *P* values based on *t* test. (**F**) Percentage of mitotic fibroblasts quantified by FACS, normalized to the mean value of control cells for each condition. Each dot represents the mean of three independent experiments. Left panel: percentage of mitotic cells following spindle inhibition with STLC. Middle panel: percentage of mitotic cells following combined treatment with STLC and UCN‑01. Right panel: fold change relative to control between STLC and combined STLC and UCN‑01 treatment. Source data are available online for this figure.

**Figure EV4:**
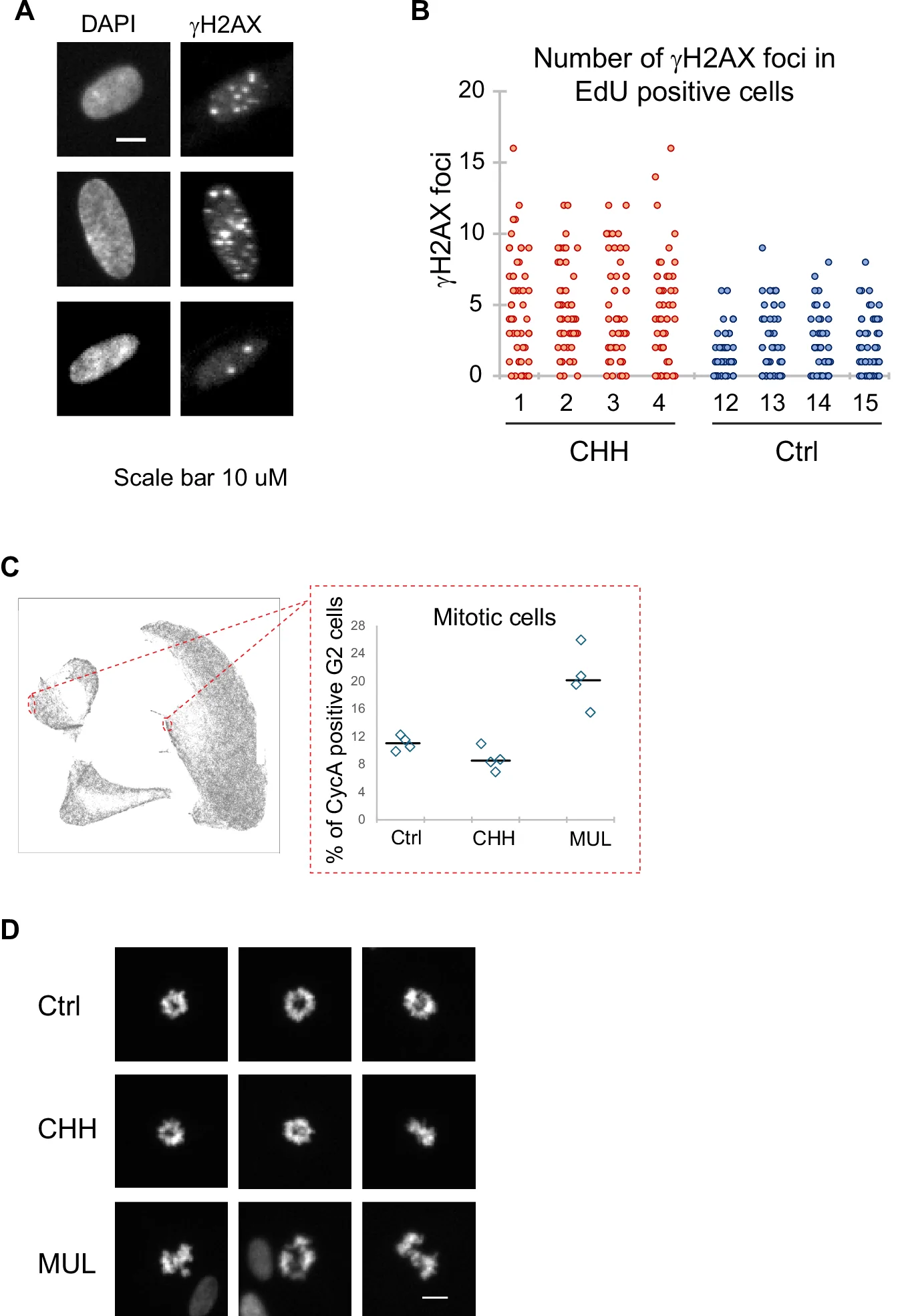
Quantification of γH2AX foci and mitotic cells. (**A**) Representative images for DAPI (left column) and γH2AX (right column) staining. Scale bar 10 μm. (**B**) Quantification of γH2AX foci in EdU-positive CHH (red) vs corresponding Ctrl (blue) cells. (**C**) Gate in UMAP to depict mitotic cells (left). Percentage of mitotic cells for Ctrl, CHH, and MUL in relation to all cells (middle) and Cyclin A positive G2 cells (right). (**D**) Representative images for DAPI staining in mitosis for Ctrl, CHH, and MUL cells. Scale bar 10 μm.

We next gated the distributions of cells from each individual in the UMAP. We noted that cells from 4 individuals diagnosed with CHH showed a different pattern compared to control cells (Fig. 4A). First, an increased fraction of the untreated CHH cells colocalized with replicating cells treated with the DNA polymerase inhibitor aphidicolin (Figs. 3B and 4B). Similar to cells treated with aphidicolin, these CHH cells showed an accumulation of γH2AX, indicating DNA damage, and increased Cyclin A2 and Plk1 levels compared to their position in S phase, indicating that the cell cycle engine had proceeded further than the replication of DNA (Fig. 4B; Appendix Fig. S4) (Lemmens et al, 2018). Similarly, CHH cells in S phase showed a reduction of EdU incorporation, although to a lesser extent compared to cells treated with aphidicolin, which directly inhibits DNA polymerase (Fig. 4C; Appendix Fig. S4). To test this finding independently of the UMAP, we counted γH2AX foci in EdU-positive CHH and control cells. We find that a subset of S-phase fibroblasts from 4 CHH patients contains an increased number of γH2AX foci (Fig. EV4A,B). Thus, a subset of CHH cells experiences replication stress.

Replication stress can lead to p53- and p21-dependent cell cycle exit in the following G2 phase, characterized by a loss of mitotic inducers, such as Cyclin A2 or Cyclin B1 (Hornsveld et al, 2021). Alternatively, if the cells pass through mitosis, replication stress can lead to a p53 and p21-dependent cell cycle block or delay in the following G1 phase (Arora et al, 2017). Both trajectories depend on p53, but whereas arrest from the G2 phase is linked to the presence of damaged DNA as indicated by γH2AX, cells blocking or delaying in the following G1 phase do not necessarily contain γH2AX (Stallaert et al, 2022b, 2022a). We found that CHH cells accumulated in the corresponding regions in the UMAP and that both G1 and G2 cells contained increased levels of p53 and p21 (Fig. 4D,E). Similar to CHH cells, we found that cells from 4 individuals diagnosed with MUL contained upregulated p53 and p21 and accumulated in the regions for G1 cell cycle stress and G2 cell cycle exit (Fig. 4D,E). However, in contrast to CHH, MUL cells did not show as clear an increased colocalization with aphidicolin-treated cells (Fig. 4B). This difference possibly reflects underlying mutations for the conditions, as MUL cells contain mutations in *TRIM37* that is involved in ensuring bipolar spindle assembly in mitosis (Balestra et al, 2021; Avela et al, 2000; Bellaart et al, 2025; Yeow et al, 2025). Consistent with this possibility, we detect an enrichment of MUL cells in mitosis in relation to the amount of cycling G2 cells (Fig. EV4C,D). Cells from an individual with an unspecified overgrowth syndrome did not accumulate in regions for cell cycle exit or arrest, suggesting faithful execution of the cell cycle. These results indicate that CHH and MUL cells both show a stress-induced block to proliferation, but by different mechanisms.

**Figure EV5:**
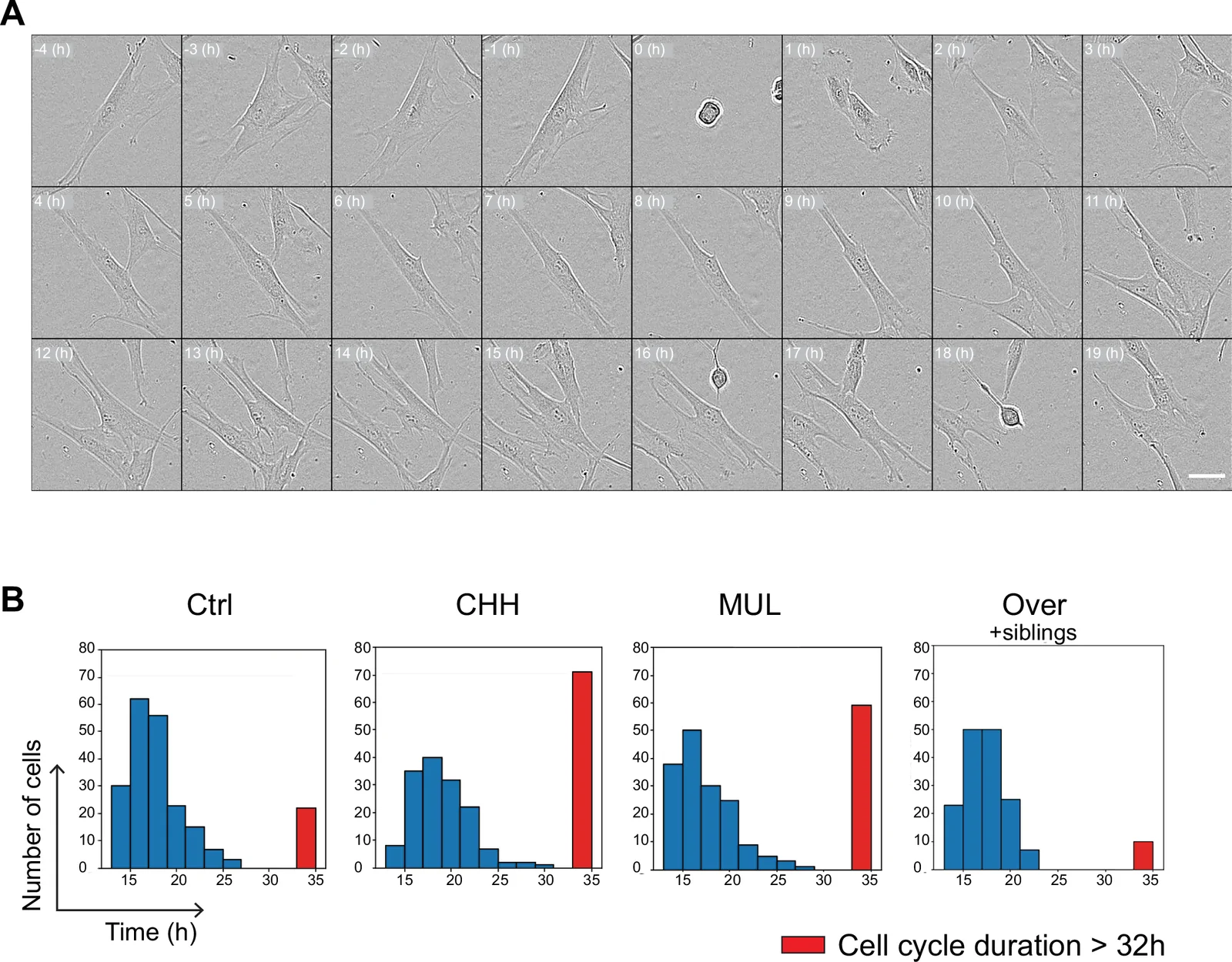
Time-lapse imaging of patient-derived fibroblasts. (**A**) Example of time-lapse images following a single cell. Scale bar 40 μm. (**B**) Quantification of the time between mitosis for the indicated cells. Cells for which the duration between mitoses was longer than 32 h, or two mitoses were not observed, are shown in red. Please note that “Over” here refers to both cells derived from the patient with overgrowth syndrome and the patient’s siblings. Data shown are representative of two independent experiments, each performed with technical replicates.

To independently test the results obtained by cumulative microscopy, we first monitored cells using time-lapse microscopy. Whereas both MUL and CHH cells showed an increased number of cells that are not dividing, only CHH cells showed an increased cell cycle duration (Appendix Fig. S2D; Fig. EV5). Next, we monitored fibroblasts by flow cytometry. First, we assessed cell cycle distribution and found that similar trends are visible using both immunofluorescence and flow cytometry (Appendix Fig. S5). To address the presence of a G2 checkpoint, we next used flow cytometry to assess the fraction of cells that accumulated in mitosis during a 4 h block of mitotic progression using STLC. We find that fewer CHH and MUL cells accumulate in mitosis after STLC treatment only, but that a larger fraction of cells accumulates in mitosis after co-treatment with the CHK1 and p38 inhibitor UCN-01 (Fig. 4F). We note that these data are consistent with a model in which a subset of CHH cells experience spontaneous replication stress followed by checkpoint activation in G2 phase (Vakkilainen et al, 2019) (Appendix Fig. S2D; Fig. EV5).

Replication stress has emerged as a key driver of genome instability in cancer, and a common feature of multiple oncogenes (Halazonetis et al, 2008; Gaillard et al, 2015). More recently, treatment options that target replication stress have been considered (Cybulla and Vindigni, 2023). We note that replication stress potentially could underlie both proliferative defects and increased cancer risk in CHH patients.

CHH patients carry biallelic pathogenic variants in the *RMRP* gene, but whether and how *RMRP* contributes to replication stress requires further investigation. However, we note that an inverse correlation exists between *RMRP* expression and a replication stress score (see methods) in established cell lines (Appendix Fig. S6).

We conclude that cumulative microscopy is a straightforward method to multiplex immunofluorescence that does not require elution or bleaching steps. The method is quantitative, can be performed using microscopes that are commonly available, does not require antibody conjugation steps, and can be used to support multi-labeling-based identification of cellular states. Importantly, the method is tested here for acquisition using air objectives, and has not been tested for high-resolution or super-resolution imaging. As cumulative microscopy is based on immunofluorescence, any artifact or variability introduced by immunofluorescence will still be retained. In addition, as antibodies are retained, cumulative microscopy precludes the use of primary antibodies that would sterically hinder each other from binding to an epitope. Although tested for antibody-based labeling here, cumulative microscopy can in theory be expanded to other forms of microscopy that involve quantification of a fluorescent signal.

## Methods

**Table:** Reagents and tools table

Reagent	Source	Identifier
Ascorbic acid	Sigma-Aldrich	A4403
Aphidicolin	Sigma-Aldrich	A4487
Azid Fluorophore (Alexa 647)	Invitrogen	A10277
BSA	Sigma-Aldrich	A4503
CuSO_4_	Sigma-Aldrich	451657
DAPI	ThermoFisher	62248
DMSO	Sigma-Aldrich	D4540
EdU (5-ethynyl-2’-deoxyuridine)	ThermoFisher	E10187
anti-Mouse conjugated to Alexa Fluor™ 568	Invitrogen	A11004
anti-Rabbit conjugated to Alexa Fluor™ 488	Invitrogen	A11034
anti-Rabbit conjugated to Alexa Fluor™ 647	Invitrogen	A21245
anti-Rabbit conjugated to Alexa Fluor™ 647	Invitrogen	A31573
anti-Goat conjugated to Alexa Fluor™ 488	Invitrogen	A11055
anti-phospho-Histone H3 (Ser10) (D2C8)	Cell Signaling	3377
Methanol	Sigma-Aldrich	32213 N
Goat anti-Cdk2	Santa Cruz	sc-163-G
Goat anti-Cdk4	Santa Cruz	sc-260-G
Goat anti-Lamin	Santa Cruz	sc-6217
Hoechst 33342	ThermoFisher	62249
Mouse anti-p53	Santa Cruz	sc-126
Mouse anti-Cyclin B1	Sigma-Aldrich	WH0000891M1
Mouse anti-pRb	BD Pharmingen	558389
Mouse anti-Plk1	Santa Cruz	sc-17783
Mouse anti γH2AX	Millipore	05-636
PBS	Gibco	14190-144
Rabbit anti-Aurora A	Cell Signaling	4718
Rabbit anti-ATAD2	Sigma-Aldrich	HPA029424
Rabbit anti-Cyclin A2	Atlas antibodies	HPA020626
Rabbit anti-p21	Cell Signaling	2947
Rabbit anti-Phospho-TCTP	Cell Signaling	5251
STLC	Tocris	2191
TBS (Tris-buffered saline pH 7.6)	ThermoFisher	J62662
TBS-T (TBS containing 0.1% Tween-20)	Sigma-Aldrich	P1379
Tris (pH 6.8)	Sigma-Aldrich	T1503
UCN01	Sigma-Aldrich	U6508

### Primary fibroblasts

The collection of patient and control skin biopsies for research purposes was approved by the Institutional Research Ethics Committee of the Helsinki University Hospital, Finland (HUS/836/2018, HUS/1190/2018). All the participants gave informed consent.

Forearm skin biopsies were harvested from the mutation-positive subjects (four with CHH and four with MUL) and from four age- and sex- matched controls. Further, skin biopsies were harvested from a subject with an unspecified overgrowth syndrome, as well as from two siblings without overgrowth. Harvesting of skin biopsies and isolation of fibroblasts were performed as described in (Pekkinen et al, 2019).

### Cell culture

RPE1-hTERT (RRID:CVCL_4388) and RPE1 - CyclinA2-YFP cells (Silva Cascales et al, 2021) were cultured in DMEM-F12 medium plus GlutaMAX (Invitrogen) supplemented with 10% FBS (HyClone) and 1% Penicillin–Streptomycin (HyClone). Cells were not recently authenticated. Primary human fibroblasts were cultured in DMEM medium plus GlutaMAX (Invitrogen) supplemented with 10% FBS (HyClone) and 1% Penicillin–Streptomycin (HyClone). Cells were maintained at 37 °C and 5% CO_2_.

Before experiments, cells were seeded in 96-well imaging plates (Falcon™ 96-Well, Cell Culture-Treated, Flat-Bottom Microplate) for overnight.

For fixed-cell experiments, cells were fixed in 3.7% formaldehyde for 5 min and permeabilized in ice‑cold methanol for 2 min. Samples were blocked for 1 h at room temperature in TBS‑T (0.1% Tween‑20) containing 2% BSA, followed by 1 h incubation with primary antibodies. After three washes in TBS‑T, cells were incubated with secondary antibodies and DAPI for 1 h at room temperature, washed again, and stored in TBS or PBS.

EdU incorporation was detected by Click chemistry using a reaction mix containing 100 mM Tris (pH 6.8), 1 mM CuSO₄, 10 mM ascorbic acid, and 3 µM azide‑fluorophore (Invitrogen) for 30 min at room temperature.

### Immunofluorescence and microscopy

Immunofluorescence was performed as in (Akopyan et al, 2016). Several systems, including an ImageXpress microscopy system equipped with a CoolSNAP HQ camera (Molecular Devices/LLC, Sunnyvale, CA, USA) and a Nikon ×20 Plan Fluor ELWD NA 0.45 objective, an ImageXpress HCS high content imaging system with ×20 LWD Air objective, and Nikon CrEST X-Light V3 with 20× PLAN APO NA 0.80 objective, were used to acquire fluorescent images.

### Live-cell Imaging

Primary fibroblasts were followed on a Sartorius IncuCyte S3 Long-term imaging inside a cell culture incubator system using a 20× NA 0.45 air objective for 60 h. Images were acquired every 1 h.

RPE-CyclinA2-YFP cells were followed on a Leica DMI6000 Imaging System using a 20× NA 0.40 air objective. Images were acquired every 30 min. The medium of the cells was changed 24 h prior to imaging to CO_2_-independent medium Leibovitz L-15 (ThermoFisher) supplemented with 6% or 10% heat-inactivated FBS and 1% Penicillin–Streptomycin (HyClone).

### Image processing

Images were processed using ImageJ (http://rsb.info.nih.gov/ij/) or Python. Background subtraction was performed using background functions as in (Akopyan et al, 2016). Nuclei were segmented based on DAPI signal, and integrated intensities of the nuclei were quantified. Please note that for comparison across image cycles, segmentation needs to be performed on a structure that remains similar across cycles—such as nuclei in a separate channel. Protein expression levels were assessed by measuring the integrated intensities within the corresponding nuclear masks.

### Image registration and field‑of‑view alignment

For cumulative microscopy images were aligned using a custom Python script developed to correct positional drift across acquisition cycles. Image data were organized in subdirectories corresponding to repeated imaging cycles (c1, c2, c3, …), each containing TIFF images with identical filenames. For each sample, one reference cycle (c1) was used as the alignment anchor.

To estimate the translational offset between cycles, the script automatically selected images containing the substring “Side 1_ Wavelength1”, which served as the fiducial channel. Each pair of corresponding images across cycles was converted to 32‑bit grayscale, and the translational shift relative to the reference image was computed using phase correlation in the Fourier domain, a robust and widely used method for detecting spatial translations between images. The resulting per‑cycle shifts were averaged (rounded to integer pixel values) to obtain a stable displacement estimate for each cycle.

After determining the translational offsets for all cycles belonging to a sample, the script calculated the largest common overlapping region shared by all images. All TIFF files in each cycle directory were then cropped to this common field of view after compensating for the estimated shift. This produced a set of spatially aligned, identically sized images for downstream analysis.

Time estimation from fixed cells was performed by ordering cells based on increasing levels of Cyclin A2 and DAPI as in (Akopyan et al, 2014).

### Cumulative microscopy

A detailed description of cumulative microscopy can be found in the Appendix.

Briefly, a scaling function was calculated using linear regression of the signal from one area for two consecutive cycles, but with primary antibodies omitted in the second cycle. Except for Fig. 1B, Appendix Fig. S1, and Fig. EV2A, in which scaling functions were calculated from pixel values, scaling functions were based on measurements of the integrated intensity of segmented nuclei. The scaling function was used to scale the signal of either the image (pixel values) or the measurements from one imaging cycle, which was then subtracted from the same image or measurements of the next imaging cycle. Calculations were performed using Python. Code to perform cumulative microscopy is available at https://github.com/KAkopyan/Cumulative-Microscopy-Python.

### Dimensionality reduction algorithm and simulations

Uniform Manifold Approximation and Projection (UMAP) and visualization were performed in Python using *sklearn.datasets, sklearn.model_selection, sklearn.preprocessing, matplotlib.pyplot, numpy, seaborn, pandas*, and *colorcet*.

The dataset used for UMAP construction contained quantitative single‑cell measurements for nuclear area, DAPI intensity, EdU incorporation, γH2AX, pTCTP, Plk1, Cyclin A, p53, and p21 expression. In total, approximately 96,000 fibroblasts were profiled, derived from 15 individuals, including CHH and MUL patients as well as healthy controls. The dataset also included cells treated with Aphidicolin to induce replication stress. All data for the UMAP were collected from cells growing in the same 96-well plate.

For each individual sample (well), intensity normalization was performed to account for variability in staining intensity and imaging conditions across wells. Specifically, a per-sample scaling factor was computed as follows:

The DAPI intensity distribution of G1-phase cells was used to estimate a normalization factor. The dominant low-intensity bin (representing baseline DNA content) was identified from a binned histogram, and the midpoint of this bin was used as the correction value. All DAPI values within each sample were then normalized by dividing by the corresponding correction factor, resulting in comparable relative DNA content across samples.

Following sample-wise normalization and concatenation, the combined dataset was globally standardized using z-score normalization (zero mean and unit variance) across all cells and features (StandardScaler, scikit-learn).

Data integration across multiple wells was performed by concatenating all normalized single-cell measurements into a single dataset prior to scaling and embedding. No explicit batch correction algorithm was applied; instead, normalization to per-sample DAPI intensity and subsequent global standardization were used to mitigate inter-sample variability.

Cell‑cycle phases and progression trajectories were inferred based on established marker profiles, integrating DNA content (DAPI), DNA synthesis activity (EdU), and cell cycle markers (e.g., Plk1 and Cyclin A). Mitotic cells were manually identified based on DAPI staining to mark the regions corresponding to mitotic cells.

Simulations for the relation of R-square value to normalized standard deviation were performed in Python using: *matplotlib.pyplot, numpy, scipy.stats, seaborn, pandas, pathlib*, and *math*.

### Neural network architecture and training procedure

A feedforward neural network was implemented using PyTorch (version 2.5.1) to classify cell types, cell phases, or conditions based on input data. The model architecture consists of an input layer with 9 neurons, followed by three fully connected hidden layers with 8, 9, and 9 neurons, respectively. Each hidden layer uses the Rectified Linear Unit (ReLU) activation function to introduce non-linearity. The final output layer comprises 2 neurons corresponding to the binary classification task (e.g., Aphidicolin-treated vs. not treated cells) or 3 neurons in the case of classifying cell types or cell phases.

Prior to training, the dataset of 96 000 cells was split into training and test sets using an 80:20 ratio with a fixed random seed for reproducibility. Features in the dataset consist of the fluorescence intensity from the measured antibodies, DAPI, and EdU (Appendix Fig. S2D), as well as nuclear area. Input features were standardized using the StandardScaler from the scikit-learn library to ensure consistent scaling across all features. The model was trained using the Adam optimizer with a learning rate of 0.01. The loss function used was cross-entropy loss, suitable for multi-class classification tasks. Training was conducted over 600 epochs, and the model’s performance was monitored by tracking the training loss at regular intervals.

The model was trained and evaluated on separate datasets to assess its generalizability across different experimental conditions.

### Replication stress score

The replication stress (Repstress) score was calculated by summing the expression levels of 17 signature genes, each weighted according to previously published coefficients (Takahashi et al, 2022). Gene expression values were Z score–normalized across all genes within each cell line. The resulting Repstress scores were then Z score–normalized across all samples to enable cross-sample comparison.

RNA-seq data for 675 commonly used human cancer cell lines (RNA-seq of 675 commonly used human cancer cell lines) were downloaded from the EMBL-EBI Expression Atlas (https://www.ebi.ac.uk/gxa/experiments/E-MTAB-2706/Downloads). Only cell lines with non-zero expression values for RMRP, TRIM37, and all 17 signature genes were included in the analysis, resulting in a final dataset of 180 cell lines spanning 49 cancer types.

### Signal-intensity distribution analysis

Signal-intensity distributions from Cumulative IF and Standard IF were compared by analyzing single‑cell intensity values for each condition. Intensities were normalized by dividing each dataset by the mean intensity. Normalized distributions were then statistically compared using an unpaired two‑sample *t* test for mean differences and the Kolmogorov–Smirnov (KS) test for distribution differences. For visualization, kernel density-based histograms (Seaborn histplot, with KDE enabled and density normalization) were used to display the normalized intensity distributions.

### Flow cytometry

Flow cytometric analysis was performed using a BD FACSCanto II flow cytometer. Mitotic cells were defined as cells with high pH3 levels and 4 N DNA content, whereas S-phase cells were identified by EdU incorporation. Cells were incubated with EdU for 1 h before harvest. After collection, all cells were fixed in 70% ethanol. EdU incorporation was analyzed as described above. DNA content was determined by Hoechst 33342 staining. Mitotic cells were stained with rabbit anti-phospho-Histone H3 (Ser10) (D2C8) antibody (Cell Signaling Technology, #3377, 1:200). For assessment of G2-checkpoint function, cells were treated with S-trityl-L-cysteine (STLC, selective inhibitor of mitotic kinesin Eg5, 10 μM, Tocris Bioscience, Cat. No. 2191) for 4 h, either alone or in combination with UCN-01 (CHK1 and p38 inhibitor, 300 nM, Sigma-Aldrich, Cat.No. U6508). Data were analyzed using FlowJo software, and cell doublets were excluded from all analyses. The gating scheme is shown in Appendix Fig. S7.

## Supplementary information

Table EV1

Table EV2

Appendix

Peer Review File

Source data Fig. 1

Source data Fig. 2

Source data Fig. 3

Source data Fig. 4

Expanded View Figures

## Data Availability

Raw imaging data from cumulative microscopy of patient fibroblasts is available at https://www.ebi.ac.uk/biostudies/bioimages/studies/S-BIAD3443 (https://doi.org/10.6019/S-BIAD3443). Code to perform cumulative microscopy is available at https://github.com/KAkopyan/Cumulative-Microscopy-Python. The source data of this paper are collected in the following database record: biostudies:S-SCDT-10_1038-S44319-026-00859-5.
