# Supplementary material for: Cumulative microscopy reveals cellular states in fibroblasts from patients with genetic disorders
Source: EMBO Rep. 2026 Jul 30;27(17):5022–38. doi: 10.1038/s44319-026-00859-5 (PMC13554152; doi:10.1038/s44319-026-00859-5)
Supplement: Supplementary file 1 [file 44319_2026_859_MOESM3_ESM.pdf]

Appendix

Cumulative microscopy reveals cellular states in fibroblasts from patients with genetic disorders

Karen Akopyan[1], Zhiyu Hao[1], Susann Karlberg[2,3,4], Svetlana Vakkilainen[2,3,4], Fulya Taylan[5], Minna Pekkinen[2,3,4], Outi Mäkitie[2,3,4,5], Arne Lindqvist[1]*

Table of contents

Appendix methods, cumulative microscopy

Appendix methods, step-by-step workflow for cumulative microscopy

Appendix figure S1

Appendix figure S2

Appendix figure S3

Appendix figure S4

Appendix figure S5

Appendix figure S6

Appendix figure S7

Appendix methods, cumulative microscopy

Setup

Cumulative microscopy is based on performing multiple imaging cycles, without removing the signal between individual cycles. Rather, each cycle contributes to more staining, and the staining of an image therefore grows in a cumulative fashion. To access a particular staining, the preceding image or quantification is subtracted. This subtraction needs to be calibrated, as bleaching of fluorophores, background staining, and possibly changed image exposure and illumination settings may affect the contribution of how much of the signal from one imaging cycle is retained in the next imaging cycle. Further, additional binding of a secondary antibody to primary antibodies from preceding cycles may affect the signal that should be subtracted. Therefore, for each imaging cycle, a unique staining is required in which it is possible to assess how to calibrate the subtraction for that very imaging cycle. This unique staining to calibrate the subtraction is obtained by re-staining a sample with only the secondary antibody, as detailed below.

For quantitative immunofluorescence, we use the same secondary antibodies for all stainings in all wells. For each imaging cycle, the primary antibodies are omitted from one well, although primary antibodies have been added and imaging performed on that well in preceding cycles. The total number of starting wells therefore scales with the number of imaging cycles (Figure EV1A). The well in which no primary antibody is added for an imaging cycle is compared to the same well in the previous imaging cycle. By performing a linear regression on measurements from this well, a scaling function can be assessed on how much bleaching, re-staining with a secondary antibody, and possibly changed exposure and illumination conditions have affected the signal. This scaling function is used to modify the image or measurements used for subtraction (Figure EV1B).

Estimating a scaling function for subtraction

The scaling function for two consecutive imaging cycles can be assessed by comparing the fluorescence intensity for each pixel value, or by comparing the measurements of segmented structures in the image, such as nuclei. We find that comparing segmented structures is practically more straightforward, as it is much less sensitive to small shifts in the image position, thus allowing processing without image registration. Further, although we find that a linear correlation can be used to approximate both measurements of pixel values and segmented structures, at least the theoretical possibility exists that background pixel values and pixel values from areas occupied by cells would require two different linear functions. By using measurements of segmented structures rather than all pixels, the scaling function can be assessed only on structures that are of interest for further analysis (Appendix Figure S1).

To evaluate the robustness of the calibration procedure, we tested the scaling approach across more than ten antibody targets and four fluorophores commonly used in our imaging pipeline. These targets represented a range of staining patterns, including stress-response markers, allowing us to assess the stability of the calibration function across biologically diverse signals. For each antibody–fluorophore combination, we quantified both absolute intensity levels and relative signal changes across imaging cycles to ensure that the scaling procedure generalized reliably to different staining conditions.

Antibody-specific variability, including differences in background intensity, signal-to-noise ratio, and staining specificity, was addressed by incorporating internal controls within each imaging round (Figure EV2). Background levels were estimated from cell-free regions adjacent to the segmented nuclei, enabling cycle-specific correction of non-specific fluorescence. In addition, antibodies with known variability profiles were evaluated through replicate staining across multiple samples, allowing us to distinguish biological differences from antibody-dependent fluctuations.

Calibration accuracy was assessed primarily on a per-cell (segmented structure) basis, consistent with our overall analytical pipeline, which relies on cellular measurements rather than pixel-level information (Except for Figure 1B, Appendix Figure S1, and Figure EV2A). While pixel-wise calibration is possible, we found that cell-level evaluation provides a more stable and interpretable basis for scaling, particularly because segmentation mitigates the impact of minor image shifts and local heterogeneities.

The calibration procedure is designed to correct all global, cycle-dependent fluctuations in signal intensity, including changes in illumination, exposure settings, and fluorophore decay between cycles. However, the method does not correct parameters that vary at the subcellular level within individual cells, such as spatially restricted antibody accessibility.

Retrieving a particular staining

The scaling function is used to modify the image used for subtraction. The subtraction can be performed directly on all pixel values to retrieve a physical image, as is shown in Figure 1B. However, similar to when assessing the scaling function, even minimal shifts in image position may affect the placement, thus risking introducing errors. Instead, we directly subtract the measurements of segmented structures, without creating an image, thus minimizing errors due to spatial shifts between imaging cycles (Figure 1C, Appendix Figure S1).

Subtraction variability

We reasoned that technical variability of cumulative microscopy could be estimated from the accuracy of the linear regression used to create a scaling function. We therefore plotted the distribution of differences between pairwise measurements from two consecutive imaging cycles, in which no primary antibody was added for the second imaging cycle (Figure EV3A, left). After linear regression to obtain a scaling function and correction by that scaling function, the data could be approximated by a normal distribution (Figure EV3A, middle). To allow comparison between stainings, we normalized the data to the fluorescence signal level and noted that the variability was low compared to the measured signal (Figure EV3A, right). Indeed, the technical variability was low compared to cell-cell variability of measured Cyclin A2 levels (Figure 1D, circle size indicates technical variability).

A common metric to describe a fit after linear regression is the R-square value. We simulated how the R-square value of a normally distributed pair-wise difference after creation and correction by a scaling function related to the normalized standard deviation. As the R-square value falls under 0.99, the standard deviation exceeds 8% of the signal, indicating that the R-square value can function as a readily available estimate for the technical variability for cumulative microscopy (Figure EV3B). In this sense, the technical variability of each

individual cycle is straightforward to assess. Importantly, as there is always a comparison between two consecutive steps, errors do not accumulate.

In addition to direct technical variability, other error sources may affect accuracy of cumulative microscopy. Notably, the method depends on comparing different stainings, which is a potential source for variability. One way to control for variability between wells is to include technical duplicates. Using the scaling function from one well to correct the signal of a parallel well, we did not detect a marked difference in variability (Figure EV3A). However, we advise to include technical duplicates to control for this potential error source.

Another possible error source is that the presence of antibodies from previous imaging cycles would sterically hinder antibodies to find their targets. This is likely applicable only when using antibodies to targets that are in close proximity. Nonetheless, one way to control for such steric hindrance is to compare the trend of one antibody staining using cumulative microscopy to the trend using ordinary quantitative immunofluorescence. We estimated the accumulation of Cyclin A2, which has a characteristic pattern of accumulation through the cell cycle. We detect no difference in the pattern of Cyclin A2 accumulation when assessed by cumulative microscopy or by ordinary quantitative immunofluorescence (Appendix Figure S2). Similarly, these patterns of accumulation agreed with the accumulation of Cyclin A2-YFP measured in live cells (Figure 1D). Thus, the technical variability by cumulative microscopy can be small in comparison to biological variation. Further, the technical variability can be estimated from the data and potential error sources can be controlled for by technical duplicates and comparison to single stainings. As cumulative microscopy is based on immunofluorescence, any artifact or variability introduced by immunofluorescence will still be retained.

Appendix methods, step-by-step workflow for cumulative microscopy

Immunofluorescence staining

Cells were seeded in 96-well imaging plates (Falcon™ 96-well, cell culture-treated, flat-bottom) and allowed to adhere overnight prior to experimentation. For each condition, one more well than number of staining cycles is needed (see Figure EV1A).

(*Optional*) For EdU labeling, cells were incubated with 10 μM EdU for 1 h prior to fixation, where applicable.

Cells were fixed with 3.7% formaldehyde for 5 min at room temperature, followed by permeabilization in ice-cold methanol for 2 min. After permeabilization, samples were blocked for 1 h at room temperature in TBS-T (0.1% Tween-20) supplemented with 2% BSA.

Primary antibodies were diluted in blocking buffer and incubated with cells for 1 h at room temperature. Following primary antibody incubation, cells were washed three times in TBS-T. Secondary antibodies and DAPI were then applied for 1 h at room temperature, followed by three additional washes in TBS-T.

(*Optional*) For EdU labeling, incorporation was detected using Click chemistry with a reaction mixture containing 100 mM Tris (pH 6.8), 1 mM $CuSO_4$, 10 mM ascorbic acid, and 3 μM azide-fluorophore (Invitrogen) for 30 min at room temperature.

After staining, cells were maintained in TBS or PBS until imaging.

Note: The applied fixation and staining protocol represents a commonly used workflow for adherent cells and commercially available antibodies. Compatibility with alternative fixation methods (e.g., methanol- or acetone-based fixation) depends primarily on antibody specificity and cell-type-dependent epitope preservation, and therefore may require empirical optimization.

Sequential staining and imaging

Imaging was performed directly in the 96-well plates with cells maintained in TBS or PBS. After initial image acquisition, samples were subjected to subsequent rounds of immunostaining without antibody stripping.

For each additional staining cycle, the buffer was removed and cells were incubated with a new set of primary antibodies for 1 h at room temperature, followed by three washes in TBS-T, incubation with secondary antibodies for 1 h at room temperature, and a final set of three washes. Fresh TBS or PBS was added prior to imaging.

Note: Although it is difficult to define a strict time limit between cycles, plates with samples can be stored at 4°C for extended periods if necessary, provided that the volume of TBS/PBS is sufficient to prevent sample drying.

Note: The same wells were imaged across cycles by reusing predefined stage coordinates, ensuring consistent field acquisition between imaging rounds (see section: *Field-of-view relocation and reproducible imaging*).

Imaging

Fluorescence imaging was performed using a widefield fluorescence microscope equipped with an automated stage and a 20× air objective (PLAN). Image acquisition settings (e.g., exposure time, illumination intensity) were adjusted to ensure optimal signal detection while avoiding saturation. The same imaging conditions are used within each cycle, but can be changed between cycles.

The use of a 20× air objective provides a balance between spatial resolution and field of view, allowing efficient imaging of a large number of cells per condition.

Note: The method is compatible with other fluorescence imaging platforms, provided that sufficient signal-to-noise ratio and appropriate dynamic range are maintained.

Field-of-view relocation and reproducible imaging

Imaging was performed using a fluorescence microscope equipped with an automated motorized stage. Stage coordinates corresponding to each well and field position were saved during the first imaging cycle and reused in subsequent cycles to ensure reproducible imaging of identical locations.

Although fixation prevents biological movement, minor positional shifts may occur due to stage repositioning accuracy. To correct for such shifts, post-acquisition image registration was applied to align images from different cycles at the pixel level.

Note: Optimal imaging conditions were achieved using moderate cell densities (~50–70% confluency), which facilitate reliable segmentation and consistent identification of cell populations across cycles. The field of view is determined by the imaging system and camera but is not a limiting factor, provided that identical acquisition settings are maintained between cycles.

Note: This workflow does not require specialized hardware beyond a standard automated stage microscope, although higher stage precision improves initial alignment and reduces the extent of computational correction required.

Image processing and computational workflow

Raw fluorescence images were acquired in a multi-channel format (e.g., TIFF) across sequential staining cycles. The computational workflow consists of the following steps:

1. Image organization
   Images from different staining cycles and channels were systematically organized according to well position, field of view, and staining round. Please see description on https://github.com/KAkopyan/Cumulative-Microscopy-Python for naming of image files.
2. Image registration
   To correct for small positional shifts introduced during stage repositioning, images from subsequent cycles were aligned to the first imaging cycle using image-registration algorithms. This step ensures pixel-level correspondence between cycles. A script to perform image registration is present at https://github.com/KAkopyan/Cumulative-Microscopy-Python
3. Quality control
   Images were evaluated to ensure:
   - absence of saturation

- sufficient signal-to-noise ratio
- consistent exposure conditions across cycles
- correct alignment between cycles
  Images not meeting these criteria were excluded from downstream analysis.

4. Signal processing and integration
   Registered images were processed using a custom Python-based script (available at: https://github.com/KAkopyan/Cumulative-Microscopy-Python) to generate cumulative microscopy datasets for downstream analysis.

Note: Image processing can be performed using standard image analysis tools (e.g., ImageJ/Fiji, Python-based workflows, or equivalent), and does not require specialized computational infrastructure. Typical analyses can be performed on a standard workstation equipped with a multi-core CPU and ≥16 GB RAM, with processing times depending on dataset size.

Appendix Figure S1

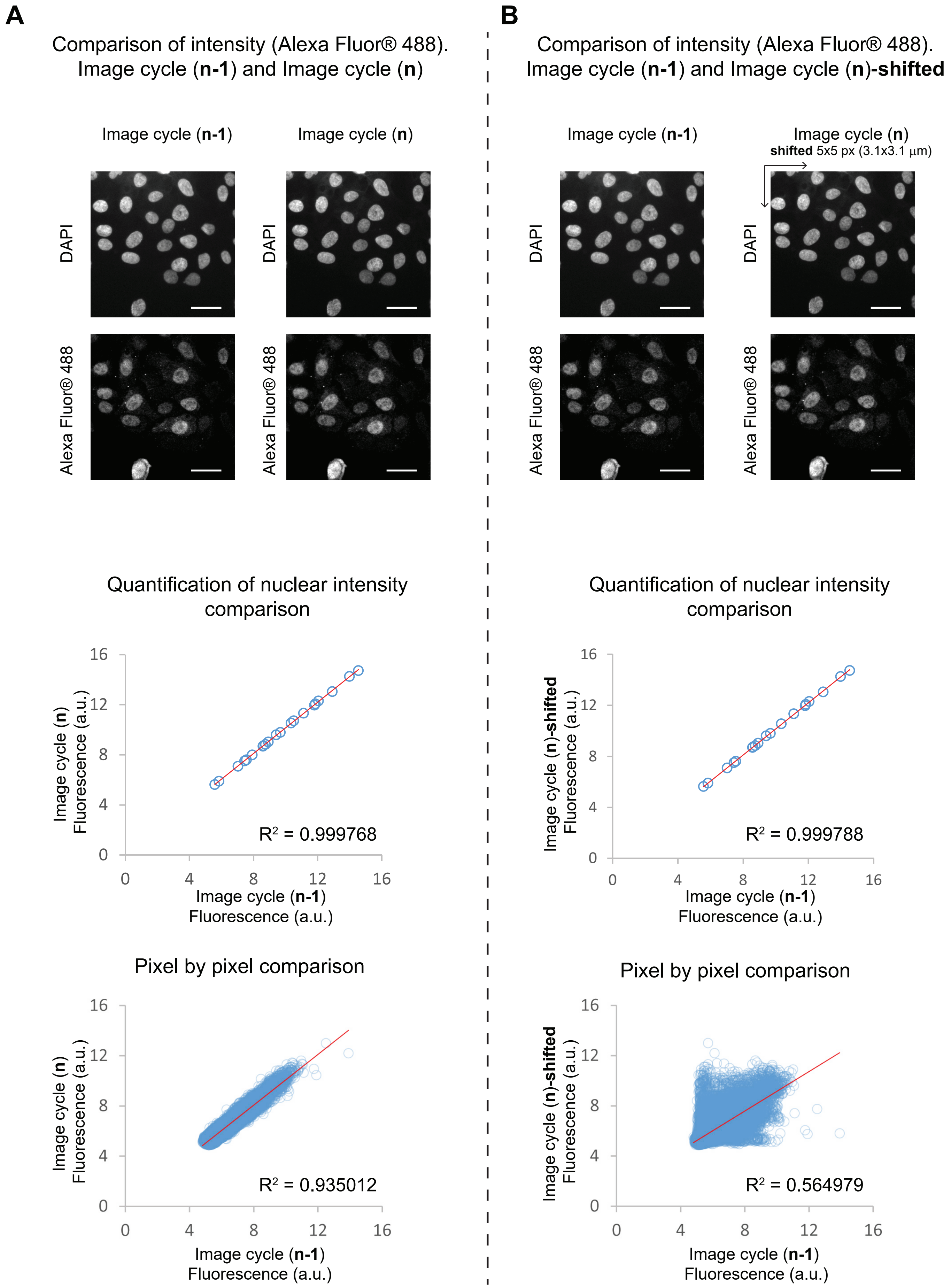

Appendix Figure S1. Comparison of estimating a scaling function based on pixel values or segmented structures.

Top, images show DAPI and secondary antibody staining of two successive imaging cycles, in which no new primary antibody is introduced. Middle, graphs show linear regression of integrated nuclear fluorescence from secondary antibody staining. The nuclei are segmented based on the DAPI staining. Bottom, graphs show linear regression of pixel values from secondary antibody staining.

A, the same area is imaged in two consecutive cycles.

B, using the same images as in A, the image for the second cycle is shifted by 5 pixels in both x and y directions.

Scale bar 40 μm.

Appendix Figure S2

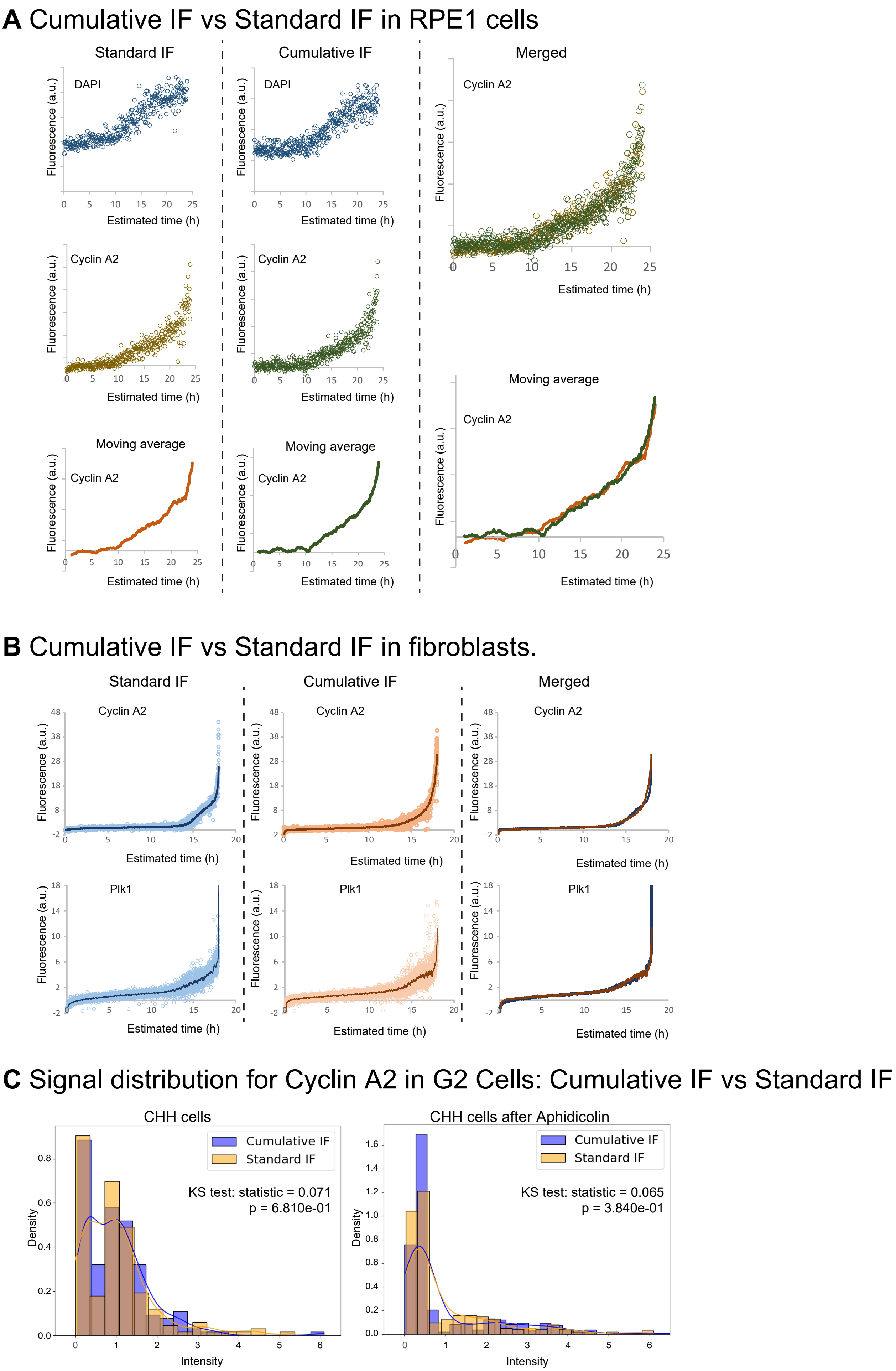

Appendix Figure S2

D Experimental setup

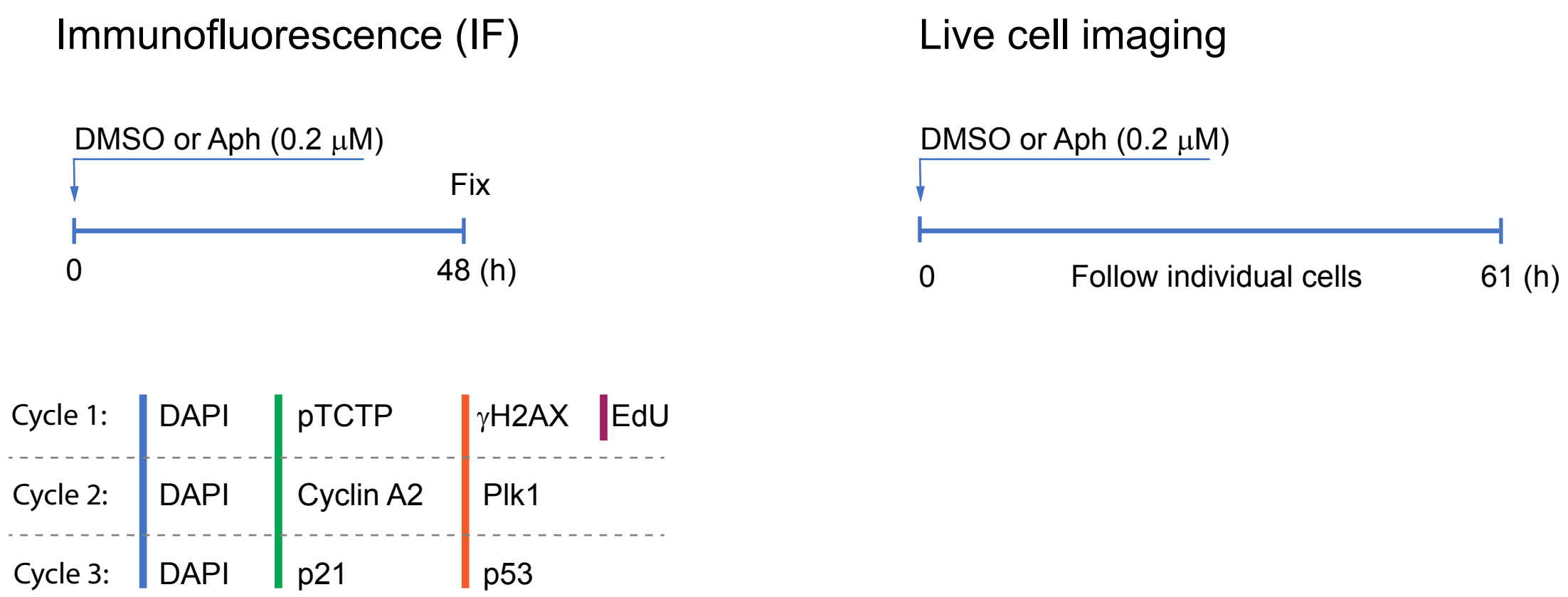

Appendix Figure S2. Comparison of single quantitative immunofluorescence and cumulative microscopy.

A, Figures show quantification of DAPI and Cyclin A2 staining, sorted for estimated cell cycle time. The data for cumulative microscopy comes from an identical experiment setup as is showed in Figure 1. The moving average is calculated for 20 cells. Data shown are representative of at least 3 independent experiments, each performed with technical replicates.

B, Quantification of Cyclin A2 and Plk1 staining, sorted for estimated cell cycle time. The data for cumulative microscopy comes from an identical experiment setup as is showed in Figure 1. The moving average is calculated for 40 cells. Data shown are representative of 2 independent experiments, each performed with technical replicates.

C, Signal distribution for Cyclin A2 in G2 for untreated CHH cells (left panel) and CHH cells treated with aphidicolin (right panel). Signal distribution Comparison of Cumulative IF vs Standard IF is performed by using Kolmogorov–Smirnov (KS) test. Data shown are representative of 2 independent experiments, each performed with technical replicates.

D, setup of cumulative microscopy and live-cell imaging experiments.

Appendix Figure S3

Signal distribution of Cumulative IF at varying exposure times in RPE G2 cells

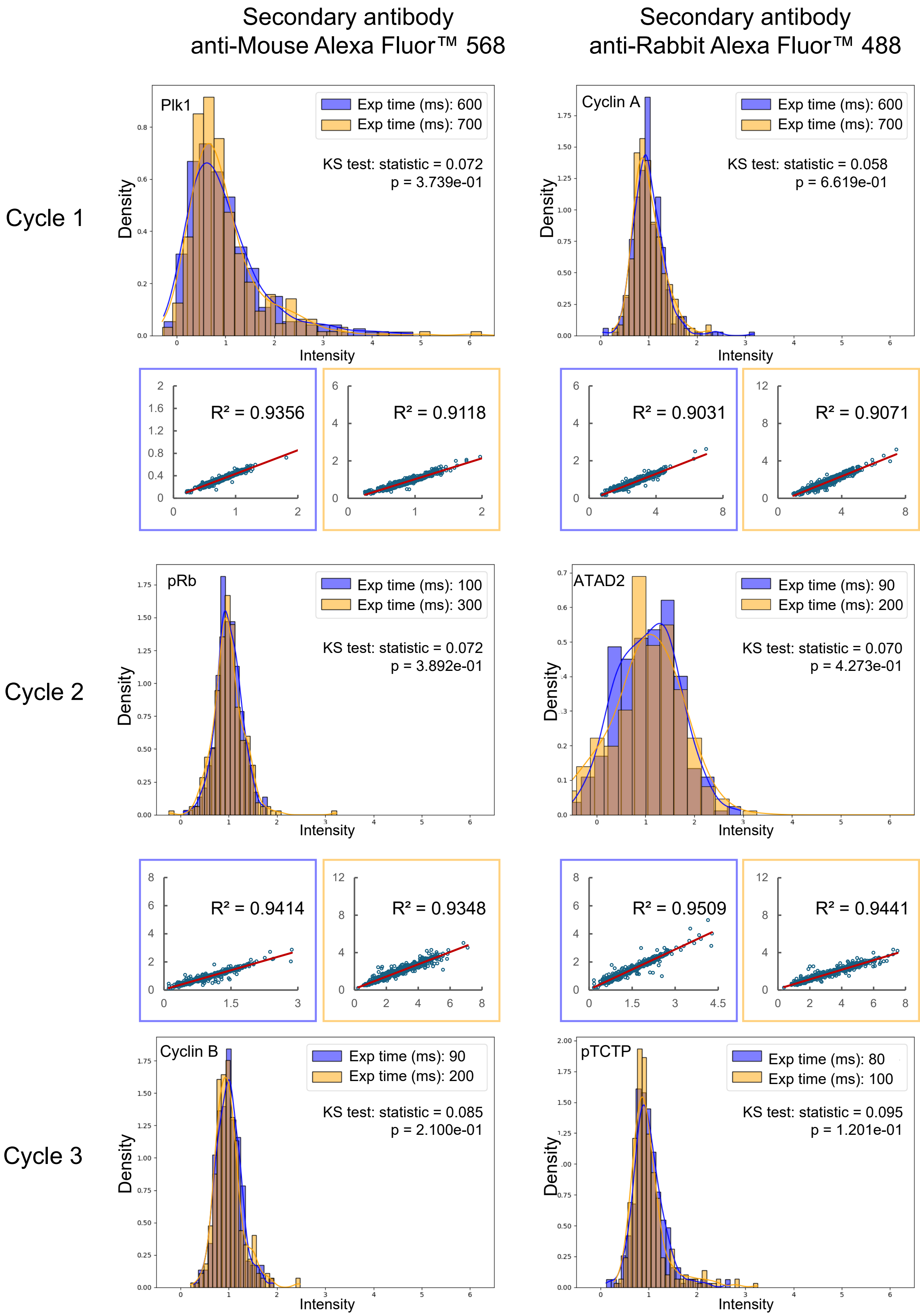

Appendix Figure S3. Signal distribution at varying exposure times

Signal distribution of Cumulative IF at varying exposure times in RPE G2 cells for Plk1, Cyclin A2, pRb, ATAD2, Cyclin B and pTCTP. Signal distribution Comparison is performed by using Kolmogorov–Smirnov (KS) test. Data shown are representative of 3 independent experiments, each performed with technical replicates.
Linear regression was applied for correction using a scaling function, illustrated in the small squares between cycles marked in blue or yellow, corresponding to specific exposure times.

Appendix Figure S4

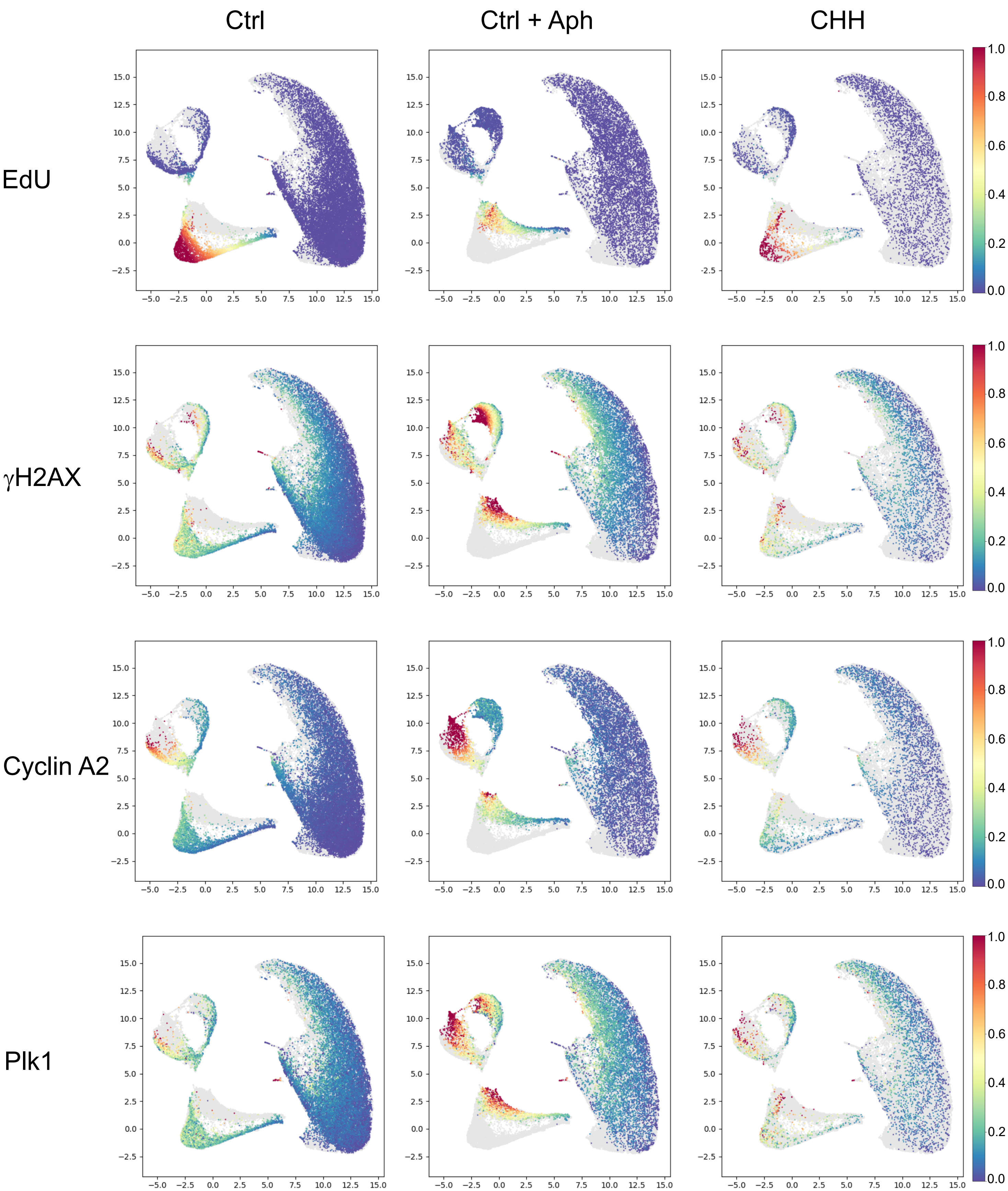

Appendix Figure S4. UMAP of selected cells.

Graphs show UMAP based on data in figure 2, but separated for only control cells treated with DMSO (Ctrl), control cells treated with Aphidicolin (Ctrl + APH), and CHH cells (CHH). Data shown are representative of 2 independent experiments, each performed with technical replicates.

Appendix Figure S5

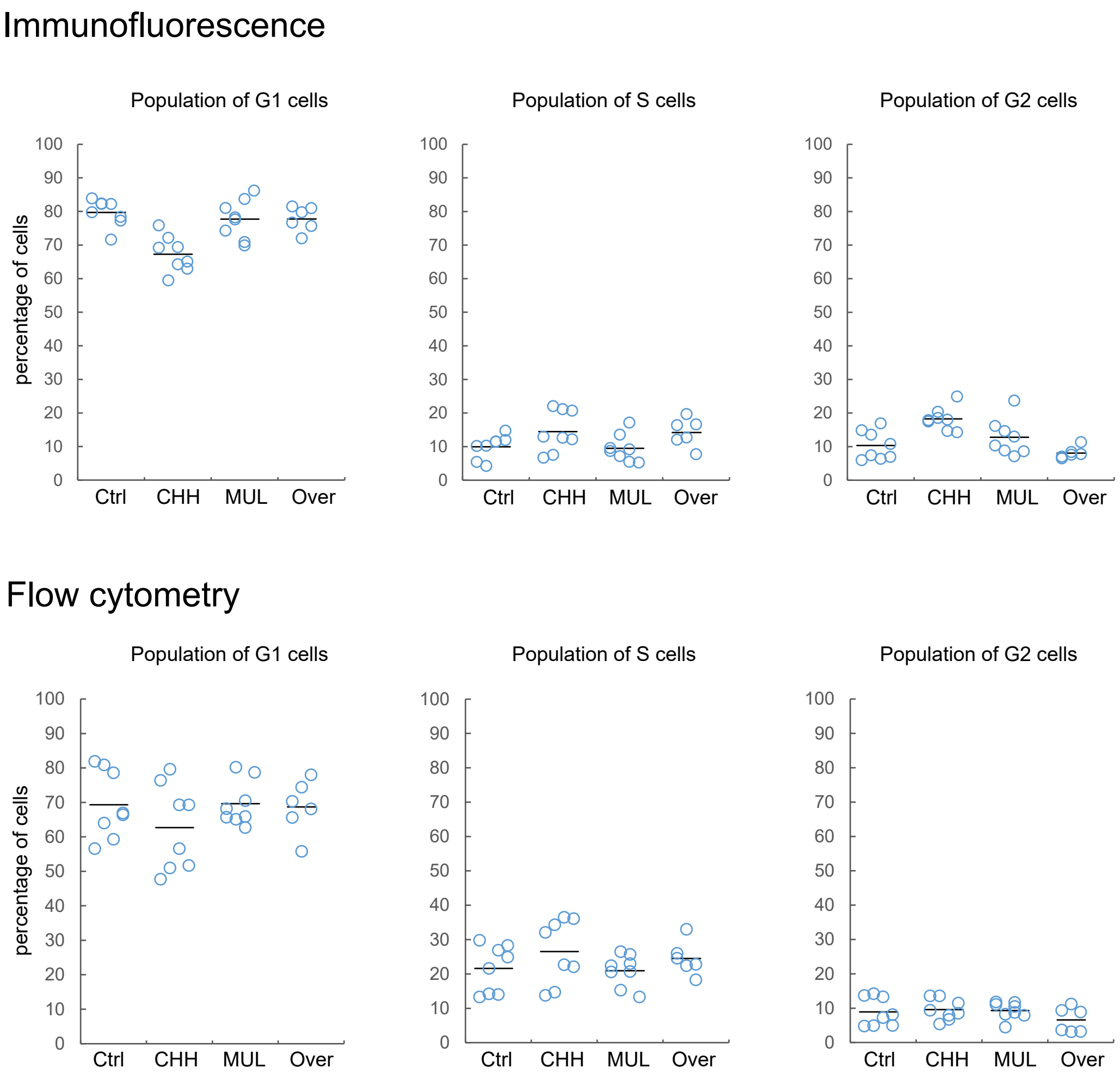

Appendix Figure S5. Independent validation of cell-cycle phase distribution by flow cytometry.

Quantification of G1-, S-, and G2/M-phase populations by immunofluorescence staining (top) and flow cytometric analysis of EdU incorporation (bottom) in Ctrl, CHH, MUL and Over primary fibroblasts. Each dot represents one primary fibroblast sample from an individual donor, measured in duplicate; horizontal bars indicate mean values.

Appendix Figure S6

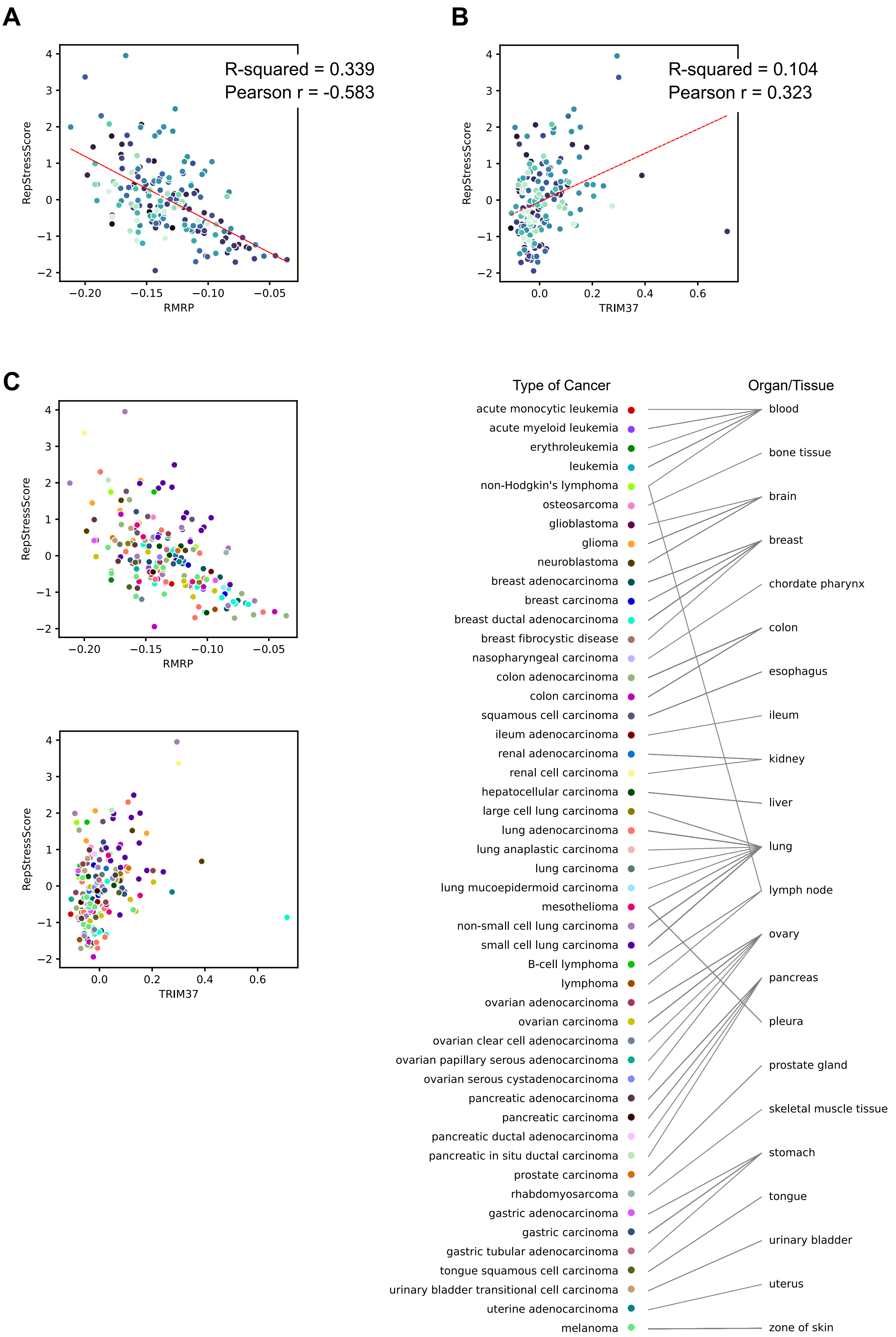

Appendix Figure S6. Correlation between replication stress and expression of RMRP and TRIM37.

(A, B) Scatter plots showing the correlation between replication stress (RepStressScore) and RNA expression levels of RMRP (A) and TRIM37 (B) across 180 cancer cell lines. Red lines indicate linear regression trendlines.

(C) Scatter plots from panels A and B, color-coded by cell line origin.

Appendix Figure S7

A Gating strategy

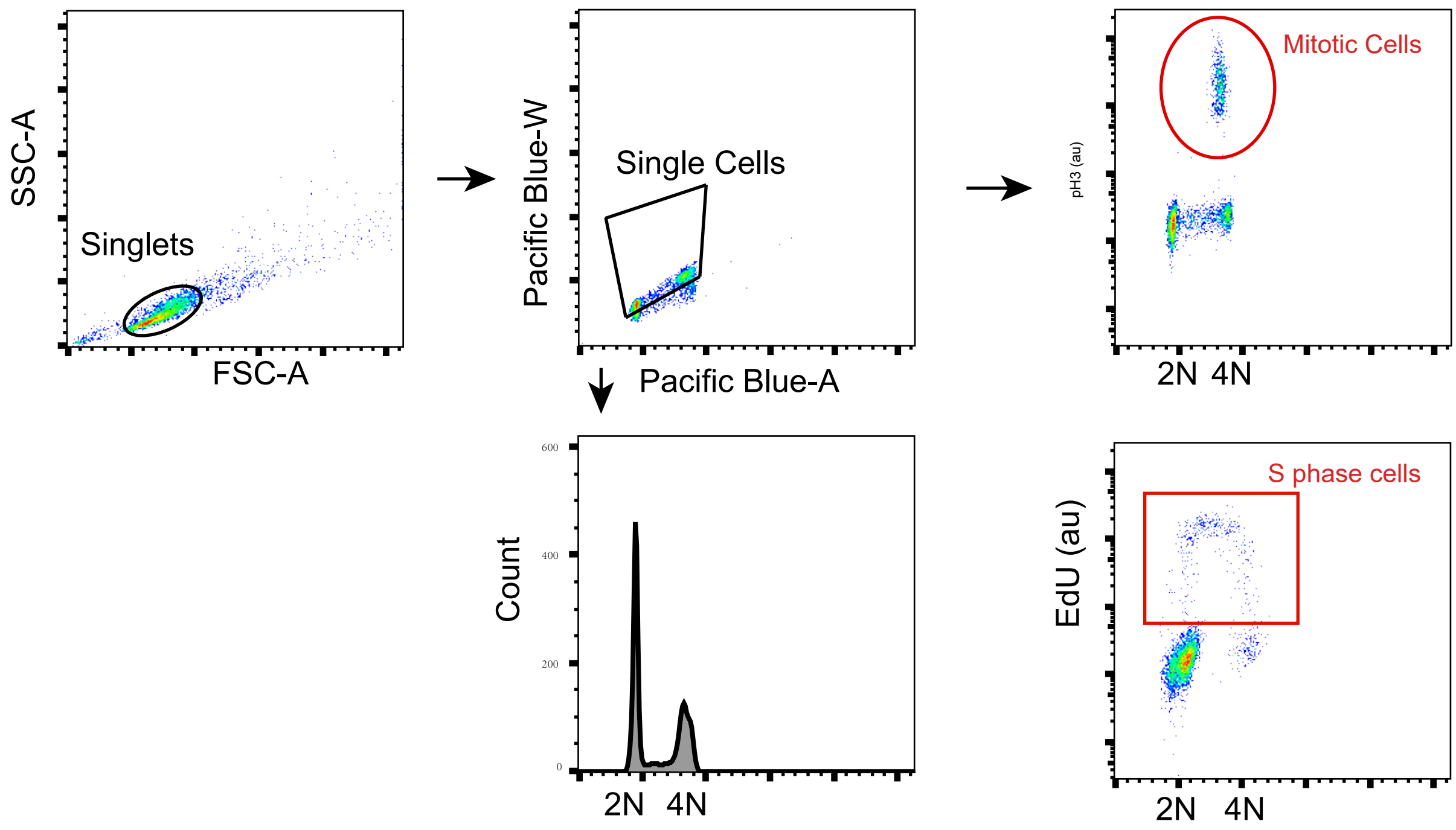

B Compensation controls for flow cytometry

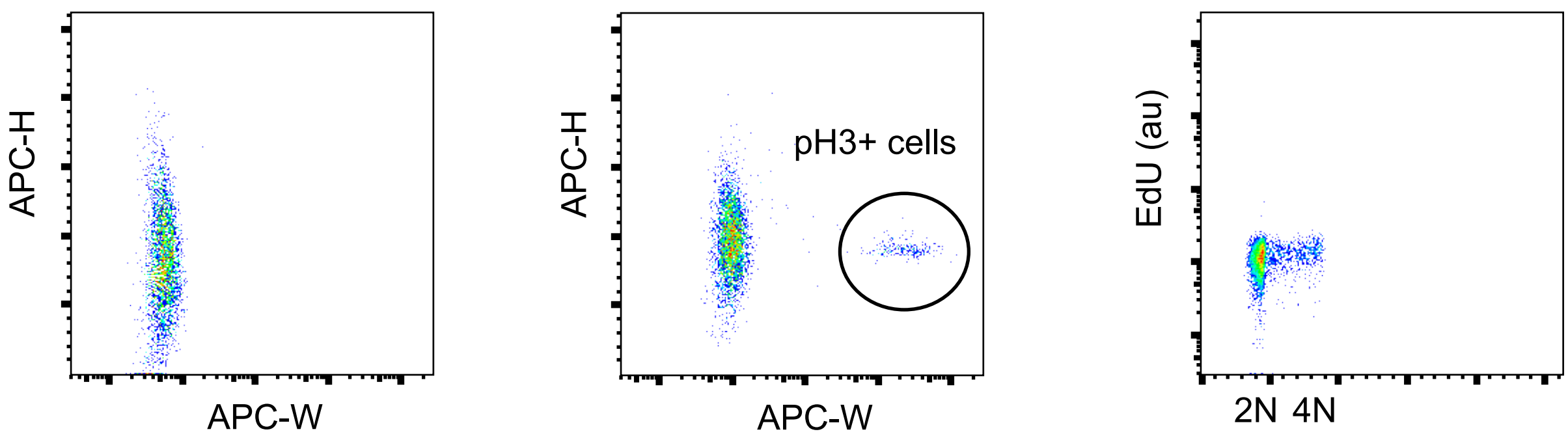

Appendix Figure S7. Representative gating strategy for identification of mitotic or S-phase cells and compensation controls for flow cytometry.

(A) Cells were first gated on the main population according to FSC-A and SSC-A and subsequently filtered for single cells using Pacific Blue-A and Pacific Blue-W. Mitotic cells were defined as the pH3-positive population after gating on DNA content. DNA-content profiles showing the 2N and 4N peaks are presented as reference. S-phase cells were defined as the EdU-positive population.

(B) Representative compensation controls for the relevant fluorescence channels are shown in the lower panels.
