# Supplementary material for: Cumulative microscopy reveals cellular states in fibroblasts from patients with genetic disorders
Source: EMBO Rep. 2026 Jul 30;27(17):5022–38. doi: 10.1038/s44319-026-00859-5 (PMC13554152; doi:10.1038/s44319-026-00859-5)
Supplement: Supplementary file 2 [file 44319_2026_859_MOESM4_ESM.pdf]

EMBO reports

Cumulative microscopy reveals cellular states in fibroblasts from patients with genetic disorders

Karen Akopyan, Zhiyu Hao, Susann Karlberg, Svetlana Vakkilainen, Fulya Taylan, Minna Pekkinen, Outi Mäkitie, and Arne Lindqvist

Corresponding author(s): Arne Lindqvist (arne.lindqvist@ki.se)

Review Timeline:

Transfer Date:	27th Jan 26
Editorial Decision:	5th Feb 26
Revision Received:	8th May 26
Editorial Decision:	10th Jun 26
Revision Received:	21st Jun 26
Accepted:	24th Jun 26

Editor: Esther Schnapp

Transaction Report: This manuscript was transferred to EMBO Reports following peer review at Review Commons.

(Note: With the exception of the correction of typographical or spelling errors that could be a source of ambiguity, letters and reports are not edited. Depending on transfer agreements, referee reports obtained elsewhere may or may not be included in this compilation. Referee reports are anonymous unless the Referee chooses to sign their reports.)

Review #1

1. Evidence, reproducibility and clarity:

Evidence, reproducibility and clarity (Required)

Summary: The study introduces cumulative microscopy, a quantitative method to assess cell cycle and stress markers in individual primary fibroblasts obtained from patients with rare genetic disorders. This approach is compatible with neural network-based analysis, enabling the identification of distinct cellular states.

Major Comments:

1. Clarification of Experimental Replicates
While the authors provide extensive single-cell data (over 96,000 cells from 15 individuals), it is not clearly stated how many times key experiments were independently repeated. Specifying the number of biological and technical replicates would improve confidence in the reproducibility and robustness of the findings.
2. Validation of Cumulative IF with Standard Controls
The authors demonstrate that cumulative microscopy can quantify protein levels by immunofluorescence (IF), but including a control experiment would strengthen confidence in these results. For example, performing IF on cells with no previous staining and comparing the results would help validate the method. This could be done using control versus disease fibroblasts, or a cell line such as RPE under a cell cycle perturbation, like aphidicolin.
3.Clarification of Experimental Details and Quantification Methods
The manuscript notes that cells were seeded in a 96-well imaging plate overnight, but additional details would aid reproducibility. It would be helpful to clarify whether the wells were glass- or plastic-bottom, whether any surface treatment was applied, and how protein levels were quantified (e.g., using nuclear masks or another method).
4. Clarification of Figure Plot Types
The figures should specify the type of plots presented. For example, box plots could indicate median, interquartile range, and outliers, while violin plots could clarify whether the width represents data density and which summary statistics are included. Clear labelling would enhance interpretability and reproducibility (e.g., Figure 4).

Minor Comments:

- The authors should acknowledge in their introduction the work developed by Stallaert and colleagues 2022 (DOI: 10.1016/j.cels.2021.10.007) that maps the dynamics of 48 key human cell cycle proteins using single-cell imaging and computational modelling, providing crucial insights into cell proliferation, arrest, and therapeutic strategies for diseases like cancer.
- Supplementary Figure 6 is never mentioned in the text.
- In Supplementary Figure 1, the panels are labelled as "A - Imaging" and "C - Quantification." Should the second panel be labelled "B - Quantification" instead?
- Two supplementary items (Supplementary Figure 5A and Supplementary Figure 8) are currently presented as figures but consist entirely of tabular data. It would be more appropriate to present these as tables for clarity and proper formatting.

2. Significance:

Significance (Required)

General assessment

This study presents cumulative microscopy, an innovative method for quantitative single-cell analysis of cell cycle and stress markers in primary fibroblasts from patients with rare genetic disorders. Its strengths include high-resolution single-cell data, integration with neural network-based analysis, and the ability to quantify multiple proteins in the same experiment. Limitations include unclear reporting of experimental replicates, missing methodological details, and lack of validation controls, which, if addressed, would make the method more robust. Overall, resolving these issues would enhance reproducibility, clarity, and the impact of this promising technique.

Advance

This study introduces cumulative microscopy, enabling multiplexed single-cell analysis of cell cycle and stress markers in the same experiment-an advance over previous approaches. Compared to related work, it applies this method to primary fibroblasts from patients with rare genetic disorders, providing new mechanistic and functional insights into cellular heterogeneity and stress responses. The study represents both a technical and conceptual advance, offering a framework for integrating high-content imaging with neural network-based analyses.

Audience

This study will primarily appeal to a specialised audience in cell biology, single-cell analysis, and disease modelling, especially researchers studying rare genetic disorders, cell cycle regulation, and stress responses. Its combination of multiplexed imaging and neural network-based analysis may also interest those in computational biology and high-content imaging. Beyond this field, the method could be useful in translational research for understanding disease mechanisms or screening therapeutic interventions.

My expertise

My expertise lies in cell cycle regulation, live cell imaging of single cells, quantitative microscopy, high-content imaging analysis, and molecular modelling. I am confident in evaluating the technical and conceptual aspects of this study, including experimental design, imaging methods, and data analysis.

3. How much time do you estimate the authors will need to complete the suggested revisions:

Estimated time to Complete Revisions (Required)

(Decision Recommendation)

Between 1 and 3 months

4. *Review Commons* values the work of reviewers and encourages them to get credit for their work. Select 'Yes' below to register your reviewing activity at [Web of Science Reviewer Recognition Service] (formerly Publons); note that the content of your review will not be visible on Web of Science.

Web of Science Reviewer Recognition

Yes

Review #2

1. Evidence, reproducibility and clarity:

Evidence, reproducibility and clarity (Required)

Summary

Akopyan et.al. introduces an imaging approach termed cumulative microscopy, in which the same biospecimen is subjected to sequential rounds of staining and imaging. Spatial information for each target protein is then computationally integrated through post-image processing and analysis. The principal advantage claimed by the authors is that this strategy circumvents the need for fluorophore bleaching or antibody stripping between imaging cycles, a step usually required in multiplex microscopy. Using this technique, the authors report that a subset of fibroblasts derived from patients with genetic disorders exhibits replication stress, which may contribute to cancer progression. While the concept is potentially impactful, the study lacks sufficient validation at both the imaging and computational analysis levels. Rigorous methodological and biological validation is essential, and the current version of the manuscript requires substantial revision before it can be considered for publication elsewhere.

Major Comments

1. The manuscript does not present sufficient experimental validation of the proposed methodology. In the authors' workflow, repeated labeling is performed with the same secondary antibody sets. Conventional multiplex immunofluorescence is limited by antibody species compatibility and microscope channel capacity, typically requiring antibody quenching or stripping. In contrast, the authors rely on computational subtraction of prior labeling to permit repeated use of the same secondary antibody sets.
This approach raises several critical concerns:

(A) Although Figure 1 shows single-color images of dTCTP, Cyclin A2, and ATAD2, the key advantage of cumulative microscopy should lie in true multicolor labeling across cycles. If only three proteins are analyzed, conventional three-color immunofluorescence would suffice. The authors should demonstrate at least three cycles of labeling with three different targets per cycle, providing representative pre- and post-correction images, detailed processing workflows, and higher-resolution image data.

(B) The risk of non-specific binding and artifacts introduced by cumulative labeling has not been addressed. Without inactivation or removal of prior antibodies, cross-reactivity and increased background are likely, particularly when secondary antibodies bind to primary antibodies from subsequent cycles of the same species.

(C) The performance of the method when identical fluorophores are reused across cycles with overlapping localization is unclear.

(D) The subtraction strategy requires quantitative validation. The manuscript should present expected levels of under- or over-correction and assess the reliability of this computational step.

2. The manuscript should clarify the rationale for choosing ATAD2, Cyclin A2, and pTCTP in Figure 1B. Their specific biological roles and relevance to the experimental design need to be explicitly explained.

3. According to the Methods, the authors used a 20× NA 0.45 air objective for all experiments. With such a low numerical aperture, it is doubtful that the authors could adequately resolve the detailed characteristics of the markers analyzed. The authors should provide representative raw and corrected images for each marker, along with detailed descriptions of their labeling and imaging strategies, to allow proper evaluation of the method.

4. It is unclear whether cumulative microscopy is applicable to a broad range of targets and whether it can be adapted for higher-resolution imaging, or if it remains constrained to relatively large effective pixel sizes as obtained with a 20× objective. The authors should provide results and performance tests to demonstrate the versatility and limitations of this method.

3. The manuscript lacks methodological details on how the same imaging location is reliably identified across repeated rounds of staining.

4. The claim that cumulative microscopy is quantitative is not adequately substantiated. Adjusting exposure times between imaging cycles compromises the assumption that signal intensity remains linearly proportional to protein abundance within the relevant cellular domains. The authors must clarify how quantitative accuracy is maintained under these conditions and provide comprehensive experimental validation.

5. The calibration procedure is insufficiently described. Key points to address include: How many targets and fluorophore combinations were tested? How was antibody variability (e.g., background, specificity) accounted for? Was calibration assessed on a per-pixel or per-cell basis, and is population-level evaluation adequate for local corrections? Which parameters can or cannot be corrected by the calibration process? Without this information, the conclusions drawn from calibration remain unconvincing.

6. Figure S2 suggests that segmentation is critical, as pixel-by-pixel correlations deteriorate with successive staining rounds. The authors should define thresholds for acceptable error, quantify how errors propagate across cycles, and clarify how pixel assignments are maintained when subsequent cycles introduce new, bright cellular features that may obscure prior signals. Furthermore, it remains unclear whether this approach is limited to low-resolution, large-pixel images (e.g., 20×) or whether it is robust enough to perform reliably with high-resolution imaging (e.g., 100× objectives with ~60 nm effective pixel size).

7. The prevalence and significance of CHH and MUL should be described more thoroughly

in the Introduction. The rationale for selecting these two disorders as models must be clearly justified.

8. In Figures 1C and 1D, the cumulative IF data relative to mitotic timing should be labeled as estimated time, not as actual time measured by live-cell imaging. Presenting it otherwise is misleading and creates significant confusion.

9. The Methods section lacks information on the number of cells analyzed, which is essential for accurate quantification. Both the estimation of cell cycle progression and the determination of population frequencies require sufficiently large sample sizes to ensure reliability, yet such evaluations are missing. Does the method provide single-cell accuracy? What is the associated error as a function of sample size? How many cells are required for robust and accurate analysis?

10. For Figures 2B-C, it is not possible to evaluate accuracy without detailed description of how cell cycle stages were experimentally validated and how computational models were confirmed.

11. The choice of pTCTP, Cyclin A2, p21, γH2AX, Plk1, p53, and EdU in Figure 2 requires justification. Their expected temporal dynamics, localization patterns, and relevance to the study design must be described. The results should be interpreted beyond signal intensity, addressing whether cumulative microscopy accurately recapitulates known biological distributions.

12. The claim that CHH cells exhibit replication stress must be quantitatively supported. The authors should measure γH2AX foci per cell. In addition, they must demonstrate that these foci can indeed be visualized with a 20× air objective, typically, such structures are resolved only with higher-NA, high-magnification objectives. If not, the authors must validate how cumulative microscopy reliably detects these signals.

12. The assertion that p53/p21 upregulation corresponds to G1 arrest or G2 exit is not adequately supported. Additional experiments are needed to distinguish cell cycle arrest from ongoing progression.

13.To substantiate the claim that MUL and CHH cells activate a G2 checkpoint, the authors should perform flow cytometry as well as live-cell imaging with FUCCI or alternative markers to specifically assess G2 enrichment, in addition to immunofluorescence assays for checkpoint activation. Bright-field live imaging at 1-hour intervals is insufficient to support the authors' conclusion.

14. The UMAP plots require clarification. Specifically: What datasets were combined? How were they integrated and validated? How are non-dividing populations (10-30% in CHH and MUL) represented in the plots? Do these cells correspond to G1, S, or G0? Do they lack γH2AX? The accuracy of the model must be evaluated against these populations.

15. The effects of low-dose aphidicolin should be directly compared with those of standard concentrations to determine whether the observed results are simply due to partial

polymerase inhibition. If this is the case, all statements regarding aphidicolin must be interpreted with appropriate caution.
16. The proportion of G1, S, and G2 cells in each line (control, CHH, MUL) should be confirmed by an independent method, such as flow cytometry or alternative methods, to support the imaging data.
17. Differences in antibody staining efficiency and protein expression between cell types must be taken into account. Proper optimization and validation, along with assessment of target protein and RNA levels, are required to ensure fair and reliable comparisons.
18. The reported classification accuracies (31-41% for CHH and MUL; ~50% phase assignment) are too low to be scientifically meaningful. The authors should critically evaluate whether such performance levels can support their conclusions.

Minor comments:

1. Both results, Figures and methods section require substantial rewriting to include details.

2. Significance:

Significance (Required)

The principal significance of this study lies in the development of a method for multiplex immunofluorescence microscopy that avoids fluorophore bleaching and antibody stripping between successive labeling and imaging cycles. By circumventing these labor-intensive processes, the approach offers a substantial advantage to researchers by reducing overall experimental time. However, the study is limited by insufficient performance evaluation and a lack of comprehensive data to assess the robustness, capacity, limitations, and scalability of the method. Addressing these gaps will be essential to enhance its accessibility and acceptance across diverse research fields.

3. How much time do you estimate the authors will need to complete the suggested revisions:

Estimated time to Complete Revisions (Required)

(Decision Recommendation)

Between 3 and 6 months

4. *Review Commons* values the work of reviewers and encourages them to get credit for their work. Select 'Yes' below to register your reviewing activity at Web of Science

Reviewer Recognition Service (formerly Publons); note that the content of your review will not be visible on Web of Science.

Web of Science Reviewer Recognition

Yes

Review #3

1. Evidence, reproducibility and clarity:

Evidence, reproducibility and clarity (Required)

Summary:

Akopyan et al have developed cumulative microscopy to analyze repeated rounds of immunofluorescent stainings without the need of quenching, stripping or using secondary antibodies from different animals. They demonstrate the power of the system by performing cell cycle analyses in individual primary fibroblasts derived from patients. Using this approach, they showed different clustering of cells from patients with two different disorders. The authors conclude that multiplex microscopy is a powerful, yet straightforward method for multiplex immunofluorescence, applicable in a wide range of assays.

Major comments:

1. The authors address several potential pitfalls, such as slight movement of the samples from one imaging cycle to the next, dealing with bleaching during repeated imaging cycles, technical variability, changing exposure times. This thorough consideration and testing make the results very convincing.
2. The methods used are well described and statistical analyses are adequate. It is not clear to me whether the scripts are made publicly available; if yes, a link would be appreciated by all users.
3. It would be interesting to identify mitotic cells, for example based on the P-TCPT staining combined with DAPI (if possible from the existing data; I am not suggesting redoing analyses using other mitotic markers). Given that MUL cells contain mutations in TRIM37, they might be identifiable based on mitotic delays and errors, which would also be consistent with the increased p53 and p21 staining.

Minor comments:

1. The figures are very clear.
2. I don't think the accumulation of the MUL cells in the UMAP (referred to on p 6 line 5) is shown.
3. I believe the text would benefit from some more detailed explanations, depending on the target audience. In theory the method can be used for just about any cellular process, meaning not all interested readers will be experts in the cell cycle. Specifically, it would be useful to explain the choice of targets the authors stain for and why they are appropriate markers for cell cycle studies. It is not clear what the authors mean by "stress markers".
4. The same applies for the trajectories shown on Figure 3 - how were these defined?
5. The term "replication stress score" is not explained. Please describe the key points in the main text, or refer the reader to the detailed description in the Methods.

2. Significance:

Significance (Required)

- The nature of the advance is mainly technical, this is an approach that has the potential to lead to many conceptual advances.
- The major strength of the approach is that it does not requires very specialized equipment and thus it can be used in most laboratories.
- The authors did a very good job of introducing existing methods and papers presenting previous studies. It could be relevant to also discuss spectral flowcytometry and microscopy as alternative methods for multiplexing (maybe pointing out pros and contras).
- I think very many researchers would be interested in the reported approach, since the method is applicable for any biological question where immunofluorescence can be employed.
- My expertise: I have a solid background in the cell-cycle field and consider myself reasonably well-versed in immunofluorescence and microscopy. However, I am no expert in large-scale data analyses.

3. How much time do you estimate the authors will need to complete the suggested revisions:

Estimated time to Complete Revisions (Required)

(Decision Recommendation)

Less than 1 month

4. *Review Commons* values the work of reviewers and encourages them to get credit for their work. Select 'Yes' below to register your reviewing activity at Web of Science Reviewer Recognition Service (formerly Publons); note that the content of your review will not be visible on Web of Science.

Web of Science Reviewer Recognition

Yes

Revision Plan

Manuscript number: RC-2025-03150

Corresponding author(s): Lindqvist, Arne

1. General Statements [optional]

We are pleased that the reviewers were positive to the potential of cumulative microscopy. We completely agree to all points raised by reviewers 1 and 3 and are incorporating the changes. We were also very pleased by statements such as "This thorough consideration and testing make the results very convincing".

We agree to some of the points of reviewer 2, but not to all of them. The points can very broadly be summarized as: more detailed descriptions, control experiments, and expanded experiments including a focus on high magnification. We agree to making more detailed descriptions throughout. We also agree to the control experiments suggested by reviewer 2, except in the cases we believe that the data was already present in the original version of the manuscript or that they stem from a misunderstanding. In all cases we are currently adjusting the text to increase detail and clarity. Two points of reviewer 2 we feel are outside of the scope of this manuscript. The first point is to assess cumulative microscopy for high-resolution imaging, next to the current focus on quantitative imaging of cellular signals (or image cytometry/QIBC). This would take long time, and we argue that it is outside of the scope of this manuscript. The second point is to assess RNA levels in cells. We argue that this is not necessary for the conclusions and is outside of the scope of this manuscript.

2. Description of the planned revisions

Insert here a point-by-point reply that explains what revisions, additional experimentations and analyses are planned to address the points raised by the referees.

Reviewer #1 (Evidence, reproducibility and clarity (Required)):

#Summary: The study introduces cumulative microscopy, a quantitative method to assess cell cycle and stress markers in individual primary fibroblasts obtained from patients with rare genetic disorders. This approach is compatible with neural network-based analysis, enabling the identification of distinct cellular states.

#Major Comments:
1. Clarification of Experimental Replicates
While the authors provide extensive single-cell data (over 96,000 cells from 15 individuals), it is not clearly stated how many times key experiments were independently repeated. Specifying the number of biological and technical replicates would improve confidence in the reproducibility and robustness of the findings.

We agree and apologize for this mistake. We will include details on biological and technical replicates for all experiments.

2. Validation of Cumulative IF with Standard Controls
The authors demonstrate that cumulative microscopy can quantify protein levels by immunofluorescence (IF), but including a control experiment would strengthen confidence in these results. For example, performing IF on cells with no previous staining and comparing the results would help validate the method. This could be done using control versus disease fibroblasts, or a cell line such as RPE under a cell cycle perturbation, like aphidicolin.

We agree that such a comparison can be valuable also on the perturbed states. In addition to the existing comparison on unperturbed RPE cells in fig 1, we will include such an experiment for patient fibroblasts.

3.Clarification of Experimental Details and Quantification Methods
The manuscript notes that cells were seeded in a 96-well imaging plate overnight, but additional details would aid reproducibility. It would be helpful to clarify whether the wells were glass- or plastic-bottom, whether any surface treatment was applied, and how protein levels were quantified (e.g., using nuclear masks or another method).

We agree and will extend the description on experimental conditions.

4. Clarification of Figure Plot Types
The figures should specify the type of plots presented. For example, box plots could indicate median, interquartile range, and outliers, while violin plots could clarify whether the width represents data density and which summary statistics are included. Clear labelling would enhance interpretability and reproducibility (e.g., Figure 4).

We agree and will modify the figures to include these aspects.

#Minor Comments:
- The authors should acknowledge in their introduction the work developed by Stallaert and colleagues 2022 (DOI: 10.1016/j.cels.2021.10.007) that maps the dynamics of 48 key human cell cycle proteins using single-cell imaging and computational modelling, providing crucial insights

into cell proliferation, arrest, and therapeutic strategies for diseases like cancer.
- Supplementary Figure 6 is never mentioned in the text.
- In Supplementary Figure 1, the panels are labelled as "A - Imaging" and "C - Quantification." Should the second panel be labelled "B - Quantification" instead?
- Two supplementary items (Supplementary Figure 5A and Supplementary Figure 8) are currently presented as figures but consist entirely of tabular data. It would be more appropriate to present these as tables for clarity and proper formatting.

We agree to all minor comments and will make changes accordingly.

Reviewer #1 (Significance (Required)):

#General assessment
This study presents cumulative microscopy, an innovative method for quantitative single-cell analysis of cell cycle and stress markers in primary fibroblasts from patients with rare genetic disorders. Its strengths include high-resolution single-cell data, integration with neural network-based analysis, and the ability to quantify multiple proteins in the same experiment. Limitations include unclear reporting of experimental replicates, missing methodological details, and lack of validation controls, which, if addressed, would make the method more robust. Overall, resolving these issues would enhance reproducibility, clarity, and the impact of this promising technique.

#Advance
This study introduces cumulative microscopy, enabling multiplexed single-cell analysis of cell cycle and stress markers in the same experiment-an advance over previous approaches. Compared to related work, it applies this method to primary fibroblasts from patients with rare genetic disorders, providing new mechanistic and functional insights into cellular heterogeneity and stress responses. The study represents both a technical and conceptual advance, offering a framework for integrating high-content imaging with neural network-based analyses.

#Audience
This study will primarily appeal to a specialised audience in cell biology, single-cell analysis, and disease modelling, especially researchers studying rare genetic disorders, cell cycle regulation, and stress responses. Its combination of multiplexed imaging and neural network-based analysis may also interest those in computational biology and high-content imaging. Beyond this field, the method could be useful in translational research for understanding disease mechanisms or screening therapeutic interventions.

#My expertise
My expertise lies in cell cycle regulation, live cell imaging of single cells, quantitative microscopy, high-content imaging analysis, and molecular modelling. I am confident in evaluating the technical and conceptual aspects of this study, including experimental design, imaging methods, and data

analysis.

Reviewer #2 (Evidence, reproducibility and clarity (Required)):

Summary
Akopyan et.al. introduces an imaging approach termed cumulative microscopy, in which the same biospecimen is subjected to sequential rounds of staining and imaging. Spatial information for each target protein is then computationally integrated through post-image processing and analysis. The principal advantage claimed by the authors is that this strategy circumvents the need for fluorophore bleaching or antibody stripping between imaging cycles, a step usually required in multiplex microscopy. Using this technique, the authors report that a subset of fibroblasts derived from patients with genetic disorders exhibits replication stress, which may contribute to cancer progression. While the concept is potentially impactful, the study lacks sufficient validation at both the imaging and computational analysis levels. Rigorous methodological and biological validation is essential, and the current version of the manuscript requires substantial revision before it can be considered for publication elsewhere.

Major Comments
1. The manuscript does not present sufficient experimental validation of the proposed methodology. In the authors' workflow, repeated labeling is performed with the same secondary antibody sets. Conventional multiplex immunofluorescence is limited by antibody species compatibility and microscope channel capacity, typically requiring antibody quenching or stripping. In contrast, the authors rely on computational subtraction of prior labeling to permit repeated use of the same secondary antibody sets.
This approach raises several critical concerns:
(A) Although Figure 1 shows single-color images of dTCTP, Cyclin A2, and ATAD2, the key advantage of cumulative microscopy should lie in true multicolor labeling across cycles. If only three proteins are analyzed, conventional three-color immunofluorescence would suffice. The authors should demonstrate at least three cycles of labeling with three different targets per cycle, providing representative pre- and post-correction images, detailed processing workflows, and higher-resolution image data.

We agree that multi-colour labeling is beneficial to increase the number of targets. In the large experiment with patient fibroblasts, we therefore use four colour acquisition and two secondary antibodies (setup described in figure S5). We will include an experiment with three different secondary antibodies and increase the detail of the description of the workflow, as well as include more example images.

(D) The subtraction strategy requires quantitative validation. The manuscript should present expected levels of under- or over-correction and assess the reliability of this computational step.

We agree and already do this. In figure 1D we indicate the expected levels of under- and over-correction. Figure S3 show how the calculations are performed and indicate statistics based on real data. In figure 1D and figure S4 we compare the results to standard quantitative immunofluorescence and to live-cell imaging of tagged protein and find similar results. We further show that the expected variation is low compared to cell-cell variation.

2. The manuscript should clarify the rationale for choosing ATAD2, Cyclin A2, and pTCTP in Figure 1B. Their specific biological roles and relevance to the experimental design need to be explicitly explained.

We agree, this section will be extended in the revised manuscript. Briefly, they were selected as they show characteristic and distinct changes through the cell cycle. These distinct changes allows for assessment for the reliability of the method. For example, ATAD2 decreases through G2 phase, ending at very low signal, whereas pTCTP and Cyclin A2 in the previous imaging cycles increase throughout G2 (figure 1C). Thus, when ATAD2 is very low, the preceding stainings are very high. The selection of antibodies thus enables us to show that the signal in previous cycles does not contribute significantly to the signal in the following cycle.

3. According to the Methods, the authors used a 20× NA 0.45 air objective for all experiments. With such a low numerical aperture, it is doubtful that the authors could adequately resolve the detailed characteristics of the markers analyzed. The authors should provide representative raw and corrected images for each marker, along with detailed descriptions of their labeling and imaging strategies, to allow proper evaluation of the method.

We will increase the detail of the description on how the experiment was performed and provide images of all stainings.

3. The manuscript lacks methodological details on how the same imaging location is reliably identified across repeated rounds of staining.

We will include further details.

4. The claim that cumulative microscopy is quantitative is not adequately substantiated. Adjusting exposure times between imaging cycles compromises the assumption that signal intensity remains linearly proportional to protein abundance within the relevant cellular domains. The authors must

Review COMMONS

clarify how quantitative accuracy is maintained under these conditions and provide comprehensive experimental validation.

We believe there is a misunderstanding on how the method is performed here? Each antibody staining is acquired seperately and calculated seperately and the same acquisition time is used for one antibody staining. The abundance is thus estimated independently per antibody staining. This is similar to a single round of quantitative IF with two colours – the quantification of each colour/channel is separate and exposure conditions can be different between the two colours/channels. We will clarify this further in the manuscript. The exposure time may however affect the signal-to-noise ratio within one antibody staining. We will include an experiment with parallel acquisition and varying exposure conditions to provide an assessment.

5. The calibration procedure is insufficiently described. Key points to address include: How many targets and fluorophore combinations were tested? How was antibody variability (e.g., background, specificity) accounted for? Was calibration assessed on a per-pixel or per-cell basis, and is population-level evaluation adequate for local corrections? Which parameters can or cannot be corrected by the calibration process? Without this information, the conclusions drawn from calibration remain unconvincing.

We will increase the detail of description for these questions.

7. The prevalence and significance of CHH and MUL should be described more thoroughly in the Introduction. The rationale for selecting these two disorders as models must be clearly justified.

We agree and will include such an description.

8. In Figures 1C and 1D, the cumulative IF data relative to mitotic timing should be labeled as estimated time, not as actual time measured by live-cell imaging. Presenting it otherwise is misleading and creates significant confusion.

We agree and will reformulate the labeling to clarify that the live cell imaging data is measured time and the immunofluorescence data is estimated time.

9. The Methods section lacks information on the number of cells analyzed, which is essential for accurate quantification. Both the estimation of cell cycle progression and the determination of population frequencies require sufficiently large sample sizes to ensure reliability, yet such evaluations are missing. Does the method provide single-cell accuracy? What is the associated error as a function of sample size? How many cells are required for robust and accurate analysis?

We agree and will include this information.

10. For Figures 2B-C, it is not possible to evaluate accuracy without detailed description of how cell cycle stages were experimentally validated and how computational models were confirmed.

We will expand the description of these steps.

11. The choice of pTCTP, Cyclin A2, p21, γH2AX, Plk1, p53, and EdU in Figure 2 requires justification. Their expected temporal dynamics, localization patterns, and relevance to the study design must be described. The results should be interpreted beyond signal intensity, addressing whether cumulative microscopy accurately recapitulates known biological distributions.

We will expand the description of the studied proteins and their expected dynamics and show graphs of individual stainings from the patient fibroblast data.

12. The claim that CHH cells exhibit replication stress must be quantitatively supported. The authors should measure γH2AX foci per cell. In addition, they must demonstrate that these foci can indeed be visualized with a 20× air objective, typically, such structures are resolved only with higher-NA, high-magnification objectives. If not, the authors must validate how cumulative microscopy reliably detects these signals.

The foci can be visualized. We will include a new figure showing γH2AX foci and a quantification of the number of foci.

13.To substantiate the claim that MUL and CHH cells activate a G2 checkpoint, the authors should perform flow cytometry as well as live-cell imaging with FUCCI or alternative markers to specifically assess G2 enrichment, in addition to immunofluorescence assays for checkpoint activation. Bright-field live imaging at 1-hour intervals is insufficient to support the authors' conclusion.

We are performing flow cytometry to investigate cell cycle phase enrichment in patient cells. We would in addition like to point out that we refer in the text to our previous study (Vakkalainen et al 2019), in which we perform a detailed investigation of G2 delay in CHH cells using EdU pulse-chase.

14. The UMAP plots require clarification. Specifically: What datasets were combined? How were they integrated and validated? How are non-dividing populations (10-30% in CHH and MUL) represented in the plots? Do these cells correspond to G1, S, or G0? Do they lack γH2AX? The accuracy of the model must be evaluated against these populations.

We will increase the detail of description of the construction of UMAPs and expand the discussion on which regions contribute to non-dividing cells.

15. The effects of low-dose aphidicolin should be directly compared with those of standard concentrations to determine whether the observed results are simply due to partial polymerase inhibition. If this is the case, all statements regarding aphidicolin must be interpreted with appropriate caution.

The low-dose Aphidicolin was chosen to prevent a full block of all DNA replication, as such a block is unlikely to occur in patient cells that can proliferate. We will add a description that low-dose aphidicolin creates replication stress that is distinct from a stress that would occur from a complete block of DNA replication.

16. The proportion of G1, S, and G2 cells in each line (control, CHH, MUL) should be confirmed by an independent method, such as flow cytometry or alternative methods, to support the imaging data.

We will add a new figure showing flow cytometry of the patient cells.

18. The reported classification accuracies (31-41% for CHH and MUL; ~50% phase assignment) are too low to be scientifically meaningful. The authors should critically evaluate whether such performance levels can support their conclusions.

We respectfully disagree. The classifications in figure 2 are a starting point for the further studies, with the detailed analysis in figures 3 and 4. Further, they fit well with our finding that not all patient cells show replication stress. Rather, it would have been surprising if all CHH and MUL cells would have been identified solely based on expression of cell cycle and stress markers. We will expand the description of why we believe these data are coherent.

Minor comments:
1. Both results, Figures and methods section require substantial rewriting to include details.

We will increase the level of detail in these sections.
Reviewer #2 (Significance (Required)):

The principal significance of this study lies in the development of a method for multiplex immunofluorescence microscopy that avoids fluorophore bleaching and antibody stripping between successive labeling and imaging cycles. By circumventing these labor-intensive processes, the approach offers a substantial advantage to researchers by reducing overall experimental time. However, the study is limited by insufficient performance evaluation and a lack

of comprehensive data to assess the robustness, capacity, limitations, and scalability of the method. Addressing these gaps will be essential to enhance its accessibility and acceptance across diverse research fields.

Reviewer #3 (Evidence, reproducibility and clarity (Required)):

Summary:
Akopyan et al have developed cumulative microscopy to analyze repeated rounds of immunofluorescent stainings without the need of quenching, stripping or using secondary antibodies from different animals. They demonstrate the power of the system by performing cell cycle analyses in individual primary fibroblasts derived from patients. Using this approach, they showed different clustering of cells from patients with two different disorders. The authors conclude that multiplex microscopy is a powerful, yet straightforward method for multiplex immunofluorescence, applicable in a wide range of assays.

Major comments:
1) The authors address several potential pitfalls, such as slight movement of the samples from one imaging cycle to the next, dealing with bleaching during repeated imaging cycles, technical variability, changing exposure times. This thorough consideration and testing make the results very convincing.

2) The methods used are well described and statistical analyses are adequate. It is not clear to me whether the scripts are made publicly available; if yes, a link would be appreciated by all users.

Scripts are available at https://github.com/KAkopyan/CumulativeMicroscopy

3) It would be interesting to identify mitotic cells, for example based on the P-TCPT staining combined with DAPI (if possible from the existing data; I am not suggesting redoing analyses using other mitotic markers). Given that MUL cells contain mutations in TRIM37, they might be identifiable based on mitotic delays and errors, which would also be consistent with the increased p53 and p21 staining.

We agree and will add a new figure panel showing mitotic cells. We indeed find an enrichment of MUL cells in mitosis.

Minor comments:
1) The figures are very clear.
2) I don't think the accumulation of the MUL cells in the UMAP (referred to on p 6 line 5) is shown.
3) I believe the text would benefit from some more detailed explanations, depending on the target audience. In theory the method can be used for just about any cellular process, meaning not all

interested readers will be experts in the cell cycle. Specifically, it would be useful to explain the choice of targets the authors stain for and why they are appropriate markers for cell cycle studies. It is not clear what the authors mean by "stress markers".
4) The same applies for the trajectories shown on Figure 3 - how were these defined?
5) The term "replication stress score" is not explained. Please describe the key points in the main text, or refer the reader to the detailed description in the Methods.

We agree to all minor comments and will modify the manuscript accordingly.

Reviewer #3 (Significance (Required)):

Significance
- The nature of the advance is mainly technical, this is an approach that has the potential to lead to many conceptual advances.
- The major strength of the approach is that it does not requires very specialized equipment and thus it can be used in most laboratories.
- The authors did a very good job of introducing existing methods and papers presenting previous studies. It could be relevant to also discuss spectral flowcytometry and microscopy as alternative methods for multiplexing (maybe pointing out pros and contras).
- I think very many researchers would be interested in the reported approach, since the method is applicable for any biological question where immunofluorescence can be employed.
- My expertise: I have a solid background in the cell-cycle field and consider myself reasonably well-versed in immunofluorescence and microscopy. However, I am no expert in large-scale data analyses.

3. Description of the revisions that have already been incorporated in the transferred manuscript

Please insert a point-by-point reply describing the revisions that were already carried out and included in the transferred manuscript. If no revisions have been carried out yet, please leave this section empty.

4. Description of analyses that authors prefer not to carry out

Please include a point-by-point response explaining why some of the requested data or additional analyses might not be necessary or cannot be provided within the scope of a revision. This can be due to time or resource limitations or in case of disagreement about the necessity of such additional data given the scope of the study. Please leave empty if not applicable.

Reviewer #2

(B) The risk of non-specific binding and artifacts introduced by cumulative labeling has not been addressed. Without inactivation or removal of prior antibodies, cross-reactivity and increased background are likely, particularly when secondary antibodies bind to primary antibodies from subsequent cycles of the same species.

We respectfully disagree. The method inherently quantifies the change in background when secondary antibodies cross-react or bind to primary antibodies from previous cycles. This is in contrast to most existing methods, and we believe the aspect lifted by the reviewer is therefore an advantage of cumulative microscopy. The description of how the changing background is quantified is described in supplementary text and shown in figures S1 to S4. Figure S3 shows a quantification of the uncertainty introduced due to correction for changing background. We will modify the text to make this more clear.

(C) The performance of the method when identical fluorophores are reused across cycles with overlapping localization is unclear.

We respectfully disagree. In figure 1 we show that three targets with overlapping localisation can be separated. We assess this by comparison to single-round quantitative IF and to expression during live-cell imaging of a fluorescently tagged protein.

4. It is unclear whether cumulative microscopy is applicable to a broad range of targets and whether it can be adapted for higher-resolution imaging, or if it remains constrained to relatively large effective pixel sizes as obtained with a 20× objective. The authors should provide results and performance tests to demonstrate the versatility and limitations of this method.

We agree, we do not know where the borders are in terms of high-resolution imaging. This is a relevant path, and we are initiating a project to test cumulative microscopy for various super-resolution techniques, but that will take time and we feel that a full test of the limits for resolution is outside of the scope of this manuscript. This manuscript validates the method for quantification of cellular signals (image cytometry/QIBC), for which we believe it is a highly useful method.

6. Figure S2 suggests that segmentation is critical, as pixel-by-pixel correlations deteriorate with successive staining rounds. The authors should define thresholds for acceptable error, quantify how errors propagate across cycles, and clarify how pixel assignments are maintained when subsequent cycles introduce new, bright cellular features that may obscure prior signals. Furthermore, it remains unclear whether this approach is limited to low-resolution, large-pixel images (e.g., 20×) or whether it is robust enough to perform reliably with high-resolution imaging (e.g., 100× objectives with ~60 nm effective pixel size).

We agree that segmentation is critical, but disagree with the reviewer that correlations deteriorate with successive staining rounds and that errors should propagate. First, segmentation is independently performed on all cycles. Second, each staining round uses an independent background, based on the antibodies present in the last staining round plus one round with secondary antibody only. Therefore, errors do not propagate through cycles, which is one of the advantages of cumulative microscopy. We will increase the description of this in the manuscript.

12. The assertion that p53/p21 upregulation corresponds to G1 arrest or G2 exit is not adequately supported. Additional experiments are needed to distinguish cell cycle arrest from ongoing progression.

We agree that mere p53/p21 levels are not sufficient to distinguish arrest for ongoing progression. We will expand the description of how the regions G2 exit and G1 stress in figure 4 are estimated. Briefly, G2 exit is characterized by 4n DNA content and an absence of Cyclin A2, whereas G1 stress is characterized by 2n DNA content, p53 and p21 expression, in addition to increased cell size. We will reformulate and clarify in the text that G1 stress does not necessarily mean that cell cycle progression is blocked.

17. Differences in antibody staining efficiency and protein expression between cell types must be taken into account. Proper optimization and validation, along with assessment of target protein and RNA levels, are required to ensure fair and reliable comparisons.

We would like to emphasize that we in this manuscript only directly compares the same cell types. We also believe that assessing RNA levels here would be outside of the scope for this manuscript, and that RNA measurements are not required for conclusions based on protein staining. RNA levels for CHH cells were assessed in Vakkalainen 2019.

1st Editorial Decision 5th Feb 2026

Dear Dr. Lindqvist,

Thank you for the submission of your manuscript to EMBO reports and for your revision plan.

I agree that your proposed revisions are good and I would thus like to invite you to revise your manuscript with the understanding that the referee concerns must be fully addressed and their suggestions taken on board. Please address all referee concerns in a complete point-by-point response.

Acceptance of the manuscript will depend on a positive outcome of a second round of review. It is EMBO reports policy to allow a single round of major revision only and acceptance or rejection of the manuscript will therefore depend on the completeness of your responses included in the next, final version of the manuscript.

We realize that it is difficult to revise to a specific deadline. In the interest of protecting the conceptual advance provided by the work, we recommend a revision within 3 months (8th May 2026). Please discuss the revision progress ahead of this time with the editor if you require more time to complete the revisions.

You can either publish the study as a short report or as a full article. For short reports, the revised manuscript should not exceed 27,000 characters (including spaces but excluding materials & methods and references) and 5 main plus 5 expanded view figures. The results and discussion sections must further be combined, which will help to shorten the manuscript text by eliminating some redundancy that is inevitable when discussing the same experiments twice. For a normal article there are no length limitations, but it should have more than 5 main figures and the results and discussion sections must be separate. In both cases, the entire materials and methods must be included in the main manuscript file.

IMPORTANT NOTE: we perform an initial quality control of all revised manuscripts before re-review. Your manuscript will FAIL this control and the handling will be DELAYED if the following APPLIES:
1) A data availability section providing access to data deposited in public databases is missing. If you have not deposited any data, please add a sentence to the data availability section that explains that.
2) Your manuscript contains statistics and error bars based on n=2. Please use scatter blots in these cases. No statistics should be calculated if n=2.

When submitting your revised manuscript, please review the submission guidelines (https://link.springer.com/journal/44319/submission-guidelines#cms-Revised-submissions) and carefully review the instructions that follow below. Failure to include requested items will delay the evaluation of your revision.

1) a .docx formatted version of the manuscript text (including legends for main figures, EV figures and tables). Please make sure that the changes are highlighted to be clearly visible.

2) individual production quality figure files as .eps, .tif, .jpg (one file per figure). See hhttps://media.springernature.com/original/springer-cms/rest/v1/content/27825798/data/v1 for more info on how to prepare your figures.

3) We replaced Supplementary Information with Expanded View (EV) Figures and Tables that are collapsible/expandable online. A maximum of 5 EV Figures can be typeset. EV Figures should be cited as 'Figure EV1, Figure EV2" etc... in the text and their respective legends should be included in the main text after the legends of regular figures.

- For the figures that you do NOT wish to display as Expanded View figures, they should be bundled together with their legends in a single PDF file called *Appendix*, which should start with a short Table of Content. Appendix figures should be referred to in the main text as: "Appendix Figure S1, Appendix Figure S2" etc. See 'Expanded View' section of the submission guidelines.

- Additional Tables/Datasets should be labeled and referred to as Table EV1, Dataset EV1, etc. Legends have to be provided in a separate tab in case of .xls files. Alternatively, the legend can be supplied as a separate text file (README) and zipped together with the Table/Dataset file.

4) a .docx formatted letter INCLUDING the reviewers' reports and your detailed point-by-point responses to their comments. As part of the EMBO Press transparent editorial process, the point-by-point response is part of the Review Process File (RPF), which will be published alongside your paper.

5) a complete author checklist, which you can download from our author guidelines. Please insert information in the checklist that is also reflected in the manuscript. The completed author checklist will also be part of the RPF.

6) Please note that all corresponding authors are required to supply an ORCID ID for their name upon submission of a revised manuscript (<https://orcid.org/>), which can be linked to your profile in the manuscript submission system.

7) Before submitting your revision, primary datasets produced in this study need to be deposited in an appropriate public database. Please remember to provide a reviewer password if the datasets are not yet public. The accession numbers and database should be listed in a formal "Data Availability" section placed after Methods. Please note that the Data Availability Section is restricted to new primary data that are part of this study. * Note - All links should resolve to a page where the data can be accessed. *

If your study has not produced novel datasets, please mention this fact in the Data Availability Section.

8) At EMBO Press we ask authors to provide source data for the main manuscript figures. You will receive a separate email with instructions for providing source data with your revised manuscript, including how to upload and organize the files.

9) Our journal also encourages inclusion of *data citations in the reference list* to directly cite datasets that were re-used and obtained from public databases. Data citations in the article text are distinct from normal bibliographical citations and should directly link to the database records from which the data can be accessed. In the main text, data citations are formatted as follows: "Data ref: Smith et al, 2001" or "Data ref: NCBI Sequence Read Archive PRJNA342805, 2017". In the Reference list, data citations must be labeled with "[DATASET]". A data reference must provide the database name, accession number/identifiers and a resolvable link to the landing page from which the data can be accessed at the end of the reference.

10) Regarding data quantification, the following points must be specified in each figure legend:

- the name of the statistical test used to generate error bars and P values,

- the number (n) of independent experiments (please specify technical or biological replicates) underlying each data point,

- the nature of the bars and error bars (s.d., s.e.m.),

- If the data are obtained from n Program fragment delivered error ``Can't locate object method "less" via package "than" (perhaps you forgot to load "than"?) at //ejpvfs23/sites23b/embor_www/letters/embor_decision_revise_and_review.txt line 54.' 2, use scatter blots showing the individual data points.

Discussion of statistical methodology can be reported in the materials and methods section, but figure legends should contain a basic description of n, P and the test applied.

- Please also include scale bars in all microscopy images.

11) The journal requires a statement specifying whether or not authors have competing interests (defined as all potential or actual interests that could be perceived to influence the presentation or interpretation of an article). In case of competing interests, this must be specified in your disclosure statement.

12) All Materials and Methods need to be described in the main text using our 'Structured Methods' format, which is required for all research articles. According to this format, the Methods section includes a separate Reagents and Tools Table file (listing key reagents, experimental models, software and relevant equipment and including their sources and relevant identifiers) and a Methods and Protocols section describing the methods using a step-by-step protocol format. The aim is to facilitate adoption of the methodologies across labs. More information on how to adhere to this format as well as a downloadable template (.docx) for the Reagents and Tools Table can be found in our author guidelines.

We would also welcome the submission of cover suggestions, or motifs to be used by our Graphics Illustrator in designing a cover.

As part of the EMBO publication's Transparent Editorial Process, EMBO reports publishes online a Review Process File (RPF) to accompany accepted manuscripts. This File will be published in conjunction with your paper and will include the referee reports, your point-by-point response and all pertinent correspondence relating to the manuscript.

You are able to opt out of this by letting the editorial office know (emboreports@embo.org). If you do opt out, the Review Process File link will point to the following statement: "No Review Process File is available with this article, as the authors have chosen not to make the review process public in this case."

I look forward to seeing a revised form of your manuscript when it is ready.

Best regards,
Esther

Esther Schnapp, PhD
Senior Editor
EMBO reports

We thank the three reviewers for their time and valuable suggestions, which we believe have improved the manuscript. Please find our answers to the reviewers point-by-point below.

Changes to the manuscript are marked in red. New figures are:

Fig 4F:	*G2 checkpoint assessment in patient fibroblasts by flow cytometry*
Fig EV2:	*Workflow for cumulative microscopy, including three color acquisition and comparison to single round immunofluorescence.*
Fig EV4	*DNA damage foci and mitotic cells in patient fibroblasts*
Appendix Fig S2B, C	*Cumulative microscopy compared to single round immunofluorescence in fibroblasts*
Appendix Fig S3	*Cumulative microscopy after varying exposure times*
Appendix Fig S5	*Flow cytometry of patient cells*
Appendix Fig S7	*Gating strategy for flow cytometry*

Reviewer #1 (Evidence, reproducibility and clarity (Required)):

#Summary: The study introduces cumulative microscopy, a quantitative method to assess cell cycle and stress markers in individual primary fibroblasts obtained from patients with rare genetic disorders. This approach is compatible with neural network-based analysis, enabling the identification of distinct cellular states.

#Major Comments:
1. Clarification of Experimental Replicates
While the authors provide extensive single-cell data (over 96,000 cells from 15 individuals), it is not clearly stated how many times key experiments were independently repeated. Specifying the number of biological and technical replicates would improve confidence in the reproducibility and robustness of the findings.

We agree and apologize for this mistake. We have now included details on biological and technical replicates for all experiments.

2. Validation of Cumulative IF with Standard Controls
The authors demonstrate that cumulative microscopy can quantify protein levels by immunofluorescence (IF), but including a control experiment would strengthen confidence in these results. For example, performing IF on cells with no previous staining and comparing the results would help validate the method. This could be done using control versus disease

fibroblasts, or a cell line such as RPE under a cell cycle perturbation, like aphidicolin.

We agree that such a comparison can be valuable also on the perturbed states. In addition to the comparison between cumulative microscopy and IF on unperturbed RPE cells in fig 1, we now have included an experiment for patient fibroblasts (Appendix Figure S2B). We treated the cells with or without Aphidicolin and quantitatively compared the signal distribution (Appendix Figure S4C).

3.Clarification of Experimental Details and Quantification Methods
The manuscript notes that cells were seeded in a 96-well imaging plate overnight, but additional details would aid reproducibility. It would be helpful to clarify whether the wells were glass- or plastic-bottom, whether any surface treatment was applied, and how protein levels were quantified (e.g., using nuclear masks or another method).

We agree and have extended the description of experimental conditions.

4. Clarification of Figure Plot Types
The figures should specify the type of plots presented. For example, box plots could indicate median, interquartile range, and outliers, while violin plots could clarify whether the width represents data density and which summary statistics are included. Clear labelling would enhance interpretability and reproducibility (e.g., Figure 4).

We agree and now have added this information to figure legends.

#Minor Comments:
- The authors should acknowledge in their introduction the work developed by Stallaert and colleagues 2022 (DOI: 10.1016/j.cels.2021.10.007) that maps the dynamics of 48 key human cell cycle proteins using single-cell imaging and computational modelling, providing crucial insights into cell proliferation, arrest, and therapeutic strategies for diseases like cancer.

We agree and now cite this study in the introduction as well as in the results in relation to cell cycle exit.

- Supplementary Figure 6 is never mentioned in the text.

Previous supplementary figure 6 – now Appendix figure S4 – is described together with figures 4A and 4B

- In Supplementary Figure 1, the panels are labelled as "A - Imaging" and "C - Quantification." Should the second panel be labelled "B - Quantification" instead?

We have now corrected this mistake (currently Figure EV1).

- Two supplementary items (Supplementary Figure 5A and Supplementary Figure 8) are currently presented as figures but consist entirely of tabular data. It would be more appropriate

to present these as tables for clarity and proper formatting.

We agree and have now changed these figures into tables.

Reviewer #1 (Significance (Required)):

#General assessment
This study presents cumulative microscopy, an innovative method for quantitative single-cell analysis of cell cycle and stress markers in primary fibroblasts from patients with rare genetic disorders. Its strengths include high-resolution single-cell data, integration with neural network-based analysis, and the ability to quantify multiple proteins in the same experiment. Limitations include unclear reporting of experimental replicates, missing methodological details, and lack of validation controls, which, if addressed, would make the method more robust. Overall, resolving these issues would enhance reproducibility, clarity, and the impact of this promising technique.

#Advance
This study introduces cumulative microscopy, enabling multiplexed single-cell analysis of cell cycle and stress markers in the same experiment-an advance over previous approaches. Compared to related work, it applies this method to primary fibroblasts from patients with rare genetic disorders, providing new mechanistic and functional insights into cellular heterogeneity and stress responses. The study represents both a technical and conceptual advance, offering a framework for integrating high-content imaging with neural network-based analyses.

#Audience
This study will primarily appeal to a specialised audience in cell biology, single-cell analysis, and disease modelling, especially researchers studying rare genetic disorders, cell cycle regulation, and stress responses. Its combination of multiplexed imaging and neural network-based analysis may also interest those in computational biology and high-content imaging. Beyond this field, the method could be useful in translational research for understanding disease mechanisms or screening therapeutic interventions.

#My expertise
My expertise lies in cell cycle regulation, live cell imaging of single cells, quantitative microscopy, high-content imaging analysis, and molecular modelling. I am confident in evaluating the technical and conceptual aspects of this study, including experimental design, imaging methods, and data analysis.

Reviewer #2 (Evidence, reproducibility and clarity (Required)):

Summary
Akopyan et.al. introduces an imaging approach termed cumulative microscopy, in which the same biospecimen is subjected to sequential rounds of staining and imaging. Spatial information for each target protein is then computationally integrated through post-image

processing and analysis. The principal advantage claimed by the authors is that this strategy circumvents the need for fluorophore bleaching or antibody stripping between imaging cycles, a step usually required in multiplex microscopy. Using this technique, the authors report that a subset of fibroblasts derived from patients with genetic disorders exhibits replication stress, which may contribute to cancer progression. While the concept is potentially impactful, the study lacks sufficient validation at both the imaging and computational analysis levels. Rigorous methodological and biological validation is essential, and the current version of the manuscript requires substantial revision before it can be considered for publication elsewhere.

Major Comments

1. The manuscript does not present sufficient experimental validation of the proposed methodology. In the authors' workflow, repeated labeling is performed with the same secondary antibody sets. Conventional multiplex immunofluorescence is limited by antibody species compatibility and microscope channel capacity, typically requiring antibody quenching or stripping. In contrast, the authors rely on computational subtraction of prior labeling to permit repeated use of the same secondary antibody sets.

This approach raises several critical concerns:

(A) Although Figure 1 shows single-color images of dTCTP, Cyclin A2, and ATAD2, the key advantage of cumulative microscopy should lie in true multicolor labeling across cycles. If only three proteins are analyzed, conventional three-color immunofluorescence would suffice. The authors should demonstrate at least three cycles of labeling with three different targets per cycle, providing representative pre- and post-correction images, detailed processing workflows, and higher-resolution image data.

We agree that multi-colour labeling is beneficial to increase the number of targets. In new figure EV2, we now show a cumulative microscopy experiment using three different secondary antibodies. We added images for all nine primary antibodies pre and post correction and have increased the detail of the description of the workflow.

(B) The risk of non-specific binding and artifacts introduced by cumulative labeling has not been addressed. Without inactivation or removal of prior antibodies, cross-reactivity and increased background are likely, particularly when secondary antibodies bind to primary antibodies from subsequent cycles of the same species.

We respectfully disagree. The method inherently quantifies the change in background when secondary antibodies cross-react or bind to primary antibodies from previous cycles. This is in contrast to most existing methods, and we believe the aspect lifted by the reviewer is therefore an advantage of cumulative microscopy. We have now expanded the description of how the changing background is quantified in methods and the text in the Appendix.

(C) The performance of the method when identical fluorophores are reused across cycles with overlapping localization is unclear.

We respectfully disagree. In figure 1 we show that three targets with overlapping localisation can be separated. We assess this by comparison to single-round quantitative IF and to

expression during live-cell imaging of a fluorescently tagged protein. By choosing target proteins that show different expression dynamics during the cell cycle we can assess reliability of the separation of signals. We now expand the description of this in the text. Further, in new Figure EV2, we now include a quantification of the differences between single rounds of immunofluorescence and all steps of a cumulative microscopy experiment using 9 different primary antibodies.

(D) The subtraction strategy requires quantitative validation. The manuscript should present expected levels of under- or over-correction and assess the reliability of this computational step.

We agree and have now expanded this data. In figure 1D we indicate the expected levels of under- and over-correction. Figure EV3 shows how the calculations are performed and indicate statistics based on real data. In figure 1D and in Appendix figure S2 we compare the results to standard quantitative immunofluorescence and to live-cell imaging of a tagged protein and find similar results. We further show that the expected variation is low compared to cell-cell variation. In new Figure EV2, we now include a quantification of the differences between single rounds of immunofluorescence and all steps of a cumulative microscopy experiment using 9 different primary antibodies.

2. The manuscript should clarify the rationale for choosing ATAD2, Cyclin A2, and pTCTP in Figure 1B. Their specific biological roles and relevance to the experimental design need to be explicitly explained.

We agree and have extended this section in the revised manuscript. Briefly, they were selected as they show characteristic and distinct changes through the cell cycle. These distinct changes allow for assessment for the reliability of the method. For example, ATAD2 decreases through G2 phase, ending at very low signal, whereas pTCTP and Cyclin A2 in the previous imaging cycles increase throughout G2 (figure 1C). Thus, when ATAD2 is very low, the preceding stainings are very high. The selection of antibodies thus enables us to show that the signal in previous cycles does not contribute significantly to the signal in the following cycle.

3. According to the Methods, the authors used a 20× NA 0.45 air objective for all experiments. With such a low numerical aperture, it is doubtful that the authors could adequately resolve the detailed characteristics of the markers analyzed. The authors should provide representative raw and corrected images for each marker, along with detailed descriptions of their labeling and imaging strategies, to allow proper evaluation of the method.

We have substantially expanded the Methods and the text in the Appendix to provide a more detailed description of the experimental procedures, including the labeling strategy and calibration approach.

To directly address the concern regarding resolution, we now include representative examples demonstrating that DNA damage foci can be reliably resolved under our acquisition settings (Figure EV4). We also clarify in the main text that our analytical pipeline operates at the level of datasets derived from segmented nuclei, rather than relying on pixel-level quantification. This object-based approach of cellular measurements is less sensitive to small spatial inaccuracies across imaging cycles.

Importantly, although our downstream analysis is segmentation-based, pixel-wise calibration is possible. We now provide representative raw and corrected images for nine antibody stainings acquired through cumulative microscopy (three staining cycles with three targets per cycle) in Figure EV2. This allows a direct assessment of image quality and the effectiveness of the correction procedure.

4. It is unclear whether cumulative microscopy is applicable to a broad range of targets and whether it can be adapted for higher-resolution imaging, or if it remains constrained to relatively large effective pixel sizes as obtained with a 20× objective. The authors should provide results and performance tests to demonstrate the versatility and limitations of this method.

We agree, we do not know where the borders are in terms of high-resolution imaging. This is a relevant path, and we are initiating a project to test cumulative microscopy for various super-resolution techniques, but that will take time and we feel that a full test of the limits for resolution is outside of the scope of this manuscript. This manuscript validates the method for quantification of cellular signals (image cytometry), for which we believe it is a highly useful method. We have now introduced a section in the end of the main text in which we more clearly make this statement.

3. The manuscript lacks methodological details on how the same imaging location is reliably identified across repeated rounds of staining.

We agree that this information was lacking and have now introduced a section in materials entitled "Image registration and field-of-view alignment".

4. The claim that cumulative microscopy is quantitative is not adequately substantiated. Adjusting exposure times between imaging cycles compromises the assumption that signal intensity remains linearly proportional to protein abundance within the relevant cellular domains. The authors must clarify how quantitative accuracy is maintained under these conditions and provide comprehensive experimental validation.

We agree with the reviewer that changing exposure time may affect accuracy as changing exposure time may affect the signal-to-noise ratio within one antibody staining. Further, it may result in a lower dynamic range of the camera being used and the relative strength of the various signals may affect results. In new Appendix Figure S3, we now include a quantitative

comparison using an experiment with parallel acquisition and varying exposure conditions. We do not detect quantitative differences in the distribution of the resulting stainings.

Regarding linear proportionality, each antibody staining is acquired seperately and calculated seperately and the same acquisition time is importantly used within one antibody staining. The abundance is thus estimated independently per antibody staining. This is similar to a single round of quantitative IF with two colours – the quantification of each colour/channel is separate, and exposure conditions can be different between the two colours/channels.

5. The calibration procedure is insufficiently described. Key points to address include: How many targets and fluorophore combinations were tested? How was antibody variability (e.g., background, specificity) accounted for? Was calibration assessed on a per-pixel or per-cell basis, and is population-level evaluation adequate for local corrections? Which parameters can or cannot be corrected by the calibration process? Without this information, the conclusions drawn from calibration remain unconvincing.

We have increased the detail of the calibration method both in materials and as a new section in Appendix.

6. Figure S2 suggests that segmentation is critical, as pixel-by-pixel correlations deteriorate with successive staining rounds. The authors should define thresholds for acceptable error, quantify how errors propagate across cycles, and clarify how pixel assignments are maintained when subsequent cycles introduce new, bright cellular features that may obscure prior signals. Furthermore, it remains unclear whether this approach is limited to low-resolution, large-pixel images (e.g., 20×) or whether it is robust enough to perform reliably with high-resolution imaging (e.g., 100× objectives with ~60 nm effective pixel size).

We agree that segmentation is critical, as shown in the previous Figure S2, now Appendix Figure S1, but disagree with the reviewer that correlations deteriorate with successive staining rounds and that errors should propagate. First, segmentation is independently performed on all cycles. Second, each staining round uses an independent background, based on the antibodies present in the last staining round plus one round with secondary antibody only. Therefore, errors do not propagate through cycles, which is one of the advantages of cumulative microscopy.

We agree that if segmentation was performed on the cumulative antibody stainings, assigning the same area may be difficult. In this manuscript, we use segmentation of nuclei in a separate channel. In the method section, we now describe that segmentation needs to be performed on comparable structures across cycles.

For high resolution imaging, please see our answer to the first point 4 above. We now add a statement at the end of the main text that the method is not tested for high resolution imaging.

7. The prevalence and significance of CHH and MUL should be described more thoroughly in the Introduction. The rationale for selecting these two disorders as models must be clearly justified.

On p2 line 15 we now specify that these disorders are very rare and that they in addition to increased cancer risk share growth failure.

8. In Figures 1C and 1D, the cumulative IF data relative to mitotic timing should be labeled as estimated time, not as actual time measured by live-cell imaging. Presenting it otherwise is misleading and creates significant confusion.

We agree and thank the reviewer for spotting this mistake. We have reformulated the labeling.

9. The Methods section lacks information on the number of cells analyzed, which is essential for accurate quantification. Both the estimation of cell cycle progression and the determination of population frequencies require sufficiently large sample sizes to ensure reliability, yet such evaluations are missing. Does the method provide single-cell accuracy? What is the associated error as a function of sample size? How many cells are required for robust and accurate analysis?

We have now included information on number of cells. We have also reformulated the description of estimating time from fixed cells, and refer to our previous study in which we introduce the method and calculate estimated error as a function of population size (Akopyan et al, Mol Cell 2014)

10. For Figures 2B-C, it is not possible to evaluate accuracy without detailed description of how cell cycle stages were experimentally validated and how computational models were confirmed.

We have expanded the description of assessment of cell cycle stages and that they were used to confirm the classification accuracy of the model.

11. The choice of pTCTP, Cyclin A2, p21, γH2AX, Plk1, p53, and EdU in Figure 2 requires justification. Their expected temporal dynamics, localization patterns, and relevance to the study design must be described. The results should be interpreted beyond signal intensity, addressing whether cumulative microscopy accurately recapitulates known biological distributions.

We have expanded the description of the studied proteins and their expected dynamics. We have further expanded the description of how these are used to derive trajectories in Figure 3.

12. The claim that CHH cells exhibit replication stress must be quantitatively supported. The authors should measure γH2AX foci per cell. In addition, they must demonstrate that these foci

can indeed be visualized with a 20× air objective, typically, such structures are resolved only with higher-NA, high-magnification objectives. If not, the authors must validate how cumulative microscopy reliably detects these signals.

In new Figure EV4 we show images of γH2AX foci and a quantification of the number of foci in EdU positive cells.

12. The assertion that p53/p21 upregulation corresponds to G1 arrest or G2 exit is not adequately supported. Additional experiments are needed to distinguish cell cycle arrest from ongoing progression.

We agree that mere p53/p21 levels are not sufficient to distinguish arrest from ongoing progression. We have expanded the description of how the regions G2 exit and G1 stress in figure 4 are estimated. Briefly, G2 exit is characterized by 4n DNA content and an absence of Cyclin A2, whereas G1 stress is characterized by 2n DNA content, p53 and p21 expression, in addition to increased cell size. We have reformulated and clarified in the text that G1 stress does not necessarily mean that cell cycle progression is blocked and have expanded the description of cell cycle exit in G2.

13.To substantiate the claim that MUL and CHH cells activate a G2 checkpoint, the authors should perform flow cytometry as well as live-cell imaging with FUCCI or alternative markers to specifically assess G2 enrichment, in addition to immunofluorescence assays for checkpoint activation. Bright-field live imaging at 1-hour intervals is insufficient to support the authors' conclusion.

In new figure 4F, we now use flow cytometry to detect how inhibition of checkpoint kinases increases accumulation in mitosis. We find that fewer CHH and MUL cells accumulate in mitosis during unperturbed growth, but that the accumulation in mitosis is at least partially rescued by inhibition of checkpoint kinases. We would in addition like to point out that we refer in the text to our previous study (Vakkalainen et al 2019), in which we show a G2 delay in CHH cells using EdU pulse-chase.

14. The UMAP plots require clarification. Specifically: What datasets were combined? How were they integrated and validated? How are non-dividing populations (10-30% in CHH and MUL) represented in the plots? Do these cells correspond to G1, S, or G0? Do they lack γH2AX? The accuracy of the model must be evaluated against these populations.

We have added a new section in Methods, in which we describe UMAP construction in detail. The populations that at least temporally are not dividing are indicated in Figure 4D and E, the corresponding levels of γH2AX is indicated in Figure 3D. We also now refer to Stallaert et al 2022, which shows that cell exiting the cell cycle from G2 after Etoposide contain high γH2AX, whereas cells exiting or arresting in the following G1 phase contain lower γH2AX.

15. The effects of low-dose aphidicolin should be directly compared with those of standard concentrations to determine whether the observed results are simply due to partial polymerase

inhibition. If this is the case, all statements regarding aphidicolin must be interpreted with appropriate caution.

The low-dose Aphidicolin was chosen to prevent a full block of all DNA replication, as such a block is unlikely to occur in patient cells that can proliferate. We now included the statement "The low level was chosen to perturb but not completely block proliferation."

16. The proportion of G1, S, and G2 cells in each line (control, CHH, MUL) should be confirmed by an independent method, such as flow cytometry or alternative methods, to support the imaging data.

In new Appendix figure S5, we now show quantifications based on flow cytometry of cells from the 15 individuals used in Figures 2-4 and compare to the previous measurements by immunofluorescence. We assess cell cycle distribution by monitoring EdU incorporation and DNA content.

17. Differences in antibody staining efficiency and protein expression between cell types must be taken into account. Proper optimization and validation, along with assessment of target protein and RNA levels, are required to ensure fair and reliable comparisons.

We would like to emphasize that we in this manuscript only directly compare the same cell types. We also believe that assessing RNA levels here would be outside of the scope for this manuscript, and that RNA measurements are not required for conclusions based on protein staining. RNA levels for CHH cells were assessed in Vakkalainen 2019.

18. The reported classification accuracies (31-41% for CHH and MUL; ~50% phase assignment) are too low to be scientifically meaningful. The authors should critically evaluate whether such performance levels can support their conclusions.

We respectfully disagree. The classifications in figure 2 are a starting point for the further studies, with the detailed analysis in figures 3 and 4. Further, they fit well with our finding that not all patient cells show replication stress. Rather, it would have been surprising if all CHH and MUL cells from different individuals would have been identified solely based on expression of cell cycle and stress markers.

Minor comments:
1. Both results, Figures and methods section require substantial rewriting to include details.

We have increased the level of detail in these sections.

Reviewer #2 (Significance (Required)):

The principal significance of this study lies in the development of a method for multiplex immunofluorescence microscopy that avoids fluorophore bleaching and antibody stripping between successive labeling and imaging cycles. By circumventing these labor-intensive

processes, the approach offers a substantial advantage to researchers by reducing overall experimental time. However, the study is limited by insufficient performance evaluation and a lack of comprehensive data to assess the robustness, capacity, limitations, and scalability of the method. Addressing these gaps will be essential to enhance its accessibility and acceptance across diverse research fields.

Reviewer #3 (Evidence, reproducibility and clarity (Required)):

Summary:
Akopyan et al have developed cumulative microscopy to analyze repeated rounds of immunofluorescent stainings without the need of quenching, stripping or using secondary antibodies from different animals. They demonstrate the power of the system by performing cell cycle analyses in individual primary fibroblasts derived from patients. Using this approach, they showed different clustering of cells from patients with two different disorders. The authors conclude that multiplex microscopy is a powerful, yet straightforward method for multiplex immunofluorescence, applicable in a wide range of assays.

Major comments:
1) The authors address several potential pitfalls, such as slight movement of the samples from one imaging cycle to the next, dealing with bleaching during repeated imaging cycles, technical variability, changing exposure times. This thorough consideration and testing make the results very convincing.

2) The methods used are well described and statistical analyses are adequate. It is not clear to me whether the scripts are made publicly available; if yes, a link would be appreciated by all users.

We agree with the reviewer. Scripts are available at https://github.com/KAkopyan/Cumulative-Microscopy-Python

3) It would be interesting to identify mitotic cells, for example based on the P-TCPT staining combined with DAPI (if possible from the existing data; I am not suggesting redoing analyses using other mitotic markers). Given that MUL cells contain mutations in TRIM37, they might be identifiable based on mitotic delays and errors, which would also be consistent with the increased p53 and p21 staining.

We agree and have added a quantification of areas showing mitotic cells in new figure EV4. We indeed find an enrichment of MUL cells in mitosis.

Minor comments:
1) The figures are very clear.
2) I don't think the accumulation of the MUL cells in the UMAP (referred to on p 6 line 5) is shown.

We thank the reviewer for spotting this error – we now refer to the correct figure panels (Figure 4 D and E) for this point.

3) I believe the text would benefit from some more detailed explanations, depending on the target audience. In theory the method can be used for just about any cellular process, meaning not all interested readers will be experts in the cell cycle. Specifically, it would be useful to explain the choice of targets the authors stain for and why they are appropriate markers for cell cycle studies. It is not clear what the authors mean by "stress markers".

We agree and have now expanded the description of the targets in the main text.

4) The same applies for the trajectories shown on Figure 3 - how were these defined?

We agree and have now expanded the description of the trajectories in the main text.

5) The term "replication stress score" is not explained. Please describe the key points in the main text, or refer the reader to the detailed description in the Methods.

We now refer the reader to the description in the Methods section.

Reviewer #3 (Significance (Required)):

Significance
- The nature of the advance is mainly technical, this is an approach that has the potential to lead to many conceptual advances.
- The major strength of the approach is that it does not requires very specialized equipment and thus it can be used in most laboratories.
- The authors did a very good job of introducing existing methods and papers presenting previous studies. It could be relevant to also discuss spectral flowcytometry and microscopy as alternative methods for multiplexing (maybe pointing out pros and contras).
- I think very many researchers would be interested in the reported approach, since the method is applicable for any biological question where immunofluorescence can be employed.
- My expertise: I have a solid background in the cell-cycle field and consider myself reasonably well-versed in immunofluorescence and microscopy. However, I am no expert in large-scale data analyses.

1st Revision - Editorial Decision 10th Jun 2026

Dear Dr. Lindqvist,

Thank you for the submission of your revised manuscript to EMBO reports. We have now received the comments from previous referee 2, who was asked to assess it.

While this referee overall supports the publication of your ms, s/he also points out that many details are missing, and especially given that this study is a methods paper, this information is very important. Can you please address all referee comments and also include a point-by-point response with your final ms.

A few editorial requests will also need to be addressed:

- Please correct the conflict of interest subheading to "Disclosure and Competing Interests Statement"

- The author credits need to be removed from the ms file. All credits need to be entered during online ms submission.

- The REFERENCE format needs to be corrected: et al needs to be used after 10 author names; DOIs should only be used for preprints and datasets that have not been published yet. Please correct to the EMBO reports reference style.

- The separate "Funding" section heading is not needed in the ms as funding information goes under Acknowledgments.

- For Table EV1 and Table EV2 it would be better to provide the legend of each table on the same sheet with the table so that the PDF conversions look better.

- The text in the APPENDIX FILE should ideally be moved to the main ms file. We cannot offer "supplemental text". You can have methods in the Appendix file, but I am not sure whether the extra text is only about methods. "Supplemental" should not be used as nomenclature. The Appendix file needs a title page with the ms title and a table of content with page numbers; each figure legend should directly follow its figure. Please correct.

- Please upload the Reagents & Tools table as a separate file and remove it from the ms file.

- Materials and Methods should be just Methods

- The manuscript sections should be in the following order: Title page - Abstract - Introduction - Results - Discussion - Methods - Data Availability - Acknowledgments - Disclosure Statement & Competing Interests - References - Figure Legends - (Main Tables with legends if applicable) - Expanded View Figure Legends.

- Our routine image analysis detected a possible cell reuse within Figure EV4D - two cells appear very similar with high pixel similarity. Can you please clarify?

- Please write the abstract in present tense, as per journal policy.

* Figure Legends - Comments *

- Please indicate the statistical test used for data analysis in the legends of figures 4D, E
- Please note that information related to n is missing in the legends of figures 4B, C, D, E

EMBO press papers are accompanied online by A) a short (1-2 sentences) summary of the findings and their significance, B) 2-3 bullet points highlighting key results and C) a synopsis image. The synopsis image that you sent is good, but please send us the short summary and bullet points along with the final manuscript.

I look forward to seeing a final version of your manuscript when it is ready.

Best regards,
Esther

Esther Schnapp, PhD
Senior Editor
EMBO reports

Referee #1:

Akopyan et al. describe a multiplex immunofluorescence microscopy approach, termed cumulative microscopy, and use this platform to generate imaging datasets for neural-network-based analysis. As a proof of concept, the authors report that cartilage-hair hypoplasia (CHH) patient cells show evidence of replication stress, whereas Mulibrey nanism patient cells do not show the same phenotype.

The revised manuscript includes additional validation experiments and more detailed datasets, which strengthen the study. In my view, cumulative microscopy is an impressive and potentially useful method with broad applicability across diverse research fields. However, because the manuscript is primarily positioned as a methodological study, the current version would benefit from substantially more detailed methodological guidance to enable reproducibility and adoption by readers.

My main concern is that, in its current form, it may still be difficult for readers to reproduce or apply cumulative microscopy in their own experimental systems. For a methods-focused manuscript, the authors should provide a clearer step-by-step workflow, including the experimental protocol, imaging requirements, computational pipeline, quality-control criteria, and limitations.

Cumulative microscopy:

First, the immunofluorescence protocol requires more detail. Although the authors cite previous work, the staining procedure is central to cumulative microscopy and should be described directly in this manuscript. The authors should specify fixation conditions, whether the method is compatible with common other fixation approaches such as methanol or acetone, and, if not, why. They should also provide incubation times, temperatures, washing conditions, storage conditions between sequential staining and imaging rounds, and how long samples can be stored without compromising signal quality or reproducibility.

Second, the imaging-system requirements remain unclear. Most images appear to have been acquired using a 20× air objective on a confocal microscope. The authors should clarify whether this setup is required or whether the method is compatible with other objectives, magnifications, and imaging platforms. If 20× air-objective confocal imaging is required, the authors should explain why. If higher magnifications or other microscope systems are compatible, supporting data or practical guidance should be provided.

Third, the manuscript should more clearly describe how the same field of view, cell population, or individual cells are repeatedly imaged across staining cycles. Although the authors provide image-registration analysis for field-of-view alignment, they do not clearly explain how the same fields are relocated during microscopy. The authors should specify whether this requires particular hardware, software, stage-positioning accuracy, cell density, field size, or other experimental conditions.

Fourth, the computational workflow for image processing should be better documented. The authors should provide information on required computer specifications, image formats, processing time for each step, software parameters, error handling, and quality-control criteria. A complete workflow describing how raw images are converted into cumulative microscopy outputs would substantially improve reproducibility and accessibility.

Neural-network:

The neural-network analysis also requires additional explanation. The authors should provide more detail on model architecture, training strategy, training dataset size, validation approach, and performance metrics. In particular, the manuscript should clarify how many cells are required for training and which image features most strongly contribute to classification accuracy.

The precision analysis in Figure 2D is also unclear. The authors should specify whether the model was evaluated by classifying individual unseen cells as G1 or non-G1, or by classifying mixed populations containing all cell-cycle phases. The latter would be more informative. If mixed populations were used, the authors should report the fraction of unclassified cells and estimate how many cells were misclassified or incorrectly excluded, such as cells expected to be in G1 but left unclassified.

Finally, the generalizability of the neural-network model should be clarified. The model was trained using control primary fibroblasts and then applied to CHH and Mulibrey nanism patient cells, where substantial classification errors were observed. The authors should explain how broadly the model generalizes to other primary fibroblast lines or cell types, and whether differences in antibody penetration, staining intensity, background signal, or marker expression across cell lines contribute to classification errors. Quantitative comparisons of staining quality and marker intensity across all lines would strengthen the conclusions. For Figure 2E, marker-level analysis should be used to identify which features drive misclassification in CHH and Mulibrey nanism cells, followed by direct validation of the staining results.

Although authors suggested the immunofluorescence-based assay appears broadly consistent with flow cytometry, the estimated S-phase fractions differ substantially. Flow cytometry shows more than 20% S-phase cells across cell types in Figure S5, whereas the immunofluorescence-based assay shows approximately 10%. The authors should explain this discrepancy and clarify which method is more accurate.

Referee #1:

Akopyan et al. describe a multiplex immunofluorescence microscopy approach, termed cumulative microscopy, and use this platform to generate imaging datasets for neural-network-based analysis. As a proof of concept, the authors report that cartilage-hair hypoplasia (CHH) patient cells show evidence of replication stress, whereas Mulibrey nanism patient cells do not show the same phenotype.
The revised manuscript includes additional validation experiments and more detailed datasets, which strengthen the study. In my view, cumulative microscopy is an impressive and potentially useful method with broad applicability across diverse research fields. However, because the manuscript is primarily positioned as a methodological study, the current version would benefit from substantially more detailed methodological guidance to enable reproducibility and adoption by readers.
My main concern is that, in its current form, it may still be difficult for readers to reproduce or apply cumulative microscopy in their own experimental systems. For a methods-focused manuscript, the authors should provide a clearer step-by-step workflow, including the experimental protocol, imaging requirements, computational pipeline, quality-control criteria, and limitations.

We thank the reviewer for the comments and suggestions. We have now added a step-by-step description of a cumulative microscopy workflow, including experimental protocol, imaging requirements, computational pipeline, quality-control criteria, and limitations.

Cumulative microscopy:
First, the immunofluorescence protocol requires more detail. Although the authors cite previous work, the staining procedure is central to cumulative microscopy and should be described directly in this manuscript. The authors should specify fixation conditions, whether the method is compatible with common other fixation approaches such as methanol or acetone, and, if not, why. They should also provide incubation times, temperatures, washing conditions, storage conditions between sequential staining and imaging rounds, and how long samples can be stored without compromising signal quality or reproducibility.

We have now added a step-by-step description of a cumulative microscopy workflow. Fixation and staining conditions are now explicitly described, including fixation (3.7% formaldehyde, 5 min), permeabilization (ice-cold methanol, 2 min), blocking conditions, antibody incubation times, and washing steps.
We clarified that the protocol reflects a widely used immunofluorescence workflow optimized for adherent cells and commercially available antibodies. Importantly, the method is not restricted to a single fixation approach, and compatibility with alternative fixation strategies (e.g., methanol or acetone) is primarily determined by antibody performance and cell-type-specific requirements, as is standard in immunofluorescence experiments.
We added a detailed description of the sequential staining and imaging workflow, including handling between cycles, incubation conditions, and reuse of positional imaging coordinates.

Storage conditions between staining and imaging steps are now specified (samples maintained in TBS in multiwell plates), and practical guidance for handling between cycles is included.

Second, the imaging-system requirements remain unclear. Most images appear to have been acquired using a 20× air objective on a confocal microscope. The authors should clarify whether this setup is required or whether the method is compatible with other objectives, magnifications, and imaging platforms. If 20× air-objective confocal imaging is required, the authors should explain why. If higher magnifications or other microscope systems are compatible, supporting data or practical guidance should be provided.

We clarified that imaging was performed using a widefield fluorescence microscope with a 20× air objective (PLAN). This configuration provides a balance between resolution and throughput (number of cells per field) and is suitable for assessing cellular states.
We state that the method is not limited to this specific configuration and can be implemented on other fluorescence microscope platforms, provided that signal-to-noise and dynamic range are appropriately optimized.

Third, the manuscript should more clearly describe how the same field of view, cell population, or individual cells are repeatedly imaged across staining cycles. Although the authors provide image-registration analysis for field-of-view alignment, they do not clearly explain how the same fields are relocated during microscopy. The authors should specify whether this requires particular hardware, software, stage-positioning accuracy, cell density, field size, or other experimental conditions.
Fourth, the computational workflow for image processing should be better documented. The authors should provide information on required computer specifications, image formats, processing time for each step, software parameters, error handling, and quality-control criteria. A complete workflow describing how raw images are converted into cumulative microscopy outputs would substantially improve reproducibility and accessibility.

We clarified that imaging is performed using a microscope equipped with an automated stage, which allow reuse of positional imaging coordinates for all imaging cycles.
To account for minor positional inaccuracies of the automated stage, we implemented a post-acquisition image registration step, which corrects for small lateral shifts between cycles. A script for performing this step is provided and described in the step-by-step description. We now explicitly state that this approach does not require specialized hardware beyond a standard automated stage, although repositioning accuracy depends on stage precision.
We also clarified that optimal imaging conditions follow standard immunofluorescence practices, including moderate cell density (~50–70% confluency) to facilitate reliable segmentation and tracking of the same cell populations.

We have added a detailed description of the image-processing pipeline, including input formats, alignment, and generation of cumulative microscopy outputs.

Briefly, raw fluorescence images acquired across staining cycles are first aligned using image-registration algorithms to correct for stage drift. After alignment, images are processed to ensure consistent signal scaling and quality, followed by integration across cycles to generate cumulative datasets.
We also now specify practical considerations, including file formats, computational requirements (standard workstations), and quality control criteria such as exclusion of saturated or low-signal images.

Neural-network:
The neural-network analysis also requires additional explanation. The authors should provide more detail on model architecture, training strategy, training dataset size, validation approach, and performance metrics. In particular, the manuscript should clarify how many cells are required for training and which image features most strongly contribute to classification accuracy.

In the method section we provide a comprehensive description of the:
model architecture, which consists of a feedforward neural network with three fully connected hidden layers and ReLU activation functions strategy, including feature standardization, the use of the Adam optimizer, and training over 600 epochs.
We also describe the validation approach, in which the dataset was split into training and test subsets (80:20) using a fixed random seed to ensure reproducibility, and model performance was evaluated on the held-out test set.

The training dataset size is now explicitly stated as 80% of the 96 000 cells. We also now clarify that input features are the fluorescence intensity of cell cycle and cellular stress stainings outlined in Appendix figure S2D, as well as nuclear area.

The precision analysis in Figure 2D is also unclear. The authors should specify whether the model was evaluated by classifying individual unseen cells as G1 or non-G1, or by classifying mixed populations containing all cell-cycle phases. The latter would be more informative. If mixed populations were used, the authors should report the fraction of unclassified cells and estimate how many cells were misclassified or incorrectly excluded, such as cells expected to be in G1 but left unclassified.

In Figure 2D: The model was trained on individual cells derived from heterogeneous populations containing all cell-cycle phases, enabling it to learn classification patterns under realistic conditions.

For evaluation, we used four independent datasets, as shown in Figure 2D. These include:

1. A mixed population containing all cell-cycle phases
2. A subset consisting only of G1-phase cells
3. A subset consisting only of S-phase cells
4. A subset consisting only of G2-phase cells

This design allowed us to directly assess whether restricting the evaluation dataset to specific cell-cycle phases improves the model's ability to distinguish between different cell types (e.g., control, CHH, and Mulibrey cells), compared to classification within heterogeneous populations. We now have expanded the description of this in the figure legend.

Finally, the generalizability of the neural-network model should be clarified. The model was trained using control primary fibroblasts and then applied to CHH and Mulibrey nanism patient cells, where substantial classification errors were observed. The authors should explain how broadly the model generalizes to other primary fibroblast lines or cell types, and whether differences in antibody penetration, staining intensity, background signal, or marker expression across cell lines contribute to classification errors. Quantitative comparisons of staining quality and marker intensity across all lines would strengthen the conclusions. For Figure 2E, marker-level analysis should be used to identify which features drive misclassification in CHH and Mulibrey nanism cells, followed by direct validation of the staining results.

We agree that the development of a generalized model capable of broadly classifying and recognizing different primary fibroblast lines based on marker signal patterns would be valuable. However, achieving such generalizability would require extensive additional work, including the systematic analysis of multiple primary cell lines and a wide range of antibody panels. We argue that this is beyond the scope of the current study.
Importantly, the primary goal of the relatively simple model presented here was not to establish a broadly generalizable classifier, but rather to provide a practical and interpretable approach to assess whether measurable differences exist between control, CHH, and Mulibrey nanism fibroblasts using a limited set of markers.
Specifically, the model was designed to determine, in a straightforward manner, whether these differences can be detected and whether they are associated with cell-cycle–related features, given the selected staining panel. Thus, the model serves primarily as an exploratory tool to identify and interpret biologically meaningful differences between conditions, rather than as a universal classification framework.

Although authors suggested the immunofluorescence-based assay appears broadly consistent with flow cytometry, the estimated S-phase fractions differ substantially. Flow cytometry shows more than 20% S-phase cells across cell types in Figure S5, whereas the immunofluorescence-based assay shows approximately 10%. The authors should explain this discrepancy and clarify which method is more accurate.

We agree that the estimated S-phase fractions differ between the two approaches although it is also important to note that a certain degree of variability is commonly observed between independent experiments even when using the same method. Measured cell-cycle distributions can be influenced by experimental conditions such as cell confluency, culture conditions, and timing of labeling. In the current situation, there is a large difference in time between when the experiments were performed. On the other hand, there are

methodological differences between the two techniques. These include using different culturing conditions (96-well imaging plate versus larger dish).

Further, flow cytometry provides highly sensitive, population-level measurements, detecting DNA synthesis signals across a large number of cells with minimal exclusion criteria. In contrast, the immunofluorescence-based approach relies on image segmentation and single-cell analysis, which introduces additional constraints. In particular, cells with low EdU signal, ambiguous staining, or suboptimal segmentation quality may be excluded or not confidently classified as S-phase cells.

As a result, the imaging-based method may apply more conservative classification criteria, which possibly leads to an underestimation of the S-phase fraction compared to flow cytometry. Additionally, differences in signal detection thresholds and background correction between the two methods may further contribute to this discrepancy.

We therefore consider these approaches to be complementary rather than directly equivalent. Flow cytometry provides a sensitive global estimate of S-phase fraction, whereas the immunofluorescence-based method can enable spatially resolved, single-cell analysis with stricter quality control.

Dr. Arne Lindqvist
Karolinska Institutet
Cell and Molecular Biology
Nobels vag 9
Biomedicum A7
Stockholm 171 77
Sweden

Dear Arne,

I am very pleased to accept your manuscript for publication in the next available issue of EMBO reports. Thank you for your contribution to our journal.

Your manuscript will be processed for publication by EMBO Press. It will be copy edited and you will receive page proofs prior to publication. Please note that you will be contacted by Springer Nature Author Services to complete licensing and payment information.

You may qualify for financial assistance for your publication charges - either via a Springer Nature fully open access agreement or an EMBO initiative. Check your eligibility: https://link.springer.com/journal/44319/how-to-publish-with-us

Should you be planning a Press Release on your article, please notify the production team via this form in order to coordinate publication and release dates: https://forms.cloud.microsoft/e/eH4A8JVP6g

If you have any questions, please do not hesitate to contact the Editorial Office. Thank you for your contribution to EMBO Reports.

Best,
Esther

Esther Schnapp, PhD
Senior Editor
EMBO reports

>>> Please note that it is EMBO Reports policy for the transcript of the editorial process (containing referee reports and your response letter) to be published as an online supplement to each paper. If you do NOT want this, you will need to inform the Editorial Office via email immediately. More information is available here: https://link.springer.com/partners/embo-press/editorial-policies#Peer%20review

EMBO Press Author Checklist

Corresponding Author Name: Arne Lindqvist	
Journal Submitted to: EMBO REPORTS	
Manuscript Number: EMBOR-2026-63732V1-T	

USEFUL LINKS FOR COMPLETING THIS FORM
The EMBO Journal - Author Guidelines
EMBO Reports - Author Guidelines
Molecular Systems Biology - Author Guidelines
EMBO Molecular Medicine - Author Guidelines

Reporting Checklist for Life Science Articles (updated January
This checklist is adapted from Materials Design Analysis Reporting (MDAR) Checklist for Authors. MDAR establishes a minimum set of requirements in transparent reporting in the life sciences (see Statement of Task: 10.31222/osf.io/9sm4x). Please follow the journal's guidelines in preparing your
Please note that a copy of this checklist will be published alongside your article.

Abridged guidelines for figures
1. Data
The data shown in figures should satisfy the following conditions:
- the data were obtained and processed according to the field's best practice and are presented to reflect the results of the experiments in an accurate and unbiased manner.
- ideally, figure panels should include only measurements that are directly comparable to each other and obtained with the same assay.
- plots include clearly labeled error bars for independent experiments and sample sizes. Unless justified, error bars should not be shown for technical
- if n<5, the individual data points from each experiment should be plotted. Any statistical test employed should be justified.
- Source Data should be included to report the data underlying figures according to the guidelines set out in the authorship guidelines on Data

2. Captions
Each figure caption should contain the following information, for each panel where they are relevant:
- a specification of the experimental system investigated (eg cell line, species name).
- the assay(s) and method(s) used to carry out the reported observations and measurements.
- an explicit mention of the biological and chemical entity(ies) that are being measured.
- an explicit mention of the biological and chemical entity(ies) that are altered/varied/perturbed in a controlled manner.
- the exact sample size (n) for each experimental group/condition, given as a number, not a range;
- a description of the sample collection allowing the reader to understand whether the samples represent technical or biological replicates (including how many animals, litters, cultures, etc.).
- a statement of how many times the experiment shown was independently replicated in the laboratory.
- definitions of statistical methods and measures:
  - common tests, such as t-test (please specify whether paired vs. unpaired), simple χ2 tests, Wilcoxon and Mann-Whitney tests, can be unambiguously identified by name only, but more complex techniques should be described in the methods section;
  - are tests one-sided or two-sided?
  - are there adjustments for multiple comparisons?
  - exact statistical test results, e.g., P values = x but not P values < x;
  - definition of 'center values' as median or average;
  - definition of error bars as s.d. or s.e.m.

Please complete ALL of the questions below.
Select "Not Applicable" only when the requested information is not relevant for your study.

Materials

Newly Created Materials	Information included in the manuscript?	In which section is the information available? (Reagents and Tools Table, Materials and Methods, Figures, Data Availability Section)
New materials and reagents need to be available; do any restrictions apply?	Yes	Data availability

Antibodies	Information included in the manuscript?	In which section is the information available? (Reagents and Tools Table, Materials and Methods, Figures, Data Availability Section)
For **antibodies** provide the following information:<br>- Commercial antibodies: RRID (if possible) or supplier name, catalogue number and or/clone number<br>- Non-commercial: RRID or citation	Yes	Reagents and tools table

DNA and RNA sequences	Information included in the manuscript?	In which section is the information available? (Reagents and Tools Table, Materials and Methods, Figures, Data Availability Section)
**Short novel DNA or RNA including primers, probes**: provide the sequences.	Not Applicable	

Cell materials	Information included in the manuscript?	In which section is the information available? (Reagents and Tools Table, Materials and Methods, Figures, Data Availability Section)
**Cell lines:** Provide species information, strain. Provide accession number in repository **OR** supplier name, catalog number, clone number, and/**OR** RRID.	Yes	Materials and methods
**Primary cultures:** Provide species, strain, sex of origin, genetic modification status.	Yes	Materials and methods
Report if the cell lines were recently **authenticated** (e.g., by STR profiling) and tested for mycoplasma contamination.	Yes	Materials and methods

Experimental animals	Information included in the manuscript?	In which section is the information available? (Reagents and Tools Table, Materials and Methods, Figures, Data Availability Section)
**Laboratory animals or Model organisms:** Provide species, strain, sex, age, genetic modification status. Provide accession number in repository **OR** supplier name, catalog number, clone number, **OR** RRID.	Not Applicable	
**Animal observed in or captured from the field:** Provide species, sex, and age where possible.	Not Applicable	
Please detail **housing and husbandry conditions**.	Not Applicable	

Plants and microbes	Information included in the manuscript?	In which section is the information available? (Reagents and Tools Table, Materials and Methods, Figures, Data Availability Section)
**Plants:** provide species and strain, ecotype and cultivar where relevant, unique accession number if available, and source (including location for collected wild specimens).	Not Applicable	
**Microbes:** provide species and strain, unique accession number if available, and source.	Not Applicable	

Human research participants	Information included in the manuscript?	In which section is the information available? (Reagents and Tools Table, Materials and Methods, Figures, Data Availability Section)
If collected and within the bounds of privacy constraints report on age, sex and gender or ethnicity for all study participants.	Not Applicable	

Core facilities	Information included in the manuscript?	In which section is the information available? (Reagents and Tools Table, Materials and Methods, Figures, Data Availability Section)
If your work benefited from core facilities, was their service mentioned in the acknowledgments section?	Yes	Acknowledgements

Design

**Study protocol**	**Information included in the manuscript?**	**In which section is the information available?** (Reagents and Tools Table, Materials and Methods, Figures, Data Availability Section)
If study protocol has been **pre-registered, provide DOI in the manuscript**. For clinical trials, provide the trial registration number **OR** cite DOI.	Not Applicable	
Report the **clinical trial registration number** (at ClinicalTrials.gov or equivalent), where applicable.	Not Applicable	

**Laboratory protocol**	**Information included in the manuscript?**	**In which section is the information available?** (Reagents and Tools Table, Materials and Methods, Figures, Data Availability Section)
Provide DOI OR other citation details if **external detailed step-by-step protocols** are available.	Not Applicable	

**Experimental study design and statistics**	**Information included in the manuscript?**	**In which section is the information available?** (Reagents and Tools Table, Materials and Methods, Figures, Data Availability Section)
Include a statement about **sample size** estimate even if no statistical methods were used.	Yes	Figure legend
Were any steps taken to minimize the effects of subjective bias when allocating animals/samples to treatment (e.g. **randomization procedure**)? If yes, have they been described?	Not Applicable	
Include a statement about **blinding** even if no blinding was done.	Not Applicable	
Describe **inclusion/exclusion criteria** if samples or animals were excluded from the analysis. Were the criteria pre-established?<br><br>If sample or data points were omitted from analysis, report if this was due to attrition or intentional exclusion and provide justification.	Not Applicable	
For every figure, are **statistical tests** justified as appropriate? Do the data meet the assumptions of the tests (e.g., normal distribution)? Describe any methods used to assess it. Is there an estimate of variation within each group of data? Is the variance similar between the groups that are being statistically compared?	Yes	Materials and methods

**Sample definition and in-laboratory replication**	**Information included in the manuscript?**	**In which section is the information available?** (Reagents and Tools Table, Materials and Methods, Figures, Data Availability Section)
In the figure legends: state number of times the experiment was **replicated** in laboratory.	Yes	
In the figure legends: define whether data describe **technical or biological replicates**.	Yes	

Ethics

**Ethics**	**Information included in the manuscript?**	**In which section is the information available?** (Reagents and Tools Table, Materials and Methods, Figures, Data Availability Section)
Studies involving **human participants**: State details of **authority granting ethics approval** (IRB or equivalent committee(s), provide reference number for approval.	Yes	materials and methods
Studies involving **human participants**: Include a statement confirming that **informed consent** was obtained from all subjects and that the experiments conformed to the principles set out in the WMA Declaration of Helsinki and the Department of Health and Human Services Belmont Report.	Yes	materials and methods
Studies involving **human participants:** For publication of **patient photos**, include a statement confirming that consent to publish was obtained.	Not Applicable	
Studies involving experimental **animals**: State details of **authority granting ethics approval** (IRB or equivalent committee(s), provide reference number for approval. Include a statement of compliance with ethical regulations.	Not Applicable	
Studies involving **specimen and field samples:** State if relevant **permits** obtained, provide details of authority approving study; if none were required, explain why.	Not Applicable	

**Dual Use Research of Concern (DURC)**	**Information included in the manuscript?**	**In which section is the information available?** (Reagents and Tools Table, Materials and Methods, Figures, Data Availability Section)
Could your study fall under dual use research restrictions? Please check biosecurity documents and list of **select agents and toxins** (CDC): https://www.selectagents.gov/sat/list.htm	Not Applicable	
If you used a select agent, is the security level of the lab appropriate and reported in the manuscript?	Not Applicable	
If a study is subject to dual use research of concern regulations, is the name of the **authority granting approval and reference number** for the regulatory approval provided in the manuscript?	Not Applicable	

Reporting

The MDAR framework recommends adoption of discipline-specific guidelines, established and endorsed through community initiatives. Journals have their own policy about requiring specific guidelines and recommendations to complement MDAR.

**Adherence to community standards**	**Information included in the manuscript?**	**In which section is the information available?** (Reagents and Tools Table, Materials and Methods, Figures, Data Availability Section)
State if relevant guidelines or checklists (e.g., **ICMJE, MIBBI, ARRIVE, PRISMA**) have been followed or provided.	Not Applicable	
For **tumor marker prognostic studies,** we recommend that you follow the **REMARK** reporting guidelines (see link list at top right). See author guidelines, under 'Reporting Guidelines'. Please confirm you have followed these guidelines.	Not Applicable	
For **phase II and III randomized controlled trials**, please refer to the **CONSORT** flow diagram (see link list at top right) and submit the CONSORT checklist (see link list at top right) with your submission. See author guidelines, under 'Reporting Guidelines'. Please confirm you have submitted this list.	Not Applicable	

Data Availability

**Data availability**	**Information included in the manuscript?**	**In which section is the information available?** (Reagents and Tools Table, Materials and Methods, Figures, Data Availability Section)
Have **primary datasets** been deposited according to the journal's guidelines (see 'Data Deposition' section) and the respective accession numbers provided in the Data Availability Section?	Yes	Data availability section
Were **human clinical and genomic datasets** deposited in a public access-controlled repository in accordance to ethical obligations to the patients and to the applicable consent agreement?	Not Applicable	
Are **computational models** that are central and integral to a study available without restrictions in a machine-readable form? Were the relevant accession numbers or links provided?	Not Applicable	
If publicly available data were reused, provide the respective **data citations in the reference list**.	Yes	
