# Supplementary material for: Cumulative microscopy reveals cellular states in fibroblasts from patients with genetic disorders
Source: EMBO Rep. 2026 Jul 30;27(17):5022–38. doi: 10.1038/s44319-026-00859-5 (PMC13554152; doi:10.1038/s44319-026-00859-5)
Supplement: Supplementary file 3 [file 44319_2026_859_MOESM9_ESM.pdf]

Expanded View Figures

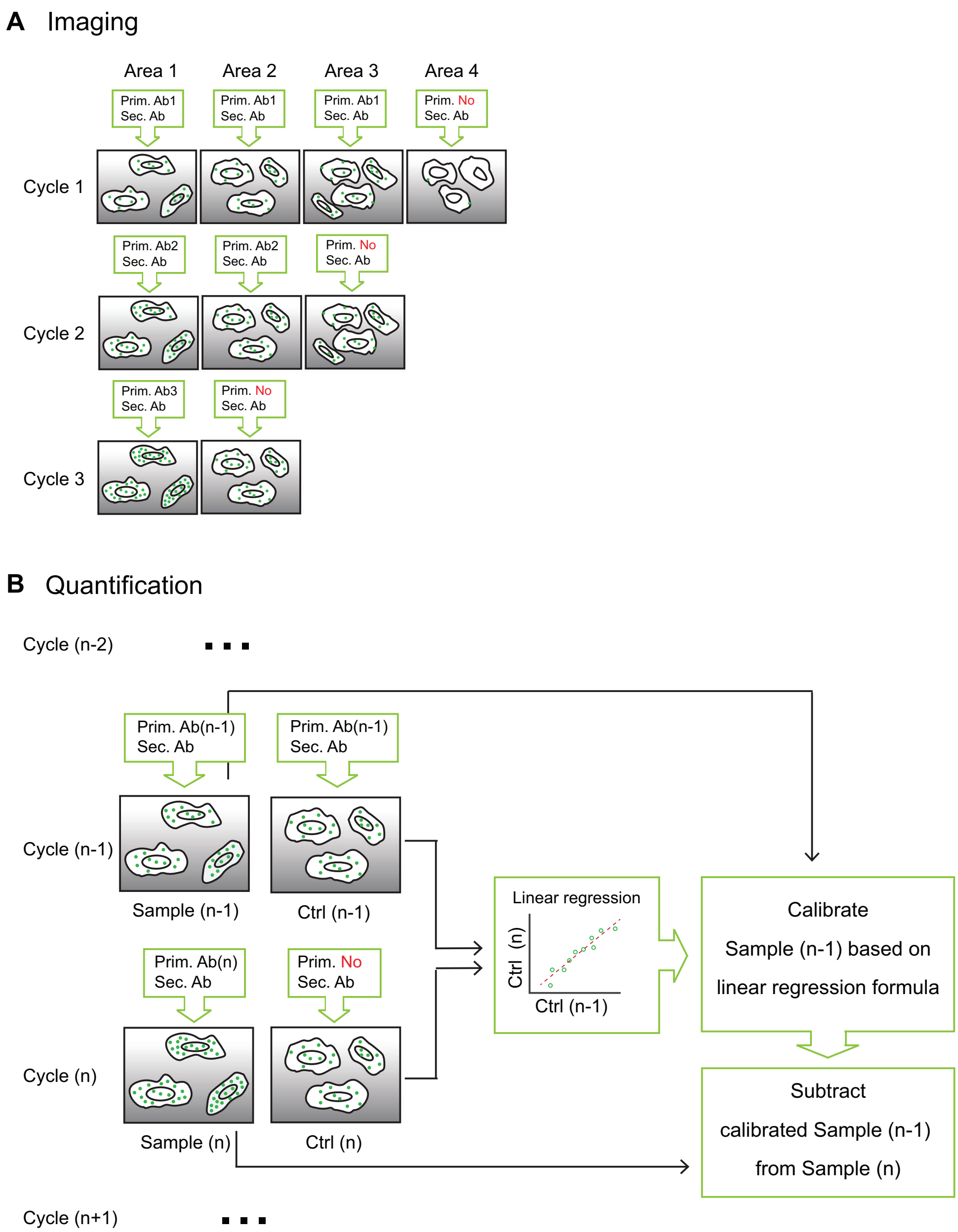

Figure EV1. Principles of cumulative microscopy.

(**A**) Schematic of the imaging setup. Each imaging cycle requires an extra staining in which no primary antibody is present. (**B**) Schematic of the quantification setup. Pixel values or quantification of segmented structures from the same area are compared for two imaging cycles. A scaling function is estimated by linear regression for the area in which no new primary antibody is introduced (Ctrl). The scaling function is used to calibrate the image or measurements from the sample before it can be subtracted from the image or measurements of the following cycle.

 © The Author(s)

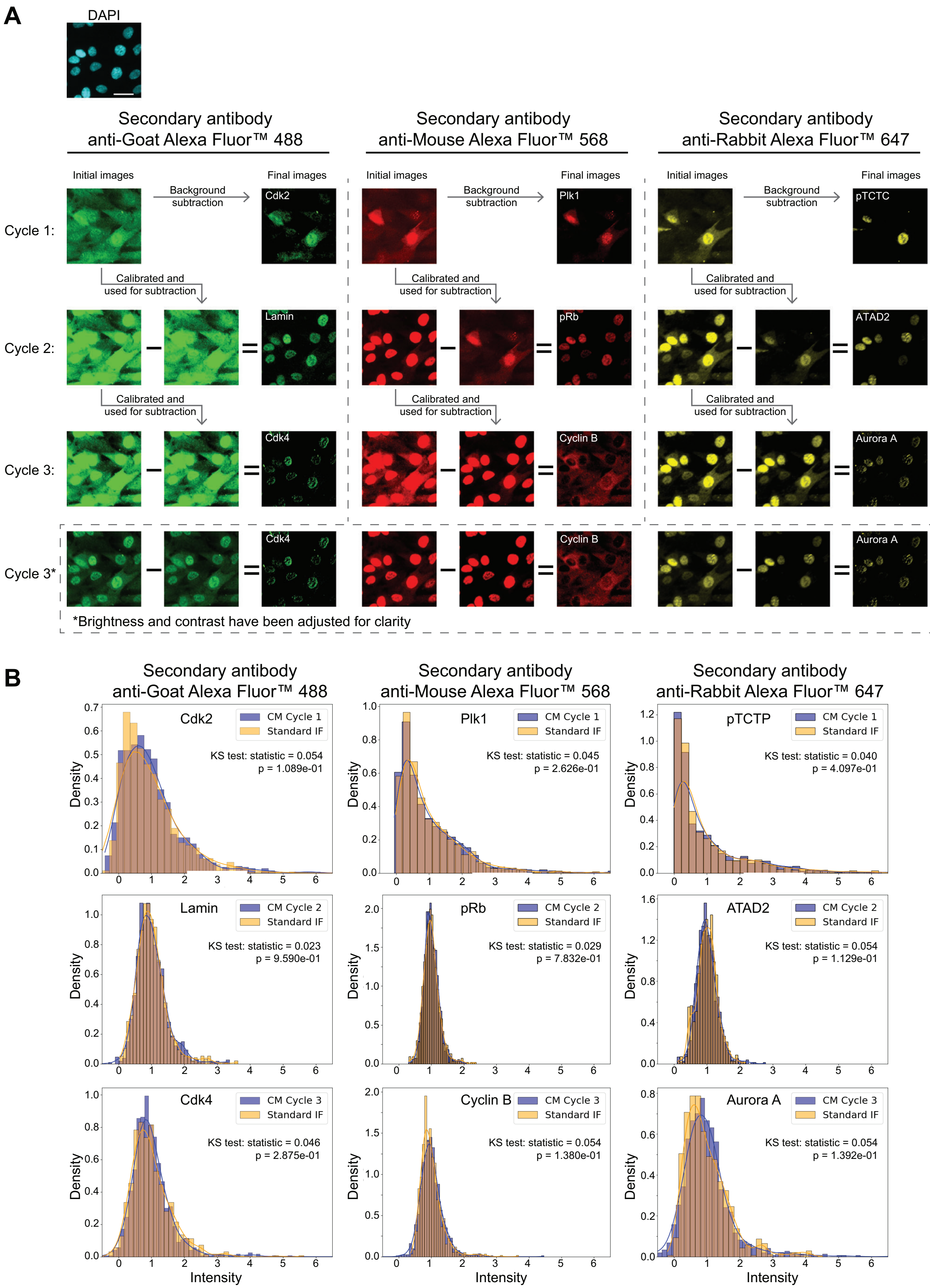
A
DAPI
Secondary antibody anti-Goat Alexa Fluor™ 488
Secondary antibody anti-Mouse Alexa Fluor™ 568
Secondary antibody anti-Rabbit Alexa Fluor™ 647
Initial images
Background subtraction
Final images
Cycle 1:
Cycle 2:
Cycle 3:
Cycle 3*
Calibrated and used for subtraction
Cdk2
Lamin
Cdk4
Plk1
pRb
Cyclin B
pTCTC
ATAD2
Aurora A
*Brightness and contrast have been adjusted for clarity
B
Cdk2
CM Cycle 1
Standard IF
KS test: statistic = 0.054 p = 1.089e-01
Plk1
KS test: statistic = 0.045 p = 2.626e-01
pTCTP
KS test: statistic = 0.040 p = 4.097e-01
Lamin
CM Cycle 2
KS test: statistic = 0.023 p = 9.590e-01
pRb
KS test: statistic = 0.029 p = 7.832e-01
ATAD2
KS test: statistic = 0.054 p = 1.129e-01
Cdk4
CM Cycle 3
KS test: statistic = 0.046 p = 2.875e-01
Cyclin B
KS test: statistic = 0.054 p = 1.380e-01
Aurora A
KS test: statistic = 0.054 p = 1.392e-01
Density
Intensity

Figure EV2. Workflow for cumulative microscopy.

(**A**) Example images for cumulative staining with Cdk2, Lamin, Cdk4, Plk1, pRb, Cyclin B, ATAD2, Aurora A, and pTCTP antibodies. Scale bar 40 μm. (**B**) Comparison of signal intensity distributions between Standard IF and three sequential cycles of Cumulative IF in RPE G2 cells. Each Cumulative IF cycle included simultaneous staining with three secondary antibodies: anti-Goat Alexa Fluor™ 488, anti-Mouse Alexa Fluor™ 568, and anti-Rabbit Alexa Fluor™ 647. Statistical differences in signal distributions were evaluated using the Kolmogorov–Smirnov (KS) test. Data shown are representative of three independent experiments, each performed with technical replicates.

 © The Author(s)

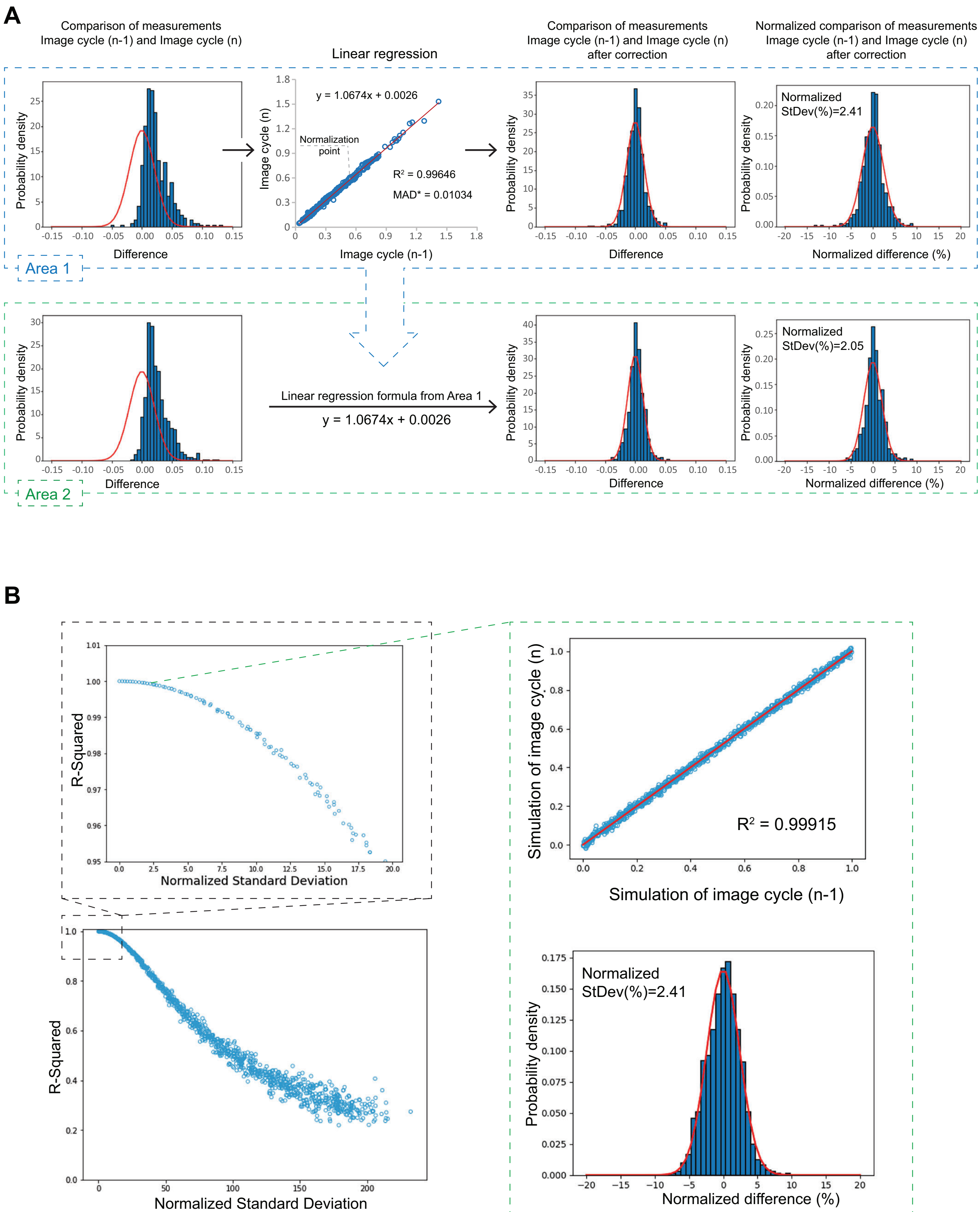
A
Comparison of measurements
Image cycle (n-1) and Image cycle (n)
Linear regression
Comparison of measurements
Image cycle (n-1) and Image cycle (n)
after correction
Normalized comparison of measurements
Image cycle (n-1) and Image cycle (n)
after correction
Probability density
Difference
y = 1.0674x + 0.0026
Normalization point
R² = 0.99646
MAD* = 0.01034
Image cycle (n)
Image cycle (n-1)
Normalized StDev(%)=2.41
Normalized difference (%)
Area 1
Linear regression formula from Area 1
y = 1.0674x + 0.0026
Normalized StDev(%)=2.05
Area 2
B
R-Squared
Normalized Standard Deviation
Simulation of image cycle (n)
Simulation of image cycle (n-1)
R² = 0.99915
Normalized StDev(%)=2.41
Probability density
Normalized difference (%)

Figure EV3. Estimation of uncertainty introduced by cumulative microscopy.

(**A**) Histograms show a comparison between measurements from two consecutive imaging cycles in which no new primary antibody is introduced. The red line shows a normal distribution centered at 0 with the standard deviation derived from the data as a comparison. Left, comparison of raw data. Middle, linear regression, and the resulting distribution after correction by the scaling function. Right, the same data after normalization to signal strength. Lower graphs show regions from a second well, using the scaling function derived from the first well. *The mean absolute deviation (MAD) is used to represent the average distance between each data value and the regression line. (**B**) Simulation of the relation between the R-square value and the normalized standard deviation.

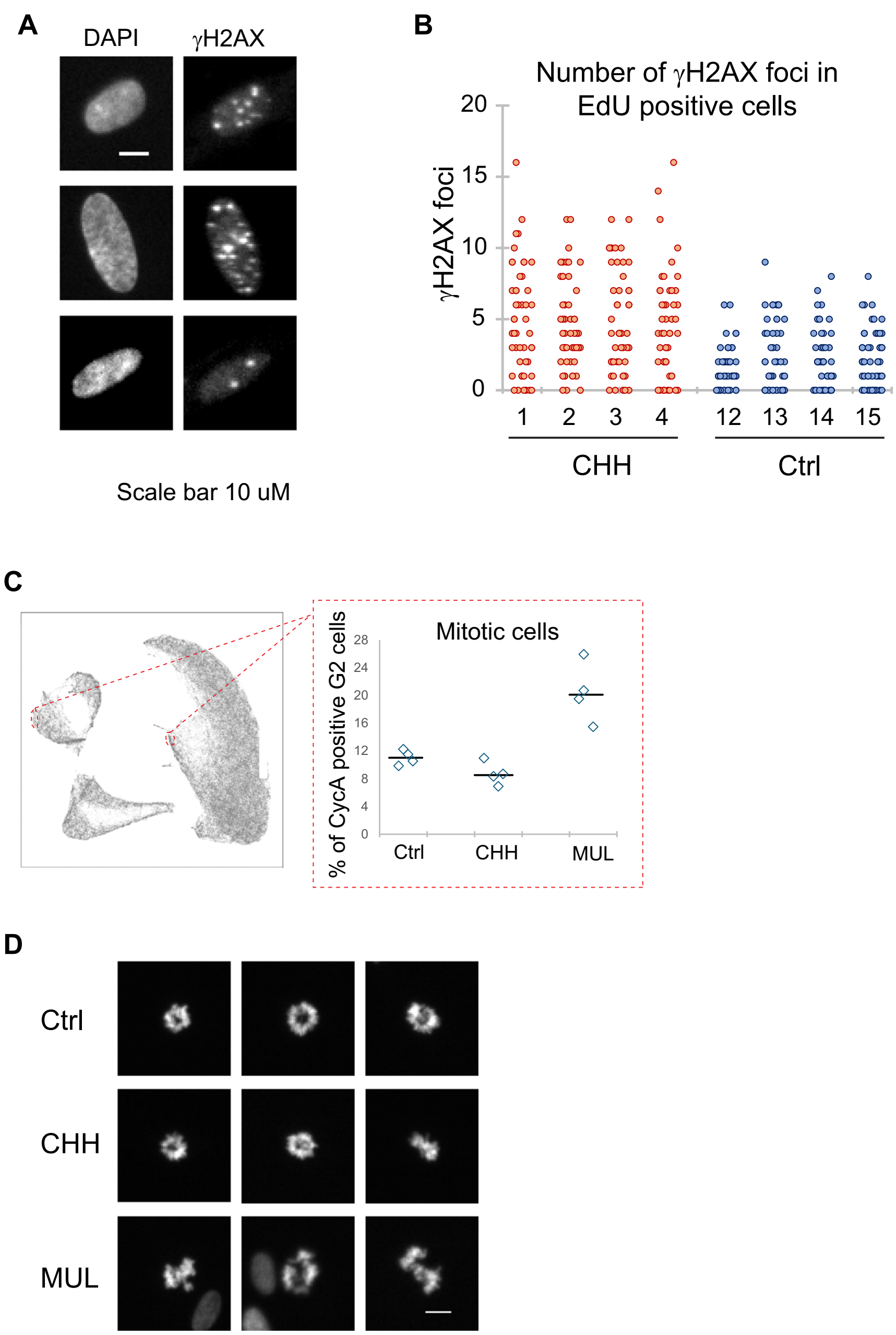

Figure EV4. Quantification of γH2AX foci and mitotic cells.

(**A**) Representative images for DAPI (left column) and γH2AX (right column) staining. Scale bar 10 μm. (**B**) Quantification of γH2AX foci in EdU-positive CHH (red) vs corresponding Ctrl (blue) cells. (**C**) Gate in UMAP to depict mitotic cells (left). Percentage of mitotic cells for Ctrl, CHH, and MUL in relation to all cells (middle) and Cyclin A positive G2 cells (right). (**D**) Representative images for DAPI staining in mitosis for Ctrl, CHH, and MUL cells. Scale bar 10 μm.

 © The Author(s)

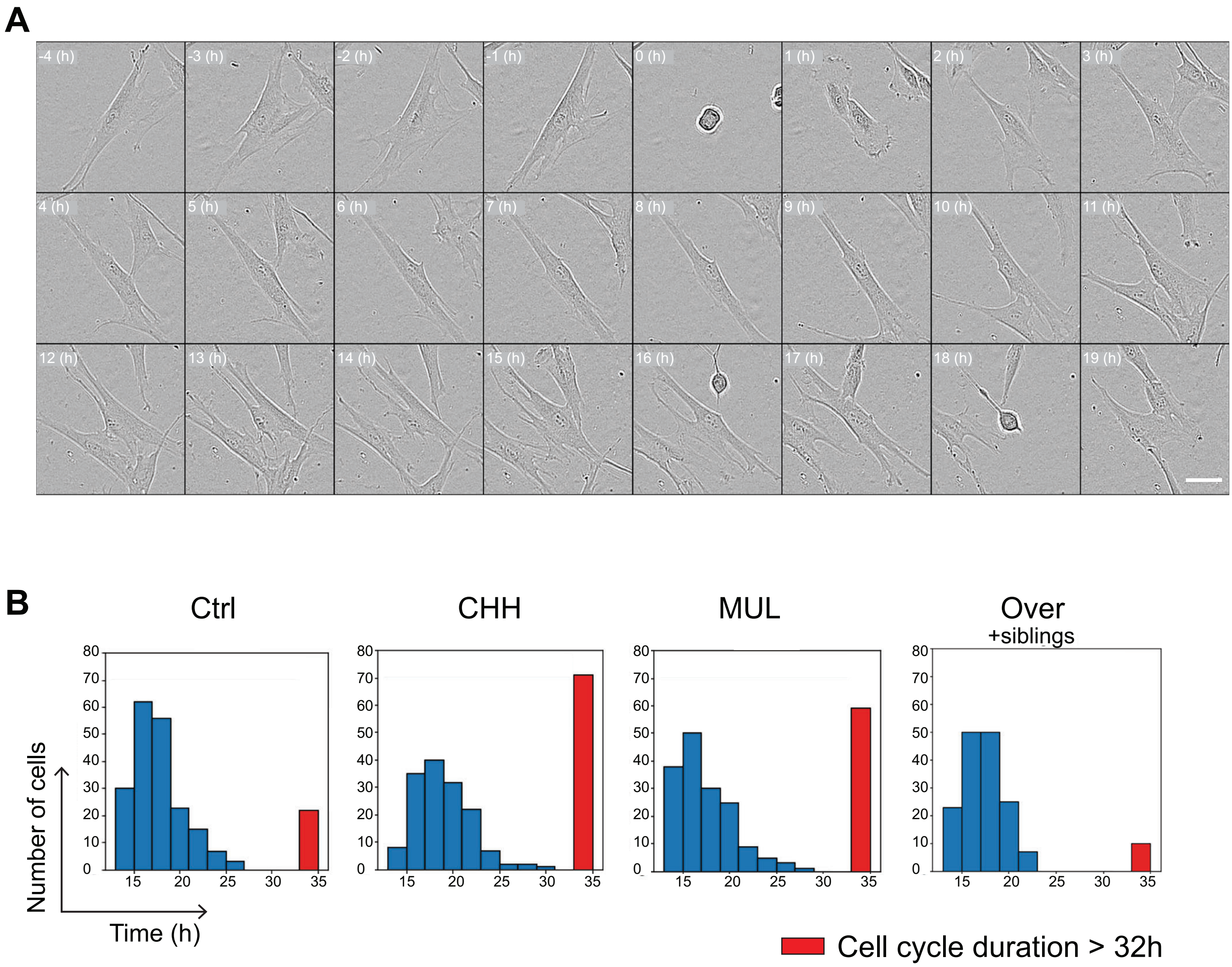

Figure EV5. Time-lapse imaging of patient-derived fibroblasts.

(**A**) Example of time-lapse images following a single cell. Scale bar 40 µm. (**B**) Quantification of the time between mitosis for the indicated cells. Cells for which the duration between mitoses was longer than 32 h, or two mitoses were not observed, are shown in red. Please note that "Over" here refers to both cells derived from the patient with overgrowth syndrome and the patient's siblings. Data shown are representative of two independent experiments, each performed with technical replicates.

© The Author(s)
